# Encapsulation Enhances the Catalytic Activity of C‐N Coupling: Reaction Mechanism of a Cu(I)/Calix[8]arene Supramolecular Catalyst

**DOI:** 10.1002/cctc.202200662

**Published:** 2022-09-01

**Authors:** Radu A. Talmazan, J. Refugio Monroy, Federico del Río‐Portilla, Ivan Castillo, Maren Podewitz

**Affiliations:** ^1^ Institute of Materials Chemistry TU Wien Getreidemarkt 9 1060 Vienna Austria; ^2^ Institute of General, Inorganic, and Theoretical Chemistry and Center of Molecular Biosciences University of Innsbruck Innrain 80/82 6020 Innsbruck Austria; ^3^ Instituto de Química Universidad Nacional Autónoma de México Circuito Exterior CU, Ciudad de México 04510 México; ^4^ Present address: Department of Chemistry Humboldt Universität zu Berlin Brook-Taylor-Strasse 2 12489 Berlin Germany

**Keywords:** C−N Coupling, Copper, Multiscale Modelling Supramolecular Catalysis

## Abstract

Development of C−N coupling methodologies based on Earth‐abundant metals is a promising strategy in homogeneous catalysis for sustainable processes. However, such systems suffer from deactivation and low catalytic activity. We here report that encapsulation of Cu(I) within the phenanthroyl‐containing calix[8]arene derivative 1,5‐(2,9‐dimethyl‐1,10‐phenanthroyl)‐2,3,4,6,7,8‐hexamethyl‐*p*‐tert‐butylcalix[8]arene (**C_8_PhenMe_6_**) significantly enhances C−N coupling activity up to 92 % yield in the reaction of aryl halides and aryl amines, with low catalyst loadings (2.5 % mol). A tailored multiscale computational protocol based on Molecular Dynamics simulations and DFT investigations revealed an oxidative addition/reductive elimination process of the supramolecular catalyst [**Cu(C_8_PhenMe_6_)I**]. The computational investigations uncovered the origins of the enhanced catalytic activity over its molecular analogues: Catalyst deactivation through dimerization is prevented, and product release facilitated. Capturing the dynamic profile of the macrocycle and the impact of non‐covalent interactions on reactivity allows for the rationalization of the behavior of the flexible supramolecular catalysts employed.

## Graphical abstract

In a combined theoretical and experimental study, we report how calix[8]arene encapsulation enhances the catalytic activity for C−N coupling of an Earth‐abundant Cu‐phenanthroline catalyst by prevention of deactivation through dimerization and by promotion of product release.

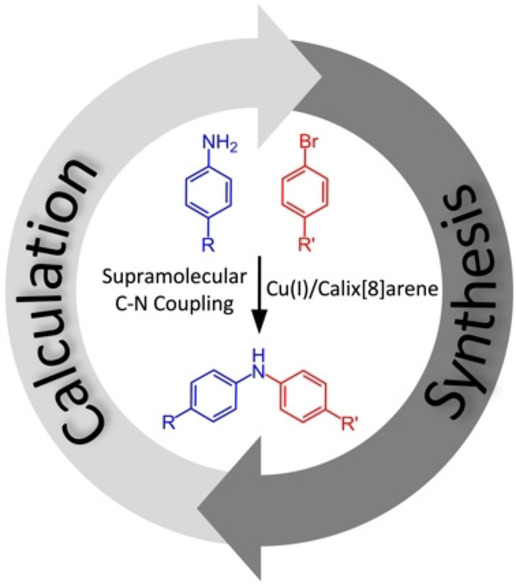

## Introduction

C−N bond formation is a reaction with a large demand, as amine derivatives are essential building blocks for multiple biologically active molecules,^[1]^ as well as play a key role in the design of high‐performance materials.^[2]^ In this regard, the development of novel, highly efficient catalysts based on Earth‐abundant metals, such as copper or iron, is an essential strategy to explore environmentally more benign and sustainable production processes.

In recent years, computational chemistry has played a fundamental role in the development of new catalysts by uncovering reaction mechanisms and rationalizing reactivity and selectivity.^[3]^ In fact, quantum chemical investigations have successfully been used not only to outline a mechanism, but also to understand the steric and electronic factors that govern catalytic systems, and to establish structure‐activity relationships.^[4]^ Consequently, complementing experimental efforts, computational studies can delineate the underlying mechanisms of the homogenous transition‐metal catalyzed C−N bond formation – a crucial step to evolve from the main strategies based on palladium,^[5]^ to extensions that employ Earth‐abundant metals like copper.^[6]^ However, appropriate computational protocols, adapted to the nature of the specific system, to obtain reliable results are required.

As Earth‐abundant systems such as those with copper are prone to dimerization and other deactivation patterns, including oxidation from Cu(I) to Cu(II), recent efforts to incorporate it as catalytic center have resorted to bioinspired supramolecular catalysis.^[7]^ Encapsulation of transition metals is a general strategy where a confined space or cavity provides a constrained chemical environment for the active species, while also imposing steric restrictions during the catalytic transformation.^[8]^ The cavity allows efficient collisions between substrates and the anchored metal site, creating a high density of local interactions that usually results in beneficial effects such as higher activity and selectivity, imitating the behavior of metalloenzymatic active sites.^[9]^

In this context, calix[n]arenes (n=4–8) represent a group of cavitands that has been used as hosts for different metal centers including copper, aimed at emulating enzymatic catalysis.^[10]^ However, the small calix[4,6]arenes tend to promote catalytic transformations that occur outside their cavities due to the limited confined space. Prior studies suggest that *p*‐*tert*‐butylcalix[8]arene (**C_8_**) is an outstanding option for supramolecular catalysis.[^11^, ^12^] Its larger and flexible cavity makes it amenable for metal ion encapsulation by using a coordinative moiety, while simultaneously allowing metal‐substrate interactions inside its cavity. Despite these potential advantages, the number of catalytic systems based on **C_8_** is significantly lower than those reported with the smaller calix[4,6]arenes.^[11]^ One reason that hindered the rational development of supramolecular systems is the lack of an adequate cost‐efficient computational methodology that sheds light on the intricate interplay of cavity conformations and active center accessibility. Such a methodology requires a description of the reaction mechanism at the catalytic center with electronic structure theory, while also incorporating the potentially vast conformational space of the macrocyclic ligand.

**Scheme 1:**
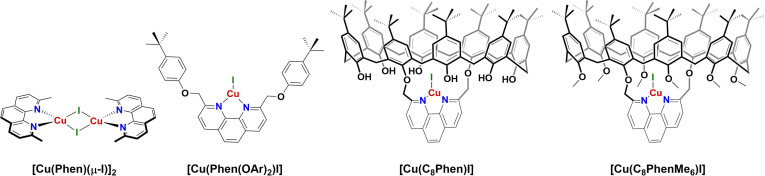
Molecular pre‐catalysts [**Cu(Phen)(μ‐I)**]_2_ and [**Cu(Phen(OAr)_2_)I**], as well as the parent supramolecular system **C_8_**‐based [**Cu(C_8_Phen)I**], and the new methylated system [**Cu(C_8_PhenMe_6_)I**].

A promising candidate for macrocyclic encapsulation with calix[8]arene is [**Cu(Phen)(μ‐I)**]_2_ (Phen=2,9‐dimethyl‐1,10‐phenanthroline, Scheme 1). Its monomeric form has been shown to be active towards C−N coupling, even though with high ligand and metal loadings (10‐20 % mol), and only the most active substrates were converted with acceptable yields.^[13]^ On the other hand, encapsulating Cu(I) with a bidentate phenanthroyl moiety in the recently reported **C_8_**‐based cavitand 1,5‐(2,9‐dimethyl‐1,10‐phenanthroyl)‐*p*‐*tert*‐butylcalix[8]arene (**C_8_Phen**)^[14]^ allows for assembly of the catalytic system [**Cu(C_8_Phen)I**] (Scheme 1). This supramolecular system exhibits remarkable catalytic C−S cross‐coupling capability.^[12]^ Consequently, exploring **C_8_**‐based encapsulation of Cu‐phenanthroline complexes for C−N coupling reactions is an evident choice to develop highly efficient catalysts and to expand the range of reactions.

For a rational development of more efficient catalysts, insight into the underlying reaction mechanism is essential. C−N coupling reactions are known to follow different reaction pathways, involving either a single electron transfer (SET), an atom transfer mechanism with charged species, or an oxidative addition/reductive elimination sequence (see e. g., Ref 15a). These mechanistic possibilities have been investigated with computational methods, as well as experiments, with varying results.[^3a^, ^15^, ^16^] It appears that the nature of the catalyst, as well as the substrate, dictate the pathway,^[16]^ with phenanthroline‐based catalysts and aryl substrates generally following an oxidative addition/reductive elimination path.^[16d]^ To identify the most viable mechanism, all alternatives need to be carefully evaluated in experimental and theoretical investigations.

In this context, the computational modelling of supramolecular systems including large calixarenes is not straightforward due to their intrinsic flexibility and hence, conformational diversity.^[3e]^ This aspect must not be neglected to accurately assess the conformational space available to these types of catalysts. However, conformer sampling is not mature in transition‐metal catalysis and even less established when flexible macrocyclic ligands are involved. The reason for it is the inherent difficulty to describe such compounds with standard conformer generators, and tailor‐made approaches must be developed for these systems. To that end, enhanced sampling techniques such as accelerated molecular dynamics have proven successful to capture the conformational space of macrocycles, and this approach will be exploited here.^[17]^

Thus, we describe how encapsulation within a macrocyclic **C_8_** cavitand significantly increases the C−N coupling activity of a Cu‐phenanthroyl‐derived catalyst, with CuI as an inexpensive and abundant metal source to assemble the supramolecular pre‐catalyst [**Cu(C_8_PhenMe_6_)I**] (**C_8_PhenMe_6_** =1,5‐(2,9‐dimethyl‐1,10‐phenanthroyl)‐2,3,4,6,7,8‐hexamethyl‐*p*‐tert‐butyl‐calix[8]arene, Scheme 1). The experimental results are corroborated with a tailored multiscale computational protocol to elucidate the reaction mechanism of C−N bond formation, mediated by [**Cu(C_8_PhenMe_6_)I**], and to determine the origin of its higher catalytic activity when compared to the molecular analogue [**Cu(Phen)(μ‐I)**]_2_. Our modelling approach is based on enhanced sampling techniques and quantum chemical investigations, while computational results are validated and complemented by extensive experimental characterization and activity studies.

## Results and Discussion

### Synthesis and Characterization

**Figure 1:**
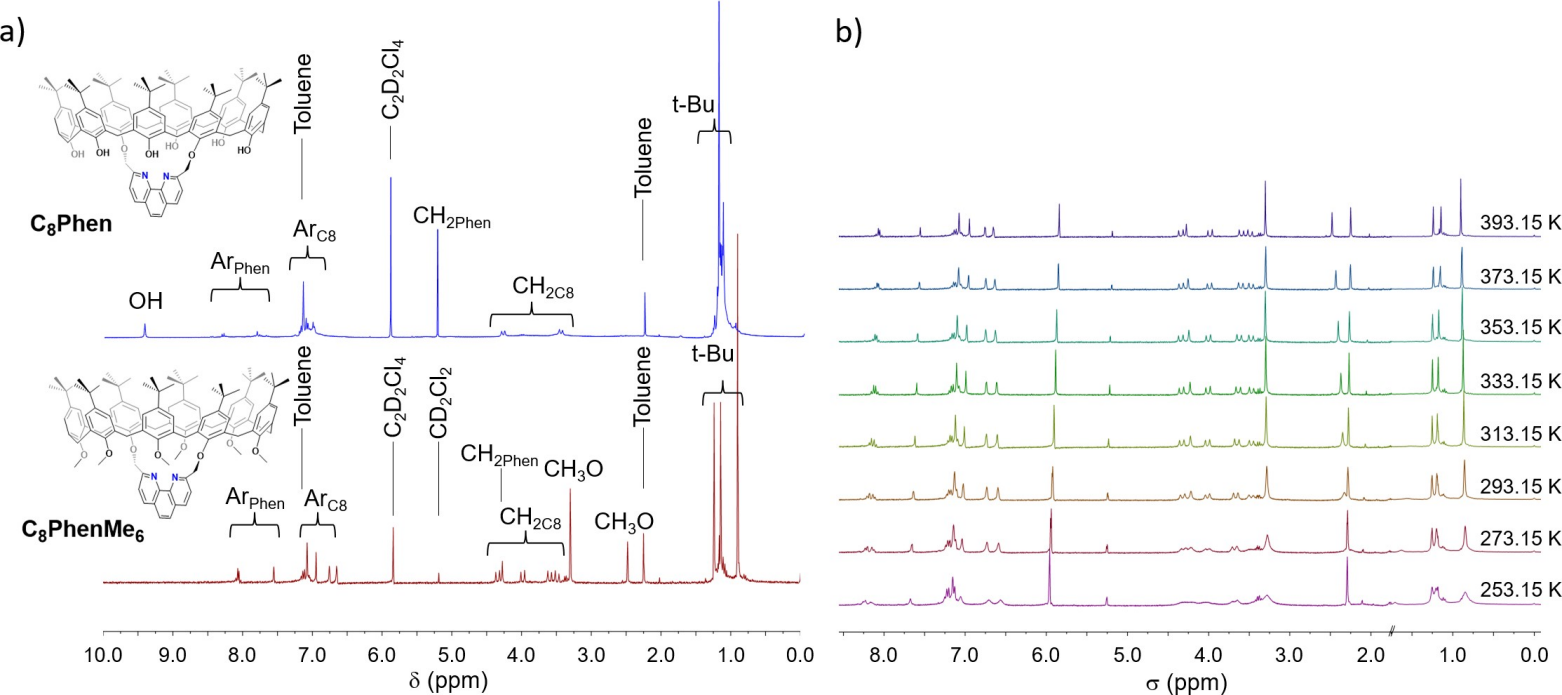
a) Comparison of ^1^H NMR spectra of the parent cavitand **C_8_Phen** (blue trace) and the new cavitand **C_8_PhenMe_6_** (red trace) in C_2_D_2_Cl_4_. b) VT ^1^H NMR spectra of **C_8_PhenMe_6_** in C_2_D_2_Cl_4_ from 253.15 to 393.15 K.

In the new cavitand **C_8_PhenMe_6_**, the hydroxy groups from the parent **C_8_Phen** were substituted by methoxy groups, which do not participate in acid‐base equilibria under the basic conditions required for catalytic C−N coupling.^[18]^ Complete methylation of the hydroxy groups of **C_8_Phen** was achieved with a slight excess of MeI after deprotonation with NaH in anhydrous THF. The ^1^H NMR spectrum of **C_8_PhenMe_6_** does not display signals beyond *δ* 8.50 ppm, which are associated with hydroxy groups in calix[8]arenes (Figure 1a).[^18^, ^19^] Instead, the presence of two new singlets at *δ* 2.47 (6H, MeO_int_) and 3.30 ppm (12H, MeO_ext_) attest to the −OMe substitution. The methoxy groups are labelled as MeO_int_, for those facing inside the calixarene, and MeO_ext_, for those outside, based on the shielding or deshielding caused by the aromatic groups. Full NMR spectroscopic analysis of **C_8_PhenMe_6_** is presented in the Electronic Supporting Information (SI Figures S1–S4), including COSY NMR that allowed the assignment of signals corresponding to the methylene bridges of the cavitand CH_2C8_ at *δ* 3.49 ppm (d, 4H, CH_2C8(d)_), 3.59 (d, 4H, CH_2C8(b)_), 3.98 (d, 4H, CH_2C8(a)_), and 4.34 (d, 4H, CH_2C8(c)_), while the methylene connector to the phenanthroyl moiety was detected at 4.27 ppm (s, 4H, CH_2Phen_). Assignment of the signals was facilitated by Variable Temperature (VT) ^1^H NMR spectroscopy (Figure 1b). The characteristic three‐singlet pattern for the *tert*‐butyl groups of a 1,5‐substituted calix[8]arene was clearly discernible at *δ* 1.23, 1.14, and 0.90 ppm with a 1 : 1 : 2 ratio above 353 K; minor peaks likely correspond to different conformers (see computational section). The aromatic signals corresponding to the phenanthroyl moiety are well resolved at high temperature, being clearly identified at *δ* 8.06 and 7.55 ppm. The signals corresponding to the cavitand phenolic framework (Ar_C8_) were observed from *δ* 7.55 to 6.65 ppm. The IR spectrum of the new cavitand in Figure S5 does not show bands above 3315 cm^−1^ that may be assigned to ν_O‐H_, further supporting the complete methylation of the hydroxy groups. Positive‐ion Fast Atom Bombardment Mass Spectrometry (FAB^+^ MS) in Figure S6 shows an intense peak at *m/z*=1586, assigned to the protonated molecular ion [**C_8_PhenMe_6_H**]^+^. Finally, addition of CuI to an equimolar amount of **C_8_PhenMe_6_** in toluene suspension resulted in pre‐catalyst [**Cu(C_8_PhenMe_6_)I**].

**Figure 2:**
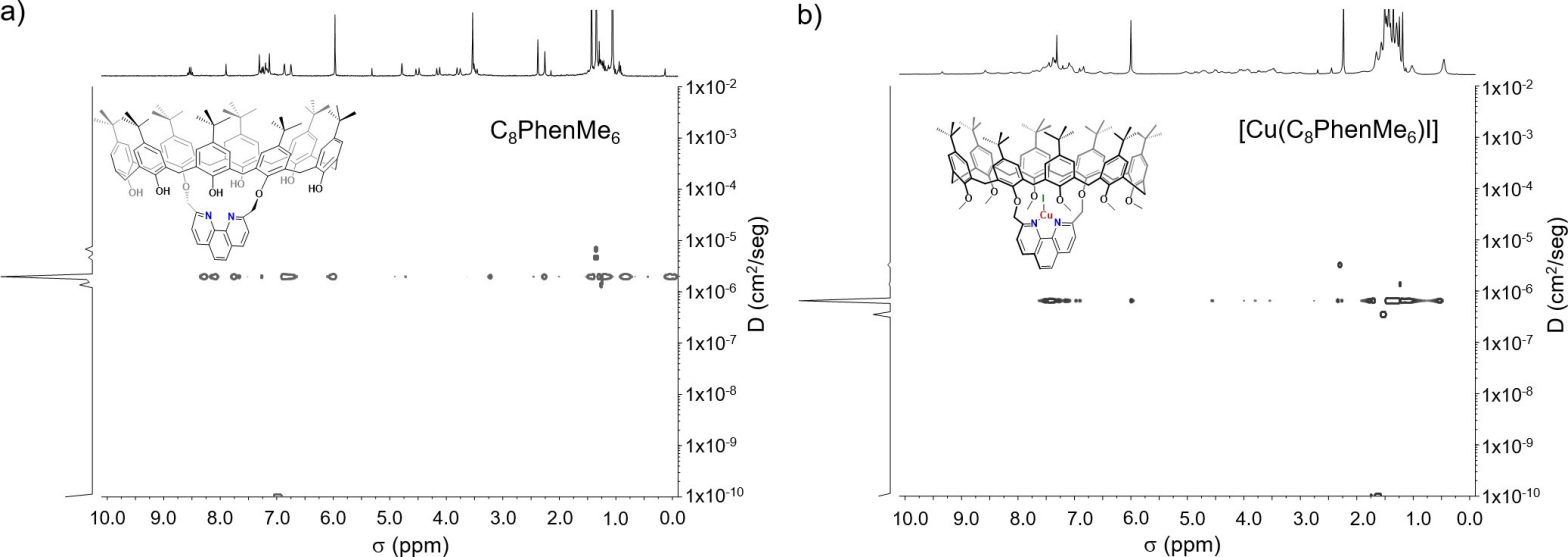
a) ^1^H 500 MHz DOSY spectrum of 8 mM **C_8_PhenMe_6_** in 1,1,2,2‐tetrachloroethane‐*d_2_* |toluene‐*d_8_* mixture (500|50 μL) at 298 K, diffusion=6.49×10^−7^ cm^2^ s^−1^, r_H_=24.90 Å; b) ^1^H DOSY spectrum of 8 mM [**Cu(C_8_PhenMe_6_)I**] in 1,1,2,2‐tetrachloroethane‐*d_2_* |toluene‐*d_8_* at 298 K, diffusion=6.49×10^−7^ cm^2^ s^−1^, r_H_=24.90 Å.

**Figure 3:**
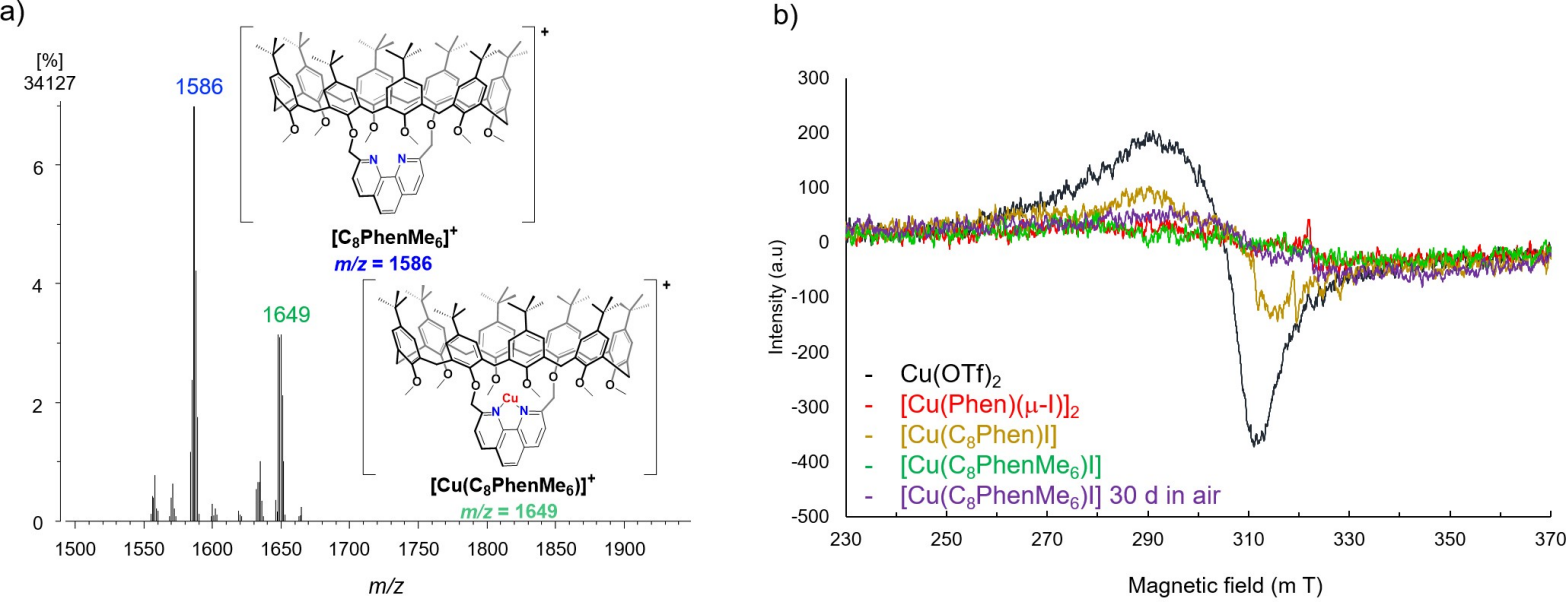
a) Experimental FAB^+^ MS of [**Cu(C_8_PhenMe_6_)I**] showing peaks assigned to [**C_8_PhenMe_6_**]^+^ and [**Cu(C_8_PhenMe_6_)**]^+^; b) X‐band EPR spectra of toluene suspensions of [**Cu(C_8_PhenMe_6_)I**] and reference compounds at 77 K.

Characterization of [**Cu(C_8_PhenMe_6_)I**] was initially established by ^1^H NMR spectroscopy and aided by VT ^1^H NMR studies (Figures S7–S9 in the SI). The compound exhibits a symmetry plane as the free cavitand **C_8_PhenMe_6_**, based on the three *tert*‐butyl signals in a 1 : 1 : 2 ratio, and the four doublets corresponding to the calix[8]arene methylene bridges CH_2C8_ at *δ* 3.75 (m, 4H, CH_2C8(a)_), 3.81 (m, 4H, CH_2C8(d)_), 4.18 (m, 4H, CH_2C8(b)_), and 4.21 (d, *J*=16 Hz, 4H, CH_2C8(c)_) observed at 393 K. The monomeric nature of [**Cu(C_8_PhenMe_6_)I**] was confirmed by DOSY ^1^H NMR spectroscopy (Figure 2), where the free cavitand **C_8_PhenMe_6_** (Figure 2a) and the complex [**Cu(C_8_PhenMe_6_)I**] (Figure 2b) exhibit identical diffusion coefficients and hydrodynamic radii (r_H_=24.90 Å).^[20]^ This observation suggests that the average size of **C_8_PhenMe_6_** is retained after CuI is anchored, indicating that **C_8_PhenMe_6_** may play a dual role as ligand: Confining the metal center (and potential substrates) within the cavity, while simultaneously preventing dimerization that deactivates other phenanthroline‐based pre‐catalysts (see also computational investigations, *vide infra*). Confirmation of the proposed identity of [**Cu(C_8_PhenMe_6_)I**] was established by FAB^+^ MS, with a peak at *m/z*=1649 assigned to [**Cu(C_8_PhenMe_6_)**]^**+**^ (Figure 3a). Complex formation is facilitated by the lability of the Cu(I) ion,^[21]^ with FAB^+^ MS obtained after 30 min of stirring at 25 °C being identical to that displayed in Figure 3a. Furthermore, air stability of the pre‐catalyst was confirmed through EPR spectroscopic studies at X‐band frequency. [**Cu(C_8_PhenMe_6_)I**] is EPR‐silent as expected for a diamagnetic Cu(I) *d* ^10^ species (Figure 3b), and exposure of the isolated solid to air for 30 days resulted in virtually no change. In contrast, exposure to air for a few minutes resulted in significant signal intensity for [**Cu(C_8_Phen)I**], consistent with oxidation to the EPR active Cu(II) species. [**Cu(Phen)(μ‐I)**]_2_ appears to be protected towards oxidation due to its dimeric nature;^[22]^ Cu(OTf)_2_ was employed as reference (OTf^−^=trifluoromethanesulfonate), see spectra in Figure 3b.

### Catalytic Proof of Concept

As proof of concept to test the properties of the new cavitand **C_8_PhenMe_6_** in combination with Cu(I), the C−N coupling catalytic activity of the system was evaluated with 2.5 % loading of each. Aniline and bromobenzene in equimolar amounts were chosen as substrates as benchmark test; toluene was used as solvent, avoiding the commonly employed and high‐boiling dimethylsulfoxide. Initial assessment of the reaction was carried out with several bases, KO*t*Bu affording the best results, as shown in Tables 1 and S1 in the SI. In a second set of evaluations, different sources of Cu(I) were tested to form the **C_8_PhenMe_6_**/Cu(I) catalytic system, with CuI performing better than CuBr or CuCl. Subsequently, the aryl halide substrate was varied, revealing that [**Cu(C_8_PhenMe_6_)I**] discriminates substrate by size: A significantly better performance was achieved for the production of diphenylamine (product **a**) when bromobenzene (PhBr) was used, rather than iodobenzene (PhI). Although iodide acts as a better leaving group that facilitates the potentially rate‐limiting oxidative addition step (*vide infra*),^[23]^ PhBr was determined to be a better substrate in related C−S couplings catalyzed by [**Cu(C_8_Phen)X**] (X=Cl, I).^[12]^ The isolated yield of product **a** with PhBr (73 %, entry 2 in Table 1) is significantly better than with PhI (56 %, entry 3 in Table 1). This result supports the notion that the substrates must access the confined space of the calixarene cavity to interact with the Cu(I) center. Thus, the rate of C−N coupling must be controlled by this steric restriction in combination with ease of oxidative addition of the aryl halide, since chlorobenzene (PhCl) did not react (Table 1, entry 1). Methylation of the phenolic moieties is key to achieve high yields of C−N coupling products, since [**Cu(C_8_Phen)I**] afforded only 50 % of product **a** (entry 4 in Table 1). This may be attributed to the potential interference of deprotonated ArOH groups of **C_8_Phen** in the presence of the strong base KO*t*Bu. Although a reasonable amount of **a** was obtained (50 %), collateral formation of homocoupling products of aryl halide (biphenyl, product **b**) and aniline (azobenzene, product **c**) were observed in low (7–12 %) yields. The formation of **b** is a commonly observed secondary catalytic path in Cu(I)‐based systems,^[24]^ particularly since KO*t*Bu can also promote biaryl coupling reactions.^[25]^ Aniline oxidation to generate **c** has been associated with Cu(I) complexes in the presence of an oxidant.^[26]^ In this evaluation, the cavitand **C_8_Phen** does not seem to protect the Cu(I) center towards oxidation to Cu(II) by adventitious oxygen as its methylated counterpart **C_8_PhenMe_6_**. Thus, aniline oxidation to generate **c** may occur at the Cu(II) center, regenerating Cu(I). Acid‐base equilibria also affect the catalytic performance, since deprotonation of the hydroxy groups of **C_8_Phen** is likely favored over that of aniline (even when coordinated to Cu(I)) necessary for C−N coupling. The presence of Cu(II) in the **C_8_Phen** system was confirmed by EPR analysis as mentioned above, with approximately 20 % of [**Cu(C_8_Phe)I**] initially present oxidized to Cu(II) based on integration and comparison to the external standard Cu(OTf)_2_ (Figure 3b). In contrast, **C_8_PhenMe_6_** is more selective for C−N coupling, since no trace of azobenzene (**c**) formation was observed, and this may be due to the prevalence of Cu(I) as confirmed by EPR spectroscopic analysis of [**Cu(C_8_PhenMe_6_)I**]. Therefore, **C_8_PhenMe_6_** stabilizes the reduced species under strongly basic conditions, even upon exposure to air for 30 days, with a maximum of 5 % Cu(II) present based on integration of the incipient EPR signal. These observations confirm that the chemical environment provided by the **C_8_PhenMe_6_** cavity stabilizes Cu(I) and prevents alternative acid‐base equilibria that may take place within the OH‐containing **C_8_Phen**.

**Table 1:** Optimization of the reaction conditions for C−N cross‐coupling.^[a]^

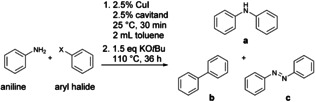					
entry	cavitand	aryl halide	yield^[b]^ [%]		
			**a**	**b**	**c**
1	**C_8_PhenMe_6_**	chlorobenzene	–	–	–
2	**C_8_PhenMe_6_**	bromobenzene	73	8	–
3	**C_8_PhenMe_6_**	iodobenzene	56	10	–
4^[c]^	**C_8_Phen**	bromobenzene	50	12	7
5^[d]^	**Phen**	bromobenzene	20	10	–
6^[d]^	**Phen(OAr)_2_**	bromobenzene	26	8	10
7^[e]^	none	bromobenzene	–	9	–
8^[f]^	**C_8_PhenMe_6_**	bromobenzene	68	6	–

[a] The evaluations were performed in a Schlenk flask (25 mL) with 2 mL of toluene, under dinitrogen atmosphere, temperature was raised to 110 °C in an oil bath for 36 h. Reaction conditions: molar ratio of 1 : 1 of aryl halide and aniline, with 2.5 % mol of cavitand and 2.5 % mol of CuI. [b] Isolated yields after column chromatography. [c] 8 % of triphenylamine (see Figures S20–21) isolated as double coupling product. [d] 10 % mol of ligand and 10 % mol of CuI. [e] without cavitand and 10 % mol of CuI. [f] Mercury drop test was performed.

To evaluate the influence of the cavity on catalytic performance, we compared the **C_8_PhenMe_6_**/CuI system with the molecular analogues **Phen** and 2,9‐dimethoxy(4‐*tert*‐butylphenyl)‐1,10‐phenanthroline (**Phen(OAr)_2_**). Although substoichiometric quantities of ligand and CuI were employed (10 % mol, entries 5 and 6, Table 1), no products were detected initially by thin layer chromatography (12‐24 h of reaction time), and only low yields of **a** were consistently obtained (<26 % at 36 h), further supporting the enhancement of C−N coupling activity due to macrocyclic encapsulation. The low conversion may be attributed to the known ligand redistribution tendency of **Phen** that results in complexes with 2 : 1 ligand/Cu(I) stoichiometry in solution.^[22]^ Indeed [**Cu(Phen)_2_**]^**+**^ was detected by Direct Analysis in Real Time (DART) MS at *m/z*=479 (Figure S10). Halogen ligands tend to favor Cu−X−Cu (X=Cl, Br, I) bridge formation that limit the availability of the Cu(I) centers for catalytic reactions,^[12]^ as evidenced by DOSY NMR spectroscopy of **Phen** in Figure S11 that affords a value of r_H_=2.38 Å *vs* that of [**Cu(Phen)(μ‐I)**]_2_ in Figure S12 that is more than double at r_H_=7.25 Å, consistent with a dimeric formulation. These experimental findings are in line with quantum chemical calculations, where dimerization yielding a Cu−X−Cu structural motif was energetically favored (see computational section of the results for more details).

In the case of **Phen(OAr)_2_** that also features the bidentate phenanthroline fragment, we hypothesized that the bulky substituents akin to those in **C_8_PhenMe_6_** may inhibit the dimerization observed with **Phen (**for DART MS of **Phen(OAr)_2_** see Figure S13, for ^1^H NMR characterization of **[Cu(Phen(OAr)_2_)]** see Figure S14). Nevertheless, DOSY NMR spectroscopy (Figures S15–S16) shows that the calculated hydrodynamic radius of the complex formulated as **[Cu(Phen(OAr)_2_)I]_2_** (r_H_=10.56 A) is about twice that of the free ligand **Phen(OAr)_2_** (r_H_=5.65 A). Furthermore FAB^+^ MS attests to the presence of the redistribution product **[Cu(Phen(OAr)_2_)_2_]^+^** at *m/z*=1071.5 (Figure S17), along with monometallic **[Cu(Phen(OAr)_2_)]^+^** at *m/z*=567. This implies that bulky substituents do not completely prevent ligand redistribution of the corresponding Cu(I) complex with **Phen(OAr)_2_**. Thus, the limited catalytic performance of [**Cu(Phen(OAr)_2_)I**] is likely due to equilibration with [**Cu(Phen(OAr)_2_)_2_**]^**+**^, and the absence of the confined space provided by the cavity in **C_8_PhenMe_6_**. The combined observations indicate that such cavity is necessary to obtain high yields of the C−N coupling products, likely by increasing the local concentration of substrates during the catalytic process. This would allow more efficient interactions of the substrates with the Cu(I) center. We also tested C−N coupling with substoichiometric quantities of CuI (10 % mol) in the absence of ligands (entry 7, Table 1), and no coupling product **a** was obtained. Finally, a mercury drop test did not alter the yield of **a** with [**Cu(C_8_PhenMe_6_)I**];^[27]^ although it does not provide definitive proof of a homogeneous catalytic process, we found no evidence of metallic copper.

### Substrate Scope

The scope of C−N coupling with [**Cu(C_8_PhenMe_6_)I**] was explored by using different bromobenzene derivatives, as shown in Table 2. As usually observed in these types of coupling reactions, electron‐deficient substrates result in higher yields of the desired products (entries 1–2 in Table 2). Moderate yields were obtained when electron‐rich substrates were tested (entries 3–5), and also when *orth*o substituents relative to the halide are present (see also entry 6). The overall results evidence the good catalytic performance of [**Cu(C_8_PhenMe_6_)I**], particularly when the catalyst loading is compared to that of related molecular systems, which typically ranges from 5 to 10 % mol.[^14^, ^24^] The use of 1‐allyloxy‐2‐iodobenzene as substrate afforded the expected coupling product, isolated in moderate yield (entry 6, 51 %). Nonetheless, we did not find evidence of the formation of the cyclic product expected when a radical mechanism is involved in C−N bond formation.^[24b]^ Thus, [**Cu(C_8_PhenMe_6_)I**] does not appear to promote the formation of aryl radical intermediates, see theoretical section for mechanistic details.

**Table 2:** Scope of the C−N cross‐coupling catalyzed by in situ formed [Cu(C_8_PhenMe_6_)I].^[a]^

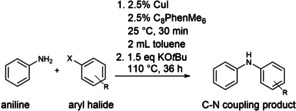			
entry	aryl halide	C−N coupling product	yield^[b]^ [%]
1	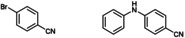		76
2	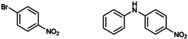		83
3	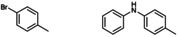		68
4	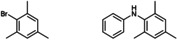		50
5	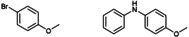		56
6	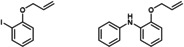		51

[a] Same conditions as in Table 1. [b] Isolated yields after column chromatography.

To gain further insight on the scope of the C−N reaction, different nitrogen‐based substrates were tested under the standard catalytic conditions (Table 3). The results show that ammonium acetate is not a good substrate. Also, γ‐valerolactam as a source of nitrogen reagent (entry 2, Table 3), offered no significant improvement in yield (76 %). In contrast, when a more nucleophilic and less sterically hindered reagent like imidazole was employed (entry 3, Table 3), a higher yield was obtained (82 %). Double substitution for the preparation of 2,6‐disubstituted pyridine‐bridged compound is also possible and resulted in an excellent yield (92 %), avoiding high‐boiling solvents or hazardous reaction conditions for this synthetic target.^[28]^ These results indicate that smaller amines are preferred, supporting the notion that the use of [**Cu(C_8_PhenMe_6_)I**] may result in predominant steric, rather than electronic control. For characterization of coupling products, see Figures S18–S43 in the SI.

**Table 3:** C−N coupling with different nitrogen sources with arylbromide substrates catalyzed by in situ formed [Cu(C_8_PhenMe_6_)I].^[a]^

entry	*N*‐source	C−N coupling product	yield^[b]^ [%]
1	NH_4_CH_3_CO_2_		–
2	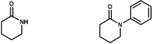		76
3	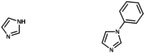		82
4^[c]^	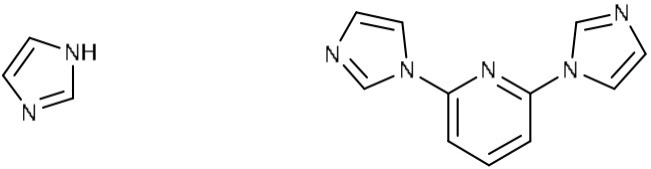		92

[a] Same conditions as in Table 1, using bromobenzene as substrate. [b] Isolated yields after column chromatography. [c] 2,6‐dibromopyridine was used as substrate with imidazole in 1 : 2 ratio.

### Computational Mechanistic Investigations

**Figure 4:**
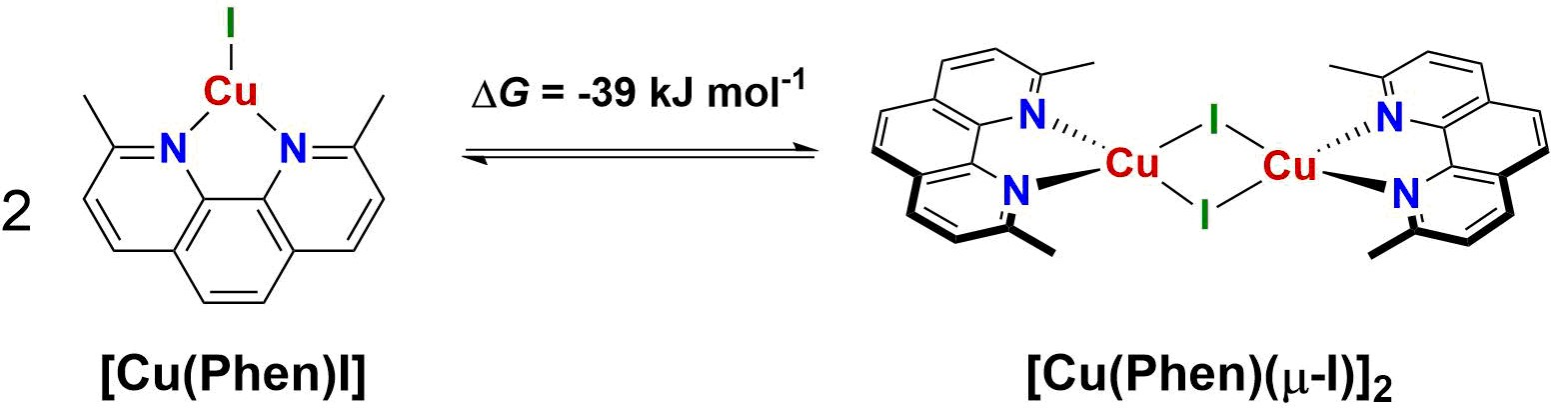
Dimerization of **[Cu(Phen)I]** to **[Cu(Phen)(μ‐I)]_2_**; reaction free energies were calculated with PBE0/def2‐TZVP/D3/COSMO//PBE0/def2‐SVP/D3/COSMO (for details see Computational Methodology).

To substantiate the experimental findings and shed light on the mechanism underlying the C−N coupling, detailed theoretical studies were carried out. Due to the size and the inherent flexibility of [**Cu(C_8_PhenMe_6_)I**], initial mechanistic studies were carried out on the simpler [**Cu(Phen)I**] (Figure 4, for a molecular structure see Figure S45) as model system. Although experimental evidence shows that this species is a poor catalyst, its low performance may be attributed to deactivation upon dimerization to [**Cu(Phen)(μ‐I)**]_2_ (compare Figure S46). Indeed, dispersion‐corrected density functional theory (DFT) investigations in implicit toluene (see Computational Methodology for details) predicted relative electronic and free energies to thermodynamically favor dimerization with Δ*E*=−71 kJ mol^−1^ and Δ*G* ^298^=−39 kJ mol^−1^ (Figure 4). Thus, dimerization appears to be one major factor for deactivation and investigation of [**Cu(Phen)I**] as model system is ideally suited to gain mechanistic insights prior to tackling the full‐fledged catalyst [**Cu(C_8_PhenMe_6_)I**].

**Figure 5:**
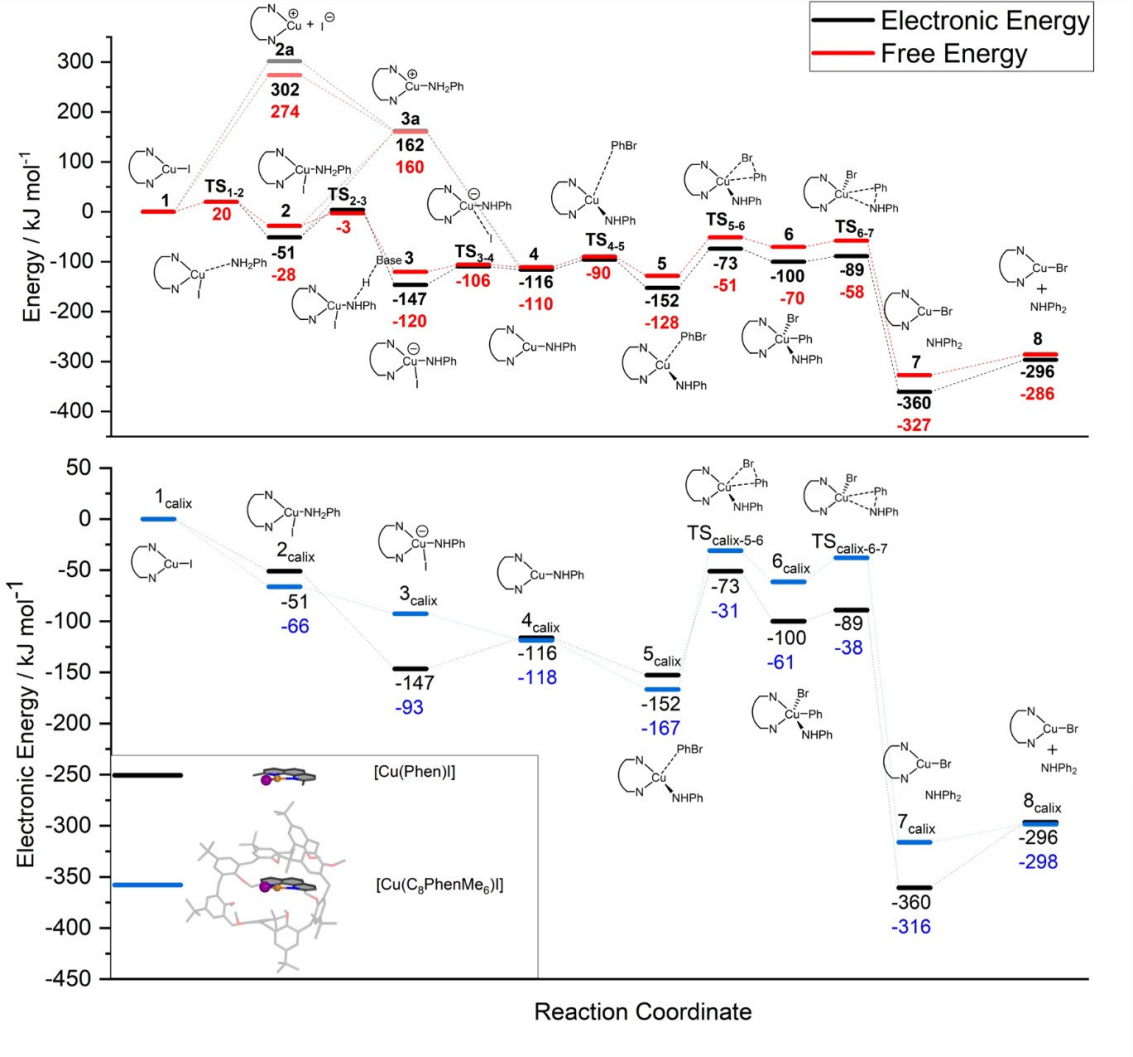
Top: Calculated energy diagram for the C−N coupling of aniline and PhBr with the model catalyst [**Cu(Phen)I**]. Relative electronic energies are given in black horizontal bars, whereas relative free energies are indicated by red bars. All energies are in kJ mol^−1^, and were calculated with PBE0/def2‐TZVP/D3/COSMO//PBE0/def2‐SVP/D3/COSMO. Bottom: Calculated energy diagram for the C−N coupling of aniline and PhBr with [**Cu(Phen)I**] (black bars) and [**Cu(C_8_PhenMe_6_)I**] (blue bars). All energies are relative electronic energies in kJ mol^−1^, and were calculated with PBE0/def2‐TZVP/D3/COSMO//PBE0/def2‐SVP/D3/COSMO; for details see Computational Methodology.

A schematic reaction mechanism for the C−N bond formation between aniline and bromobenzene mediated by [**Cu(Phen)I**] (denoted as **1**) is depicted in Figure 5. In the initial step, aniline coordinates to the neutral **1** to afford **2** in an exergonic reaction (Δ*G* ^298^= −28 kJ mol^−1^, Δ*E*=−51 kJ mol^−1^). This adduct formation is monotonously downhill and hence diffusion controlled. For such processes, typical free energy barriers are about 20 kJ mol^−1^ – a value we assumed for **TS_1‐2_**.^[29]^ The optimized structure of **2** shows π‐π stacking of the phenyl ring of the aniline and the phenanthroyl moiety, at a distance of 3.5 Å (Figure S47). In the next step, deprotonation of coordinated aniline via **TS_2‐3_** (Figure S48) occurs, for which we estimated a free energy barrier of approximately 25 kJ mol^−1^ (see Computational Methodology for details). As a result, the negatively charged intermediate **3** is formed, with an additional stability of Δ*G* ^298^=−120 kJ mol^−1^ and Δ*E*=−147 kJ mol^−1^. The aniline phenyl ring moves closer to the phenanthroyl fragment at 3.3 Å, while the rest of the structure remains largely unchanged (Figure S49). For the subsequent dissociation of iodide, we estimate a free energy barrier for **TS_3‐4_** of 14 kJ/mol (compare Figure S50; see Computational Methodology for details) to yield intermediate **4** with Δ*G* ^298^=−110 kJ mol^−1^ and Δ*E*=−116 kJ mol^−1^. Here, Cu regains a trigonal planar geometry (Figure S51). Alternatively, **4** can be accessed by iodide dissociation from **2**, resulting in **2 a** (Figure S52), which would be followed by coordination of aniline to give **3 a** (Figure S53), and finally deprotonation. This alternative pathway can be ruled out based on the highly unfavorable energetic profile, which results in relative free energies of Δ*G* ^298^=274 and 160 kJ mol^−1^ for **2 a** and **3 a**, respectively. Approach of the phenyl bromide to **4** proceeds via **TS_4‐5_**. Once again, this step presents a monotonous decrease in electronic energy. We assumed diffusion control and – in the absence of the calixarene cavity – estimated a barrier of Δ*G* ^≠^ ≈20 kJ mol^−1^ (**TS_4‐5_**).^[28]^ The formation is facilitated by π‐π stacking interactions between PhBr and the phenanthroyl moiety, situated at 3.3 Å.

**Figure 6:**
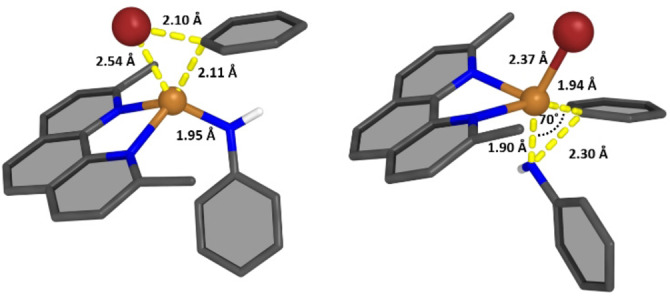
Left: Structure of the transition state for the oxidative addition **TS_5‐6_**, Right: Transition state for the reductive elimination **TS_6‐7_**. In both cases, important structural parameters are given. The two structures were optimized with PBE0/def2‐SVP/D3 in implicit toluene.

Structure **5** (Figure S54) represents the encounter complex from which the Br−C(Ph) oxidative addition step begins. **5** is stabilized by newly formed non‐covalent interactions, with Δ*G* ^298^=−128 kJ mol^−1^ and Δ*E*=−152 kJ mol^−1^. The oxidative addition to form **6** resulted in a free energy barrier for **TS_5‐6_** of Δ*G* ^≠^= 77 kJ mol^−1^. The structure of **TS_5‐6_** in Figure 6 (left) suggests concerted bond cleavage along with Cu−C(Ph) and Cu−Br bond formation. The Cu(III) intermediate **6** (Δ*G* ^298^=−70 kJ mol^−1^ and Δ*E*=−100 kJ mol^−1^, Figure S55) then undergoes C−N coupling by reductive elimination to yield the observed product, associated with a low free energy barrier calculated at Δ*G* ^≠^=12 kJ mol^−1^. The transition state **TS_6‐7_** *(*Δ*G* ^298^=−58 kJ mol^−1^ and Δ*E*=−89 kJ mol^−1^), depicted in Figure 6 (right), has the two aromatic rings of the products at a 70 °C −Cu−N angle as they approach, while increasing the distance to the metal center. Product formation is energetically favored, resulting in a reaction free energy of Δ*G* ^298^=−327 kJ mol^−1^ (Δ*E*=−360 kJ mol^−1^) for the catalyst‐product complex **7** (Figure S56). The final structure, **8**, represents the infinitely separated catalyst and product *(*Δ*G* ^298^=−286 kJ mol^−1^ and Δ*E*=−296 kJ mol^−1^). Note that the extraordinary high stability of **7** compared to **8**, is a result of π‐π stacking interactions of the phenanthroline and one phenyl ring of the aryl amine, as well as a stabilizing intermolecular Br−H interaction. For the subsequent second catalytic cycle, the initial structure is the bromo‐equivalent of **1**. As no significant changes in the reaction profile were observed (see Figure S57), we refrain from further discussion here.

The calculated rate determining step or state, according to Kozuch and Shaik's energy span model,^[30]^ is the difference between the intermediate and the transition state that maximize the energy span. In our case, it is intermediate **5** and the transition state **TS_5‐6_**. For this oxidative addition step, the free energy barrier was calculated at Δ*G* ^≠^=77 kJ mol^−1^. This moderate barrier suggests that the reaction should readily take place at experimental reaction conditions starting with monomeric [**Cu(Phen)I**]. Apart from dimerization as a deactivation pathway, strong interactions between [**Cu(Phen)Br**] and the product may also result in loss of activity or complete deactivation of the catalyst.

Although other mechanisms, such as single electron transfer (SET) or atom transfer are discussed in the literature,^[15]^ they all involve open‐shell Cu(II) and radical species that can be ruled out here. No experimental evidence of such species was found. Hence, an oxidative addition/reductive elimination mechanism is the only viable reaction pathway that agrees with all experimental data. A recent computational study on a similar [**Cu(Phen)I**] catalyst predicted the reaction barriers of all alternative mechanisms to be higher.^[16d]^

### Effect of Confinement

To estimate the effect of confinement on the reaction mechanism, we investigated the supramolecular catalysts and its intermediates by developing a multiscale approach workflow (Figure S58). Due to the flexibility of macrocyclic ligands, enhanced sampling methods were necessary to capture their conformational diversity. In addition, simulation in the full condensed phase, that is, in explicit solvation, was required to retain the cavity. Therefore, we performed accelerated Molecular Dynamics (MD) simulations of the ligand **C_8_PhenMe_6_** in chloroform to explore the potential energy surface. Although the experiments were conducted in toluene, deviations were found to be small (see Figure S59) and computational costs were significantly lower. To also investigate the corresponding Cu(I) complex and selected reaction intermediates as well as transition states, force field parameters had to be generated prior to simulation. After the simulation runs, obtained trajectories were clustered to yield a few representative and structurally diverse conformers. The initial structures thus generated were re‐optimized with DFT (see Computational Methodology for details).

Overall, investigation of the supramolecular system as a whole revealed a similar energetic profile as [**Cu(Phen)I**], as shown in Figure 5 (bottom). For structures **2_calix_**, **4_calix,_ 5_calix_** and **8_calix_** the relative stabilities differ by less than 15 kJ mol^−1^. Notable differences are found for **3_calix_**, **6_calix_**, and **7_calix_**. While the deviation of 54 kJ mol^−1^ between the small model and the supramolecular structure of **3_calix_** arises potentially due to insufficient sampling, the higher relative energy of **6_calix_** can be attributed to the very bulky nature of the intermediate, which occupies the calixarene cavity in its entirety, forcing the cage into unfavorable conformations. In addition to these intermediates, we also converged transition‐states for the oxidative addition (**TS_calix‐5‐6_**) and reductive elimination (**TS_calix‐6‐7_**) steps, respectively. Although the predicted barriers are somewhat higher than in the molecular case, this should not be overemphasized because we did not run MD simulations on these structures. Consequently, we were unable to predict the lowest conformation properly and the reported numbers can be considered as upper bounds to the true TS barriers. Moving forward in the reaction, the release in energy from **6_calix_** towards **7_calix_**, at −255 kJ mol^−1^ is similar to that of the transition from **6** to **7**, at −239 kJ mol^−1^. A structural difference that can be remarked between **7** and **7_calix_** is that the hydrogen bond between the amine and the bromine is no longer present. Instead, it has been replaced by a hydrogen bond to a calixarene oxygen atom, thus weakening the product‐catalyst interaction. Consequently, the release of product from the calixarene cavity is facilitated, with **8_calix_** being only 18 kJ mol^−1^ higher in energy than the previous intermediate, compared to 60 kJ mol^−1^ for **8**. As these are electronic energies only, thermal and entropic corrections will make this step exergonic.

**Figure 7:**
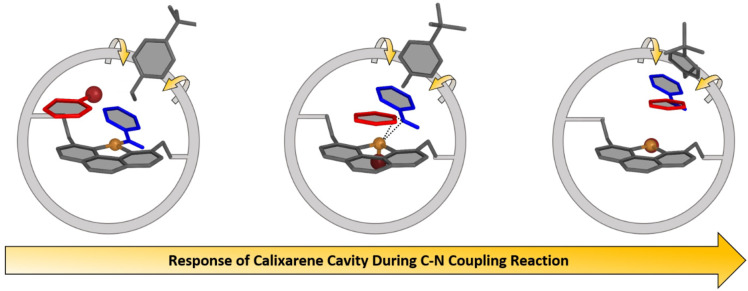
Schematic representation of the calixarene response during the C−N coupling reaction. The calixarene is depicted as a grey circle, surrounding the catalytic center, while one methoxyphenyl unit is shown in detail to highlight the rotation. As PhBr (red) enters the cavity (left), rotation of the methoxyphenyl unit begins, indicated by yellow arrows. C−N coupling starts taking place once PhBr approaches the reaction center (middle), along with continued rotation of the methoxyphenyl. Upon completion of the reaction (right), the product exits the cavity and the methoxyphenyl unit points in the opposite direction.

Analysis of the dynamics of the full system revealed a response of the methoxyphenyl units surrounding the catalytic center upon C−N coupling, depicted in Figure 7. Close inspection of the structures of the optimized supramolecular variants, **1_calix_** through **7_calix_** (Figure S60) reveals that the methoxyphenyl units rotate to adapt the cavity around the reaction center. The rotation of the methoxyphenyl units makes room for the formation of the C−N coupling product, as depicted in Figure 7. To quantify differences in the calixarene flexibility, given by the ability of each methoxyphenyl moiety to rotate, we estimated the dihedral entropy of the species as the reaction progresses from **1_calix_** to **6_calix_**, as shown in Figure S61. Clearly, a rigidification of the ring structure is observed along the reaction coordinate. The fivefold coordinated copper center in **6** and the bulky nature of the aniline and phenyl ligands are responsible for this effect. The large volume taken up by structures **5** and **6** also suggests that the binding position of the phenantroyl bridge is vital in achieving a working catalyst. In this context, the 1,5‐substitution pattern is likely crucial for the C−N coupling steps, since this positioning allows for enough space for the bromoarene substrate to approach the Cu(I) center, while potentially rejecting larger substrates and thereby offering size selectivity. Thus, the cavity plays a critical role in limiting the size of the substrates approaching the reaction center. In addition, it prevents unwanted side reaction, such as dimerization, as found for the molecular analogue **1**. Furthermore, it potentially facilitates the reaction by activating intermediate **6_calix_** and by preventing a strong catalyst‐product interaction (see **7** and **8** vs. **7_calix_** and **8_calix_** in Figure 5).

## Conclusions

In summary, we demonstrated how a macrocyclic encapsulation with a **C_8_** based cavitand enhances C−N coupling activity of [**Cu(C_8_PhenMe_6_)I**] when compared to its molecular analogue [**Cu(Phen)I**], and determined the origin of this increase in catalytic activity. The supramolecular catalyst achieves C−N coupling reactions in toluene with low loadings featuring Earth‐abundant copper. The system offers a chemical environment that is inert to basic conditions, while also favoring interaction of the metal center with the substrates. Based on the experimental and theoretical results, the catalytic process is kinetically limited by the oxidative addition of the haloarene, and by the steric restrictions imposed by the macrocycle suggesting that a 1,5‐phenanthroline bridge is vital for catalysis. Computational studies further revealed that prevention of catalyst aggregation, activation of intermediates as well as facilitation of product release fuels C−N coupling activity in comparison to the molecular analogue. [**Cu(C_8_PhenMe_6_)I**] exhibits better catalytic performance with small amines, in combination with bromoarene derivatives that fit within the cavity with more ease than their iodoarene counterparts. This supramolecular system evidences the advantages of the encapsulation of transition metals, such as higher activity and size selectivity. The detailed mechanistic insights should allow rational modulation of the reactivity by tailoring the properties of the calixarene, or other flexible macrocyclic scaffolds. The presented strategy may be extended to related homogeneous catalytic processes that are currently under investigation in our laboratory.

## Experimental

Unless otherwise specified, the reagents were purchased from commercial suppliers and used without further treatment. Solvents were dried and degasified using standard techniques. Deuterated solvents were degassed prior to use. Catalytic evaluations were performed in oven dried Schlenk flasks under vacuum/N_2_ (purity >99.998 %, Praxair). Melting points were determined with an Electrothermal Mel‐Temp apparatus and are uncorrected. ^1^H and ^13^C NMR spectra were recorded at 300 and 75 MHz with a JEOL Eclipse or Varian Inova 500 MHz spectrometer, the chemical shifts are reported relative to the residual solvent peaks. DOSY experiments were performed using Oneshot DOSY pulse sequence;^[20]^ a duration of 1 ms diffusion‐encoding gradient pulse were used in 0.1 s steps. 65,536 data points were acquired with 256 transients each. IR spectra were collected as KBr pellets on a Bruker Tensor 27 spectrometer from 4000 to 400 cm^−1^. Elemental analyses were performed with a Vario‐Micro V2.0.11 elemental analyzer. FAB^+^ ion mass spectra were obtained on JEOL SX‐102 A spectrometer operated at an accelerating voltage of 10 kV with *m*‐nitrobenzyl alcohol as a solvent (matrix). DART MS were recorded on a JEOL JMS−T100LC spectrometer.

**Synthesis of C_8_PhenMe_6_**. Precursor **C_8_Phen** (500 mg, 0.33 mol) and excess NaH (52 mg, 2.17 mmol) were suspended in anhydrous THF (10 mL) under dinitrogen, followed by addition of MeI (312 mg, 2.17 mmol), the mixture was held at 25 °C for 48 h to achieve complete methylation of the phenolic positions; the formation of a white suspension was noticed. Volatiles were evaporated under reduced pressure, and the crude solid was extracted with 50 mL mixture of dichloromethane/brine (1 : 1). The organic phase was dried over NaSO_4_, and after slow solvent evaporation a colorless solid was obtained. The product was purified by column chromatography on silica gel with dichloromethane as eluant, and washed with hexanes, resulting in a colorless solid in 80 % yield (418 mg, 0.26 mmol). M.p. >210 °C. *δ*_H_ (300 MHz, C_2_D_2_Cl_4_, 293.15 K) 8.06 (m, 4H, Ar_Phen(ac)_), 7.55 (s, 2H, Ar_Phen(b)_), 7.07 (m, 4H, Ar_C8(c)_), 6.94 (m, 4H, Ar_C8(a)_), 6.75 (m, 4H, Ar_C8(d)_), 6.65 (m, 4H, Ar_C8(d)_), 4.34 (d, *J*=16.5 Hz, 4H, CH_2C8(c)_), 4.27 (s, 4H, CH_2Phen_), 3.98 (d, *J*=15.9 Hz, 4H, CH_2C8(a)_), 3.59 (d, *J*=16.1 Hz, 4H, CH_2C8(b)_), 3.49 (d, *J*=15.7 Hz, 4H, CH_2C8(d)_), 3.30 (s, 12 H, MeO_ext_), 2.47 (s, 6H, MeO_ext(a)_), 2.25 (s, 6H, MeO_ext(b)_), 1.23 (s, 18H, *t‐*Bu_ext(b)_), 1.14 (s, 18H, *t‐*Bu_ext(a)_), 0.90 (s, 36H, *t‐*Bu_int_). ν_max_ (KBr) 2955 (CH_3_ and CH_2_), 2866 (CH_3_ and CH_2_) and 1479 (Ar). *m/*z (FAB^+^) 1587 [**C_8_PhenMe_6_H**]^+^. Elemental analysis for C_108_H_134_N_2_O_8_ ⋅ H_2_O ⋅ CHCl_3,_ found: C, 75.87; H, 8.00; N, 1.62; requires: C, 75.88, H, 7.86, N, 1.43.

**Synthesis of [Cu(C_8_PhenMe_6_)I]**. The cavitand **C_8_PhenMe_6_** (30 mg, 0.018 mmol) and CuI (3.5 mg, 0.018 mmol) were placed in a 50 mL Schlenk tube with 2 mL of toluene under dinitrogen with constant stirring. Volatiles were evaporated after 2 h of stirring under reduced pressure, resulting in an orange crystalline solid in 92 % yield (30 mg, 0.016 mmol), m.p. >230 °C. *δ*_H_ (300 MHz, toluene‐d_8_, 393.15 K) 8.34 (d, *J*=8.5 Hz, 1 H, Ar_Phen_), 8.18 (d, 1 H, Ar_Phen_), 7.68 (m, 1 H, Ar_Phen_), 7.62 (m, 1 H, Ar_Phen_), 7.16 (m, 8 H, Ar_C8_), 7.09 (m, 3 H, Ar_toluene_), 7.00 (m, 8 H, Ar_toluene_), 6.98 (m, 2 H, Ar_toluene_), 5.25 (m, 2 H, CH_2Phen_), 4.87 (d, *J*=16 Hz, 2 H, CH_2exo_), 4.73 (m, 2 H, CH_2exo_), 4.52 (m, 5 H, CH_2exo_ y CH_2Phen_), 4.21 (d, *J*=16 Hz, 4 H, CH_2exo_), 3.75 (m, 4H, CH_2exo_), 4.18 (m, 4 H, CH_2endo_), 3.81 (m, 4 H, CH_2endo_), 3.59 (s, 9 H, CH_3int_), 3.13 (s, 9 H, CH_3ext_), 2.09 (s, 3 H, CH_3toluene_), 1.30 (s, 36 H, *t‐*Bu_ext_), 1.18 (m, 36 H, *t‐*Bu_int_). *m/*z (FAB^+^) 1649 [**Cu(C_8_PhenMe_6_)**]^+^.

**General procedure for coupling reactions**. Typically, 4 mg (0.02 mmol, 2.5 % mol) of CuI and 30 mg (0.02 mmol, 2.5 % mol) of **C_8_PhenMe_6_** in 2.0 mL of toluene were stirred in a 25 mL Schlenk flask for 30 min under dinitrogen. Then, 73 μL of aniline (74 mg, 0.80 mmol), an equimolar amount of the aryl bromide, and KO*t*Bu (150 mg, 1.2 mmol) were added, and the temperature was raised to 110 °C for 36 h. After this time, the products were isolated by flash column chromatography on basic alumina as stationary phase; the mobile phase employed consisted of a hexanes/dichloromethane gradient, starting with 0 and up to 20–30 % dichloromethane, depending on the polarity of the product obtained. Their identity was confirmed by comparison with literature spectroscopic data (see Figures S20‐S45 in the SI).[^24b^, ^56^] The same reaction conditions were employed for comparative studies with **C_8_Phen**, and the molecular analogues [**Cu(Phen(OAr)_2_)I**] and [**Cu(Phen)I**]_**2**_, except that the catalyst loadings corresponded to 10 % mol for the latter two complexes.

### Computational Methodology

A multiscale modelling protocol was designed to describe the supramolecular catalyst [**Cu(C_8_PhenMe_6_)I**] (see Figure S58 for a schematic overview). We first studied the C−N coupling mechanism with monomeric [**Cu(Phen)I**] (**1**) as the catalyst, before assessing the flexibility of the macrocyclic ligand and studying the supramolecular system.

**Quantum chemical studies of [Cu(Phen)I]**. The initial geometry for **1** was constructed using Gaussview 6.0.16.^[31a]^ Unless otherwise noted, quantum chemical calculations were performed with Turbomole 7.3.[^31b^, ^31c^] All structures were roughly preoptimized in *C*1 symmetry using the BP86^[32]^ functional and def2‐SVP^[33]^ basis set with empirical dispersion corrections of the D3 type,^[34]^ as well as implicit solvent corrections using the Conductor‐Like Screening Model (COSMO)^[35]^ as implemented in Turbomole. A dielectric constant of ϵ=2.4 was chosen to model toluene. The structures were further optimized with the PBE0 functional^[36]^ which is known to provide good results when investigating structures involving transition metals[^37^, ^38^] and the same basis set, solvation model and dispersion corrections as described above. Single point energy calculations were performed on the PBE0 optimized structures with the def2‐TZVP basis set^[33]^ using the resolution‐of‐identity technique for speed‐up^[39]^ and D3 corrections. Again, calculations were performed in toluene modelled as implicit solvent, to obtain more accurate electronic energy values. Harmonic frequency calculations were performed on the optimized structures to verify that they are indeed energy minima. Reported energies are either relative electronic energies Δ*E* (PBE0/def2‐TZVP/D3/COSMO//PBE0/def2‐SVP/D3/‐COSMO) or relative free energies Δ*G*[^40^, ^41^] (see SI for details on free energy calculations) at 298.15 K, all given in kJ mol^−1^.

The transition states (TS) were obtained by using the eigenvector following method implemented in Turbomole.[^31b^, ^31c^] A successful transition state was confirmed by the presence of exactly one imaginary frequency matching the reaction coordinate.

In some cases, transition states could not be localized by the eigenvector following method. In such cases, tailored strategies were used to approximate the TSs. For the association reactions *via* **TS_1‐2_** and **TS_4‐5_**, the energy was found to monotonously decrease when the ligand approached the catalysts, which indicates a diffusion‐controlled transition state. To verify this, the structures of the so obtained intermediates were modified in such a way that the two molecules are 6 Å apart and then left to freely optimize. The optimizations completed reaching the structure of the intermediate, providing further proof of a diffusion‐controlled transition state. For these cases, as a suitable estimate for the transition state free energy, 20 kJ mol^−1^ was taken as suggested in literature.^[29]^ Transition states for dissociation reactions could not be localized with eigenvector following methods either, because dissociations were found to be energetically uphill in the electronic energy. However, as the reactants separate, they obtain additional entropy. To approximate the transition state energies, a method put forward by Baik was applied^[42]^ accounting for the onset of entropy upon dissociation, which has been successful for Au catalysts in the past^[43]^ and proved useful in estimating **TS_4‐5_** (Figure S44), see SI for more details. Particularly challenging was the hydrogen abstraction (**TS_2‐3_**). As the deprotonation in experiment is facilitated by a base, we approximated the barrier for hydrogen abstraction by modelling the reaction path of hydrogen transfer from **2** to the base (see Figure S48 for structure). It is noteworthy here that all approximated reaction barriers are significantly lower than the rate determining state **TS_5‐6_**, for which a transition state was localized with eigenvector following.

**Molecular Dynamics simulations to assess conformational flexibility**. To obtain a representative structural ensemble of the macrocyclic ligand **C_8_PhenMe_6_** for subsequent DFT studies, the conformational spaces was explored in the absence and in the presence of Cu(I), using classical accelerated Molecular Dynamics (aMD) simulation – one approach shown to yield reliable conformational ensembles for macrocycles.^[17]^ A sampling in explicit solvent proved to be essential to retain intact cavities, as initial sampling attempts in implicit solvent resulted in their collapse. In the absence of Cu(I), the initial structure of **C_8_PhenMe_6_** was adapted from the crystal structure of a calix[8]arene/Cs^[14]^ and appropriate structural modification. Force field parameters for **C_8_PhenMe_6_** were generated using Amber's Generalized Force Field (GAFF),^[44]^ partial charges were obtained with RESP fitting. MD simulations were carried in chloroform as well as benzene (as a model for toluene used in experiment). As results were largely similar (see Figure S59), we restricted ourselves to chloroform only to reduce computational costs.

To obtain adequate sampling of the catalyst, we performed twelve 1μs aMD simulations, followed by a hierarchical clustering to obtain a structurally diverse ensemble (see details SI for more details on these calculations performed with Amber).[^45^, ^46^, ^47^, ^48^, ^49^, ^50^, ^51^]

In addition, we simulated the assembled supramolecular structures **1_calix_** and **6**_**calix**_, to assess how the flexibility of the cavity changes upon Cu coordination. Therefore, a parametrization of the metal ion at the center of the molecule was required. MCPB.py^[52]^ was selected as the tool of choice for the parametrization step. The initial structure was created in Gaussview by taking a cluster representative from the simulation without metal, after which the respective QM optimized structure was fitted in (see Assembly of supramolecular catalyst for DFT investigations). The resulting structures were then optimized at the PBE0/def2‐SVP/D3/COSMO level. The next step involved separating the metal center and ligands into individual substructures and following the MCPB.py procedure. The structure optimizations and frequency calculations were performed at the PBE0/def2‐SVP/D3 level. Using the resulting parameters, equilibration, generation of boosting parameters and production runs in explicit chloroform were performed according to the protocol outlined above.

To investigate the flexibility of the calixarene units, a dihedral analysis was used, to characterize the rotations between each calixarene unit. Dihedral angles were assigned between the planes of each of the six‐membered rings of the calixarene units, as changes in these angles accurately describe the movements within the calixarene ring. Using the X‐entropy script^[53]^ to perform an entropic analysis on the angle distributions, the relative entropy values were obtained, thus, leading to an accurate estimation of the flexibility.

**Assembly of supramolecular catalyst for DFT investigations**. Once the [**Cu(Phen)I**] (**1**) structure pathway investigation was completed, the next step was to study the effects of the confinement by the calix[8]arene on the energetic landscape. To this end, a fitting of the reaction intermediates back into the **C_8_** ring was necessary. Cluster representatives of the molecular dynamics simulations were selected using a hierarchical clustering algorithm. The cluster representative with the largest population of the aMD simulation of structure **1_calix_** was chosen as the starting point for a fitting of all QM optimized structures. The fitting process was automated using a python script via PyMOL,^[54]^ which aligned the phenanthroyl backbones of the large and small models. The calixarene ring, along with the backbone from the large model were kept and combined with the remaining atoms of the small model. The results of the fitting were then pre‐optimized with GFN2‐xTB,^[55]^ followed by an optimization with PBE0/def2‐SV(P)/D3/COSMO. Final single point energies were again calculated with PBE0/def2‐TZVP/D3/COSMO.

Structural parameter measurements and visualizations were done with PyMOL.^[54]^

## Supporting Information

Detailed characterization of cavitand **C_8_PhenMe_6_** and the supramolecular system [**Cu(C_8_PhenMe_6_)I**], characterization of coupling products, details concerning the theoretical investigations.

## Conflict of interest

The authors declare no competing financial interest.

1

## Supporting information

As a service to our authors and readers, this journal provides supporting information supplied by the authors. Such materials are peer reviewed and may be re‐organized for online delivery, but are not copy‐edited or typeset. Technical support issues arising from supporting information (other than missing files) should be addressed to the authors.

Supporting Information

## Data Availability

Experimental characterization of the **C_8_PhenMe_6_** and the supramolecular system [**Cu(C_8_PhenMe_6_)I**] as well as all coupling products can be found in the Supporting Information. Coordinates of all investigated structures are supplied in an xyz file with the DOI: 10.5281/zenodo.7008048 that can be downloaded at https://zenodo.org/.
