# Supplementary material for: Encapsulation Enhances the Catalytic Activity of C‐N Coupling: Reaction Mechanism of a Cu(I)/Calix[8]arene Supramolecular Catalyst
Source: ChemCatChem. 2022 Sep 1;14(20):e202200662. doi: 10.1002/cctc.202200662 (PMC9804476; doi:10.1002/cctc.202200662)
Supplement: Supplementary file 1 — Supporting Information [file CCTC-14-0-s001.pdf]

ChemCatChem

Supporting Information

Encapsulation Enhances the Catalytic Activity of C-N Coupling: Reaction Mechanism of a Cu(I)/Calix[8]arene Supramolecular Catalyst

Radu A. Talmazan[+], J. Refugio Monroy[+], Federico del Río-Portilla, Ivan Castillo,* and Maren Podewitz*

Table of Contents

S.1. General experimental procedures 1
S.2. Synthetic procedures 2
S.3. Characterization 7
S.4. General procedure for C-N cross-coupling reactions and characterization of the coupling products 19
S5. Computational Investigations 34
S6. References 50

S.1. General experimental procedures

Unless otherwise specified, the reagents were purchased from commercial suppliers and used without further treatment. Solvents were dried and degassed using standard techniques.[1] Deuterated solvents were degassed prior to use by 3 freeze-pump-thaw cycles. Catalysis evaluations were performed in oven-dried Schlenk flasks under vacuum/N_2 (purity > 99.998%, Praxair). ^{1}H and ^{13}C NMR spectra were recorded at RT with a 300 MHz JEOL Eclipse spectrometer, chemical shifts are reported according to the residual solvent peaks. IR spectra were recorded as KBr pellets on a Bruker Tensor 27 spectrometer from 4000 to 400 cm^{-1}. Elemental analyses were performed with a Vario-Micro V2.0.11 elemental analyser. Positive ion FAB$^+$ mass spectra were obtained on a JEOL SX-102A spectrometer operated at an accelerating voltage of 10 kV in a *m*-nitrobenzyl alcohol matrix. DART MS were recorded on a JEOL JMS-T100LC spectrometer. Melting points were determined with an Electrothermal Mel-Temp apparatus and are uncorrected.

S.2. Synthetic procedures

S.2.1. Synthesis of 2,9-bis(bromomethyl)-1,10-phenanthroline ($PhenBr_2$)

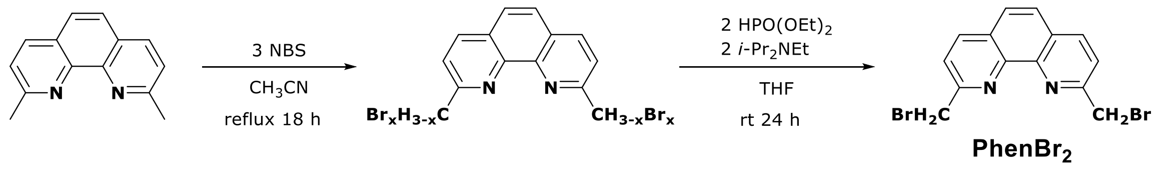

Scheme 1. Synthesis of 2,9-bis(bromomethyl)-1,10-phenanthroline ($PhenBr_2$).

Optimisation of the previously reported protocol[2] was adapted, with the reaction time modified. 2,9-dimethyl-1,10-phenanthroline (0.88 g, 4.17 mmol) was dissolved in 30 mL of acetonitrile in a 250 mL round-bottom Schlenk flask covered with black paper foil. Under constant stirring, recrystallised *N*-bromosuccinimide (NBS 2.23 g, 12.51 mmol) was added to the solution, then the temperature was raised to reflux with an oil bath and was maintained for 18 h. After that, volatiles were evaporated, the crude solid was extracted with diethyl ether, and washed with a saturated solution of $NaHCO_3$ (50 mL). Then, the solid was dissolved in 40 mL of THF, the brown solution was cooled to 0 °C. Thereupon, diethyl phosphite (1.1 mL, 8.34 mmol, 98%) and *N,N*-diisopropylethylamine (1.453 mL, 8.34 mmol, 99%) were added to the solution. After 24 h, volatiles were evaporated and the product was purified by column chromatography on silica gel with dichloromethane as eluant, resulting in an orange crystalline solid in 40% overall yield (0.61 g, 1.68 mmol), m.p. > 110 °C. δ_H (300 MHz, $CDCl_3$, 393.15 K) 8.26 (d, J = 8.34 Hz, 2 H, $Ar_{Phen(c)}$), 7.92 (d, J = 8.33 Hz, 2 H, $Ar_{Phen(a)}$), 7.82 (d, J = 7.80 , 2H, $Ar_{Phen(b)}$), 7.26 (s, 3 H, $CDCl_3$), 4.97 (s, 4 H, CH_{2Phen}). *m/z* (DART-MS) 367 [**$PhenBr_2H$**]$^+$.

S.2.2. Synthesis of 1,5-(2,9-dimethyl-1,10-phenanthroline)-*p*-tert-butylcalix[8]arene (C_8Phen)

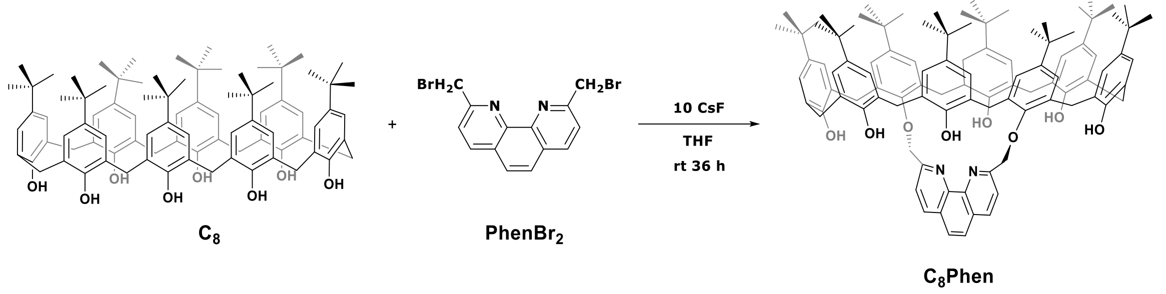

Scheme 2. Synthesis of 1,5-(2,9-dimethyl-1,10-phenanthroline)-*p*-tert-butylcalix[8]arene (**C_8Phen**).

C_8Phen was obtained according to the previously reported protocol,[2] with modifications in the purification procedure. Previously synthesized *p*-tert-butylcalix[8]arene (obtained according to the literature procedure,[3] 0.80 g, 0.62 mmol) and CsF (0.94 g, 6.22 mmol) were dried in a round-bottom Schlenk flask at 120 °C for 2 h. After cooling to room temperature, the solids were dissolved in 15 mL of THF, the formation of a white suspension was observed. The temperature was increased to 50 °C for 12 h while stirring, resulting in a yellow solution. **$PhenBr_2$** (0.28 g, 0.73 mmol) was then added, and the mixture was stirred at room temperature for 36 h. Then, volatiles were evaporated under reduced pressure, and the crude solid was dissolved in 10 mL of chloroform/toluene (10:1). The organic phase was washed with 15 mL of 0.1 M HCl, followed by 50 mL of saturated $NaHCO_3$ solution, and extracted with 20 mL of chloroform. The organic phase was dried over $CaCO_3$, and after slow solvent evaporation an orange solid was obtained. The product was purified by column chromatography on silica gel with dichloromethane as eluant, and the product was washed with hexanes, resulting in a white crystalline solid in 86% yield (0.78 g, 0.52 mmol), m.p. > 230 °C. δ_H (300 MHz, $CDCl_3$, 393.15 K) 9.48 (s, 8 H, OH), 8.39 (d, J = 8.27 Hz, 2 H, $Ar_{Phen(b)}$), 7.78 (m, 2 H, $Ar_{Phen(a)}$), 7.24 (s, 2 H, $Ar_{Phen(b)}$), 7.18 (m, 16 H, Ar_{C8}), 5.26 (s, 4 H, CH_{2Phen}), 4.28 (d, J = 12.9, 8 H, CH_{2exo}), 3.50 (d, J = 12.9, 8 H, CH_{2endo}), 1.25 (m, 48 H, *t*-Bu), 1.17 (m, 24 H, *t*-Bu). *m/z* (FAB^+) 1502 [**C_8PhenH**]$^+$.

S.2.3. Synthesis of 1,5-(2,9-dimethyl-1,10-phenanthroline)-2,3,4,6,7,8-hexamethyl-*p*-*tert*-butylcalix[8]arene (C_8PhenMe$_6$)

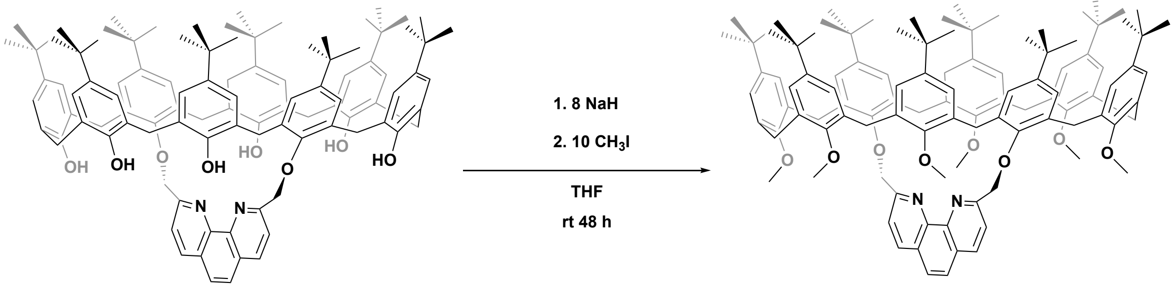

C_8Phen   C$_8$PhenMe$_6$

Scheme 3. Synthesis of 1,5-(2,9-dimethyl-1,10-phenanthroline)-2,3,4,6,7,8-hexamethyl-*p*-*tert*-butylcalix[8]arene (**C$_8$PhenMe$_6$**).

Under inert atmosphere, the previously synthetized **C$_8$Phen** (500 mg, 0.33 mmol) was dissolved in 10 mL of anhydrous THF in a 50 mL round bottom Schlenk flask, NaH (52 mg, 2.17 mmol, 90%) was added, after one hour under constant stirring, the formation of a yellow precipitate was observed. Prior addition of CH_3I (312 mg, 2.17 mmol), the Schlenk flask was covered with black paper foil, then, the mixture was stirred at room temperature for 48 h, the formation of a white suspension was noticed. Volatiles were evaporated under reduced pressure, and the crude solid obtained was extracted with 50 mL mixture of dichloromethane/brine (1:1). The organic phase was dried over Na_2SO_4, and after slow evaporation of the solvent a colourless solid was obtained. The product was purified by column chromatography on silica gel with dichloromethane as eluant, and the product was washed with hexanes, resulting in a white crystalline solid in 80% yield (418 mg, 0.26 mmol). m.p. > 210 °C. δ_H (300 MHz, $C_2D_2Cl_4$, 293.15 K) 8.06 (m, 4H, $Ar_{Phen(ac)}$), 7.55 (s, 2H, $Ar_{Phen(b)}$), 7.07 (m, 4H, $Ar_{C8(c)}$), 6.94 (m, 4H, $Ar_{C8(a)}$), 6.75 (m, 4H, $Ar_{C8(d)}$), 6.65 (m, 4H, $Ar_{C8(d)}$), 4.34 (d, J = 16.5 Hz, 4H, $CH_{2C8(c)}$), 4.27 (s, 4H, CH_{2Phen}), 3.98 (d, J = 15.9 Hz, 4H, $CH_{2C8(a)}$), 3.59 (d, J = 16.1 Hz, 4H, $CH_{2C8(b)}$),), 3.49 (d, J = 15.7 Hz, 4H, $CH_{2C8(d)}$), 3.30 (s, 12 H, MeO_{ext}), 2.47 (s, 6H, $MeO_{ext(a)}$), 2.25 (s, 6H, $MeO_{ext(b)}$), 1.23 (s, 18H, t-$Bu_{ext(b)}$), 1.14 (s, 18H, t-$Bu_{ext(a)}$), 0.90 (s, 36H, t-Bu_{int}). ν ν_{max} (KBr) 2955 (CH_3 and CH_2), 2866 (CH_3 and CH_2) y 1479 (Ar). *m/z* (FAB$^+$) 1587 [**C$_8$PhenMe$_6$H**]$^+$. Elemental analysis for $C_{108}H_{134}N_2O_8 \cdot H_2O \cdot CHCl_3$, found: C, 75.87; H, 8.00; N, 1.62; requires: C, 75.88, H, 7.86, N, 1.43.

S.2.4. Synthesis of 1,5-(2,9-dimethyl-1,10-phenanthroline)-2,3,4,6,7,8-hexamethyl-*p*-tert-butylcalix[8]arene copper(I) complex [Cu(C_8PhenMe$_6$)I]

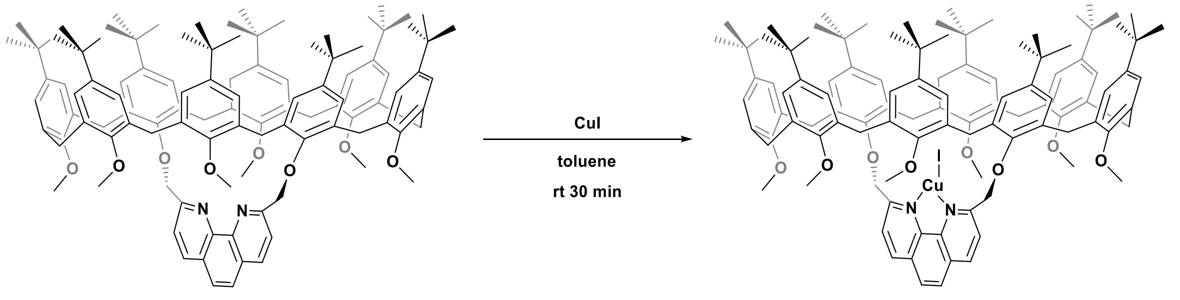

C_8PhenMe$_6$ **Cu(C_8PhenMe$_6$)I**

Scheme 4. Synthesis of 1,5-(2,9-dimethyl-1,10-phenanthroline)-2,3,4,6,7,8-hexamethyl-*p*-tert-butylcalix[8]arene copper(I) complex [**Cu(C_8PhenMe$_6$)I**].

C_8PhenMe$_6$ (30 mg, 0.018 mmol) and CuI (3.5 mg, 0.018 mmol) were placed in a 50 mL Schlenk tube with 2 mL of toluene under constant stirring for 30 min at room temperature under N_2. Volatiles were then evaporated under reduced pressure, resulting in an orange crystalline solid in 92% yield (30 mg, 0.016 mmol), m.p. > 230 °C. δ_H (300 MHz, toluene-d$_8$, 393.15 K) 8.34 (d, *J* = 8.5 Hz, 1 H, Ar_{Phen}), 8.18 (d, 1 H, Ar_{Phen}), 7.68 (m, 1 H, Ar_{Phen}), 7.62 (m, 1 H, Ar_{Phen}), 7.16 (m, 8 H, Ar_{C8}), 7.09 (m, 3 H, $Ar_{toluene}$), 7.00 (m, 8 H, $Ar_{toluene}$), 6.98 (m, 2 H, $Ar_{toluene}$), 5.25 (m, 2 H, CH_{2Phen}), 4.87 (d, *J* = 16 Hz, 2 H, CH_{2exo}), 4.73 (m, 2 H, CH_{2exo}), 4.52 (m, 5 H, CH_{2exo} y CH_{2Phen}), 4.21 (d, *J* = 16 Hz, 4 H, CH_{2exo}), 3.75 (m, 4H, CH_{2exo}), 4.18 (m, 4 H, CH_{2endo}), 3.81 (m, 4 H, CH_{2endo}), 3.59 (s, 9 H, CH_{3int}), 3.13 (s, 9 H, CH_{3ext}), 2.09 (s, 3 H, $CH_{3toluene}$), 1.30 (s, 36 H, *t*-Bu_{ext}), 1.18 (m, 36 H, *t*-Bu_{int}). *m/z* (FAB$^+$) 1649 [**Cu(C_8PhenMe$_6$)**]$^+$.

S.2.5. Synthesis of the bis(2,9-dimethyl-1,10-phenanthroline) copper(I) complex [Cu(Phen)(μ-I)]$_2$

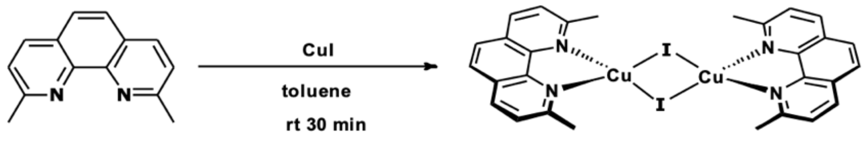

[Cu(Phen)(μ -I)]$_2$

Scheme 5. Synthesis of the bis(2,9-dimethyl-1,10-phenanthroline) copper(**I**) complex [**Cu(Phen)(μ-I)**]$_2$.

2,9-dimethyl-1,10-phenanthroline (32 mg, 0.15 mmol) and CuI (29 mg, 0.15 mmol) were dissolved in 2 mL toluene in a 50 mL Schlenk tube under N_2, the mixture was under constant stirring for 30 min at room temperature. Volatiles were evaporated under reduced pressure, resulting in a red solid in 37% yield (45 mg, 0.06 mmol). The low yield is due to the conditions were imposed to obtain the (1:1) Phen-CuI complex. m.p. > 230 °C. δ_H (300 MHz, toluene-d8, 393.15 K) 8.55 (d, *J* = 7.80 Hz, 2 H, $Ar_{Phen(c)}$), 8.06 (s, 2 H, $Ar_{Phen(a)}$), 7.82 (d, *J* = 7.9 Hz, 2 H, $Ar_{Phen(b)}$), 7.26 (s, 3 H, $CDCl_3$), 2.45 (s, 6 H, CH_{3Phen}). *m/z* (DART-MS) 479 [**Cu(Phen)$_2$**]$^+$.

S.2.6. Synthesis of the 2,9-bis((4-(tert-butyl)phenoxy)methyl)-1,10-phenanthroline copper(I) complex [Cu(Phen(OAr)$_2$)I]

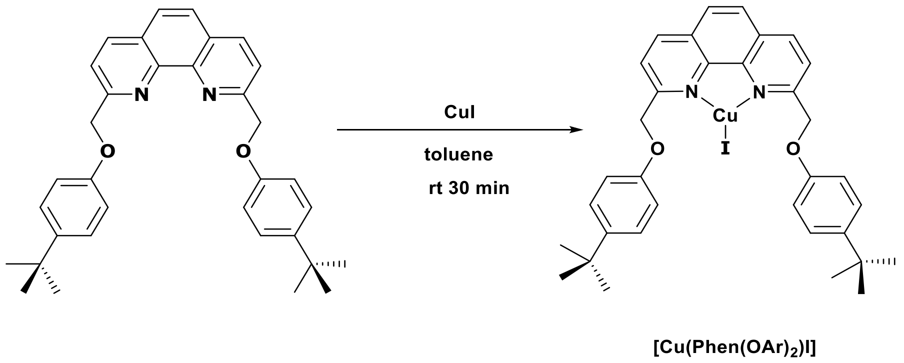

Scheme 6. Synthesis of the 2,9-bis((4-(tert-butyl)phenoxy)methyl)-1,10-phenanthroline copper(I) complex [**Cu(Phen(OAr)$_2$)I**].

2,9-bis((4-(*tert*-butyl)phenoxy)methyl)-1,10-phenanthroline (obtained according to the literature procedure,[4] 60 mg, 0.12 mol) and CuI (23 mg, 0.12 mol) were dissolved in 2 mL toluene in a 50 mL Schlenk tube under N_2, the mixture was stirred for 30 min at room temperature. Volatiles were evaporated under reduced pressure, resulting in a red solid in 93% yield (76 mg, 0.11 mmol), m.p. > 230 °C. δ_H (300 MHz, $CDCl_3$, 298.15 K) 8.31 (d, *J* = 8.3 Hz, 2H), 7.97 (d, *J* = 8.3 Hz, 2H), 7.81 (s, 2H), 7.34 (d, *J* = 8.9 Hz, 4 H), 7.26 (s, 3H, $CDCl_3$), 7.02 (m, 4H), 5.65 (s, 4H), 1.31 (s, 18H). *m/z* (ESI-MS) 567.3 [**Cu(Phen(OAr)$_2$)**]$^+$.

S.3. Characterization

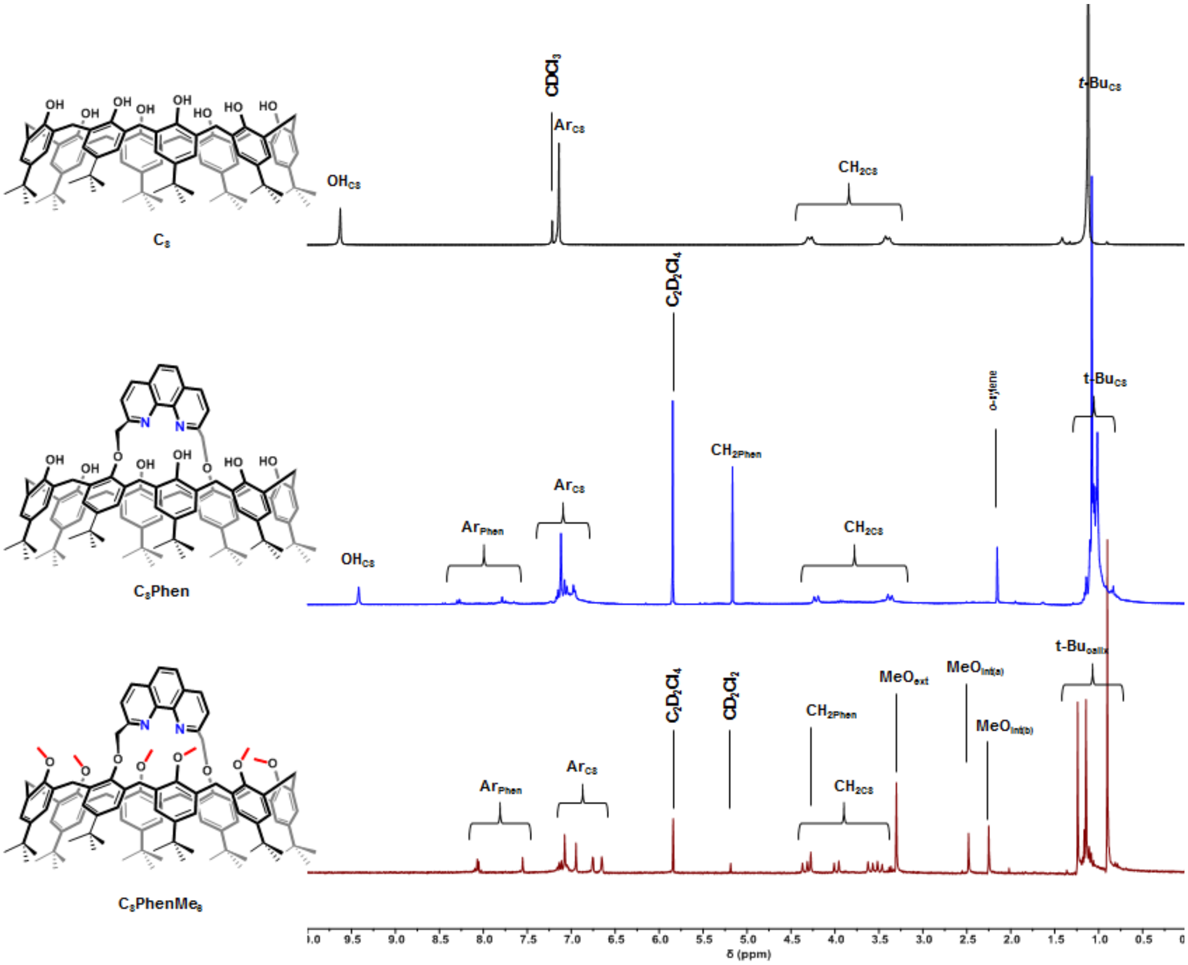

Figure S1. Comparison of the ^{1}H NMR spectra of the macrocycles **C_8** in $CDCl_3$ (black) at 293.15 K, **C_8Phen** in $C_2D_2Cl_4$ (blue) at 293.15 K, and **C_8PhenMe$_6$** in $C_2D_2Cl_4$ (red) at 393.15 K.

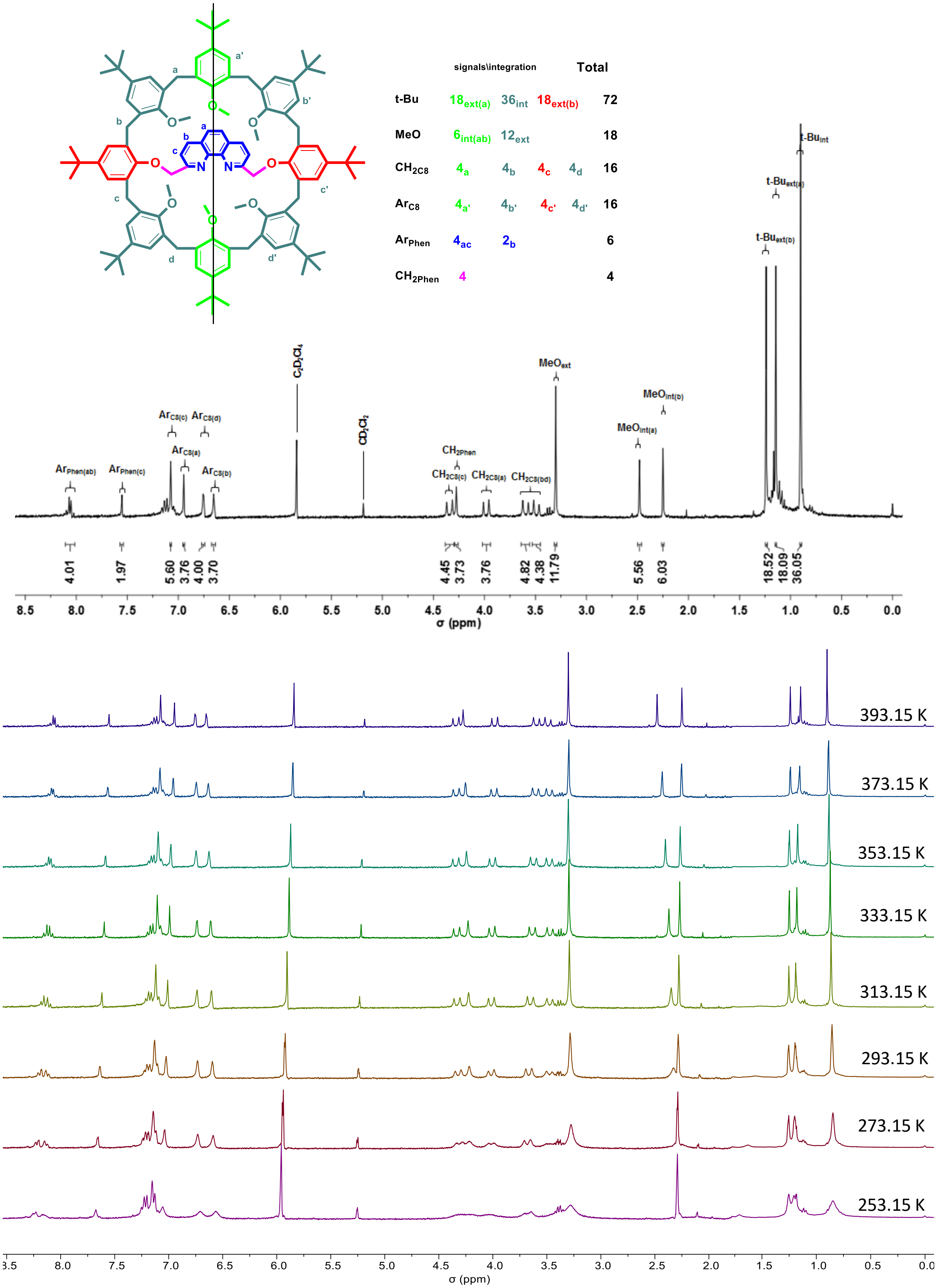

Figure S2. Top: analysis of the ^{1}H NMR spectrum of **C_8PhenMe$_6$** in $C_2D_2Cl_4$ at 393.15 K. Bottom: VT ^{1}H NMR spectra of **C_8PhenMe$_6$** in $C_2D_2Cl_4$ from 253.15 to 393.15 K.

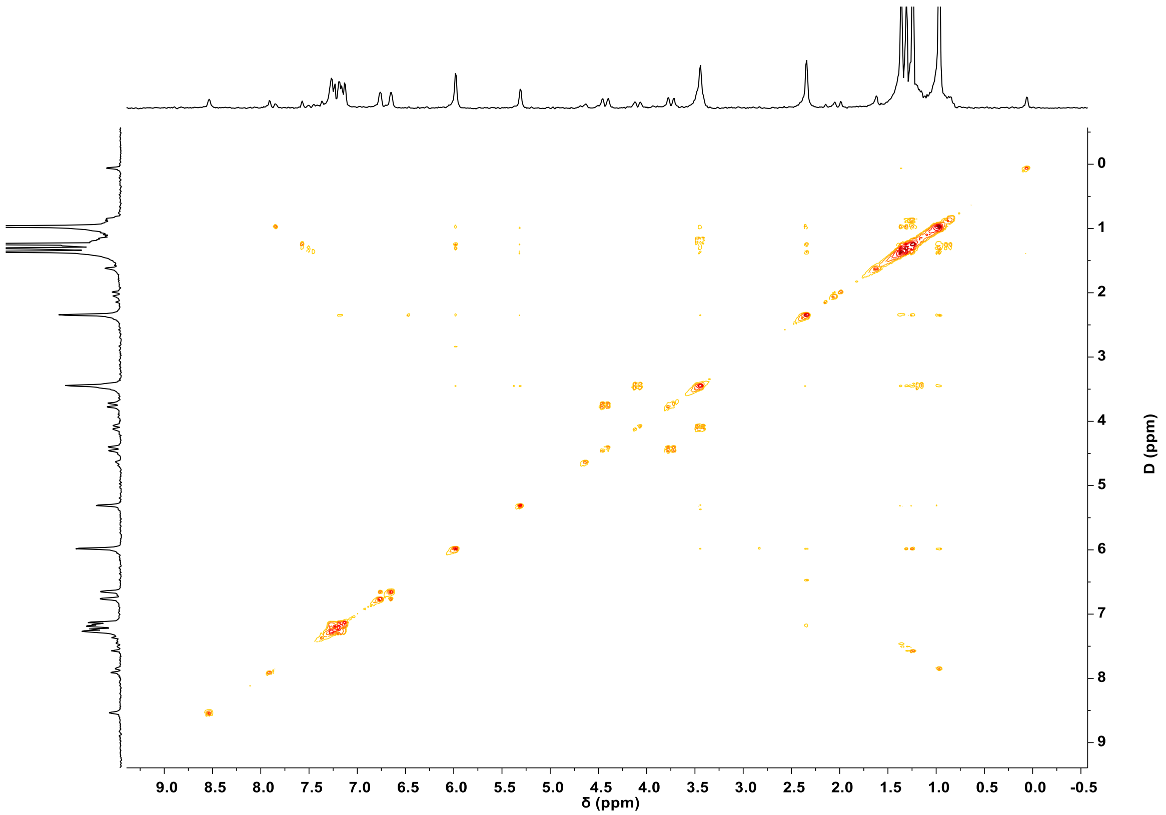

Figure S3. COSY NMR spectrum of **C_8PhenMe$_6$** in $C_2D_2Cl_4$ at 298.15 K.

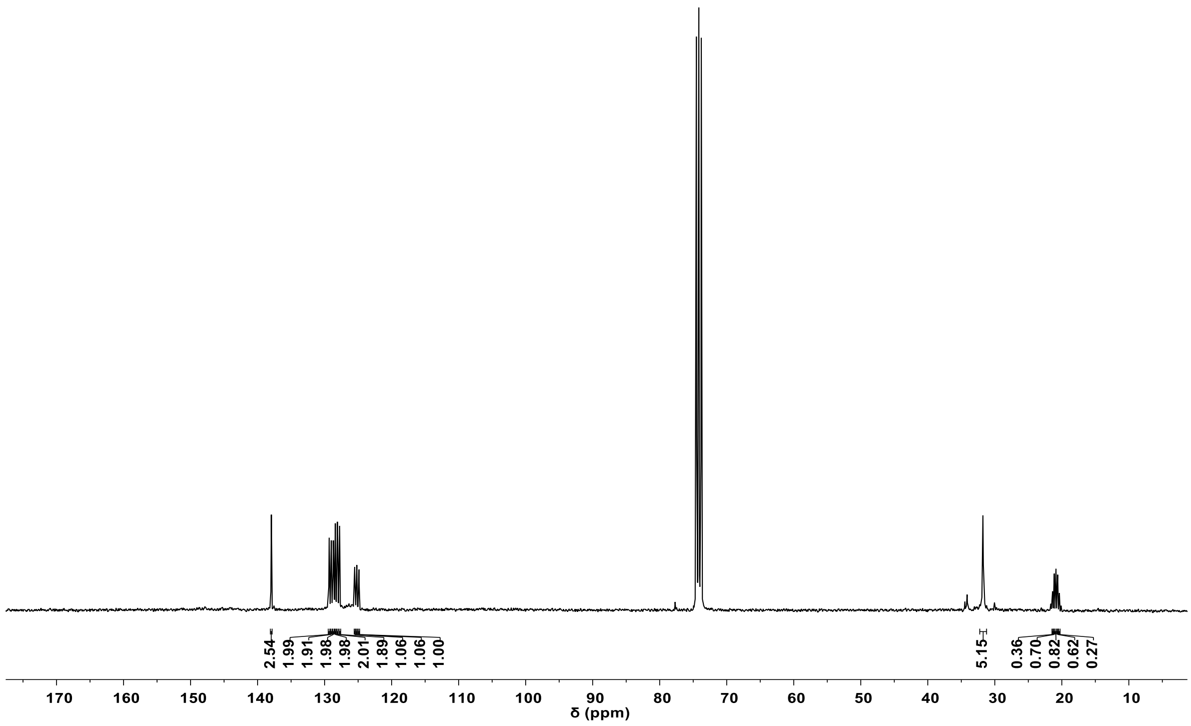

Figure S4. ^{13}C NMR spectrum of **C_8PhenMe$_6$** in $C_2D_2Cl_4$ at 298.15 K.

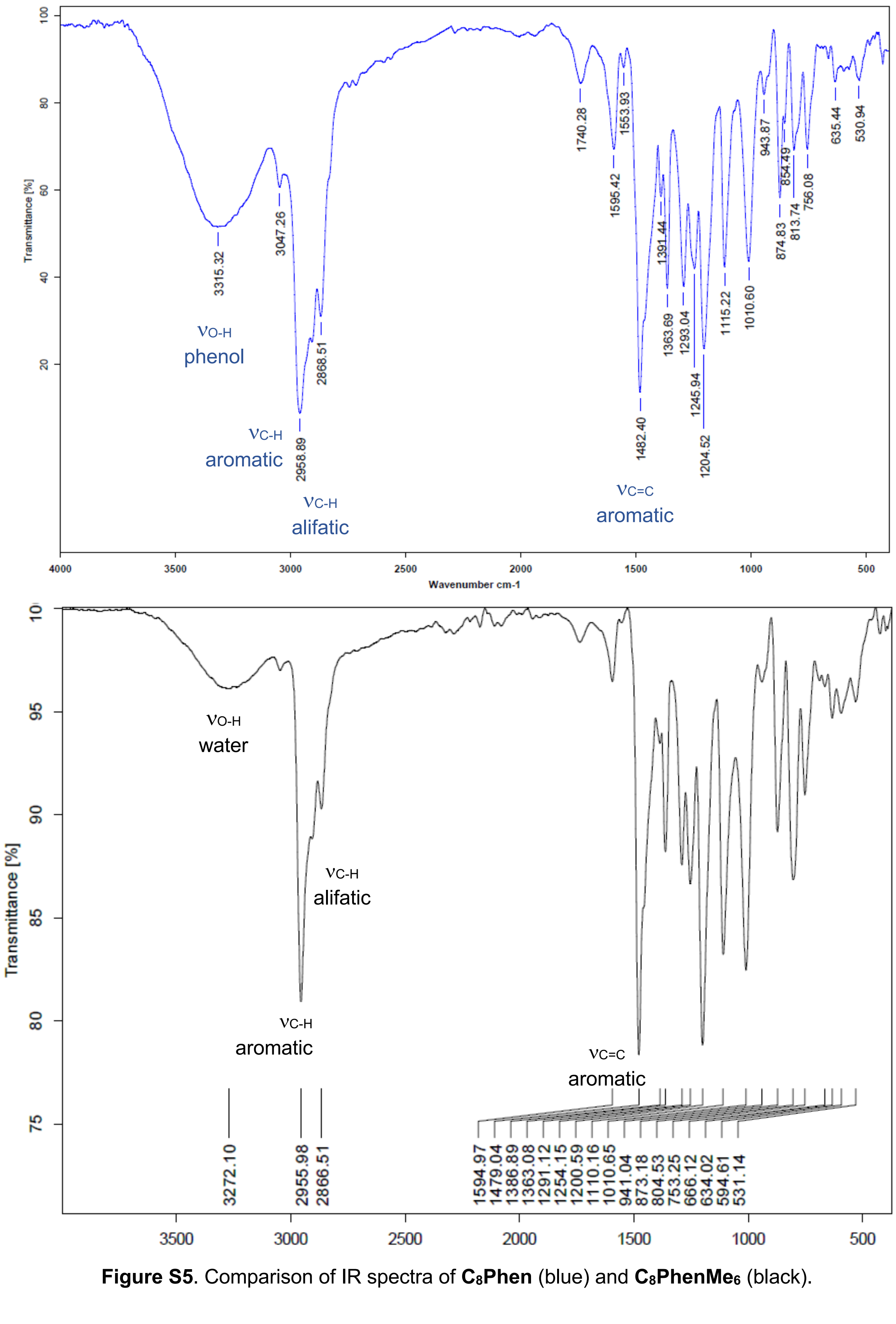

Figure S5. Comparison of IR spectra of **C8Phen** (blue) and **C8PhenMe6** (black).

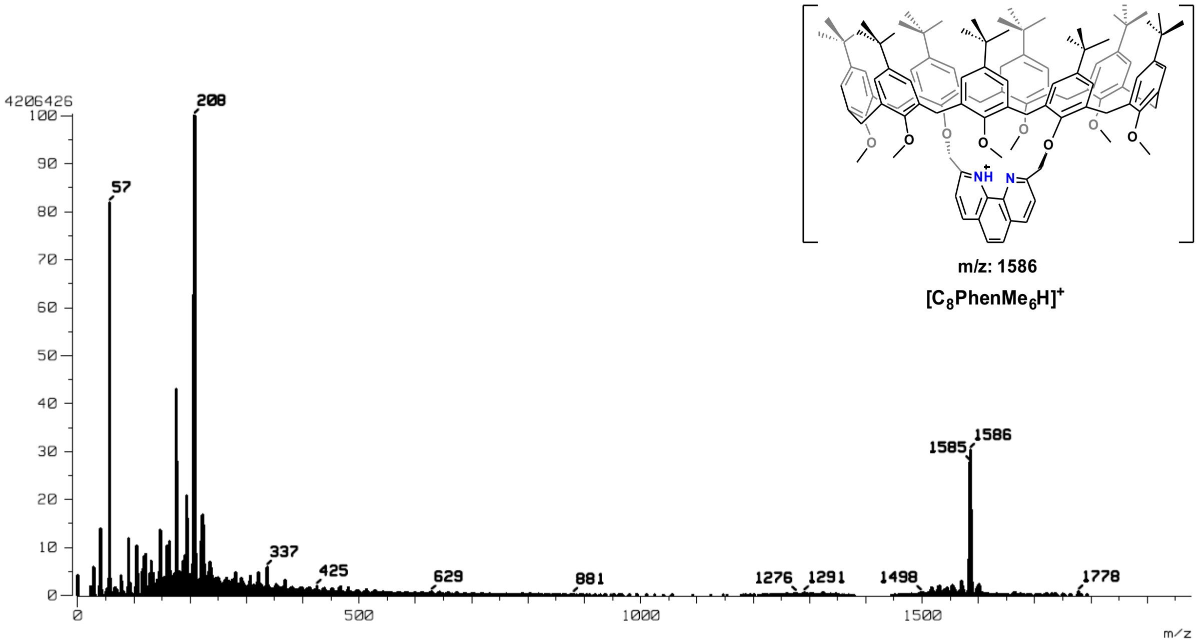

Figure S6. FAB$^+$ MS of **$C_8PhenMe_6$**.

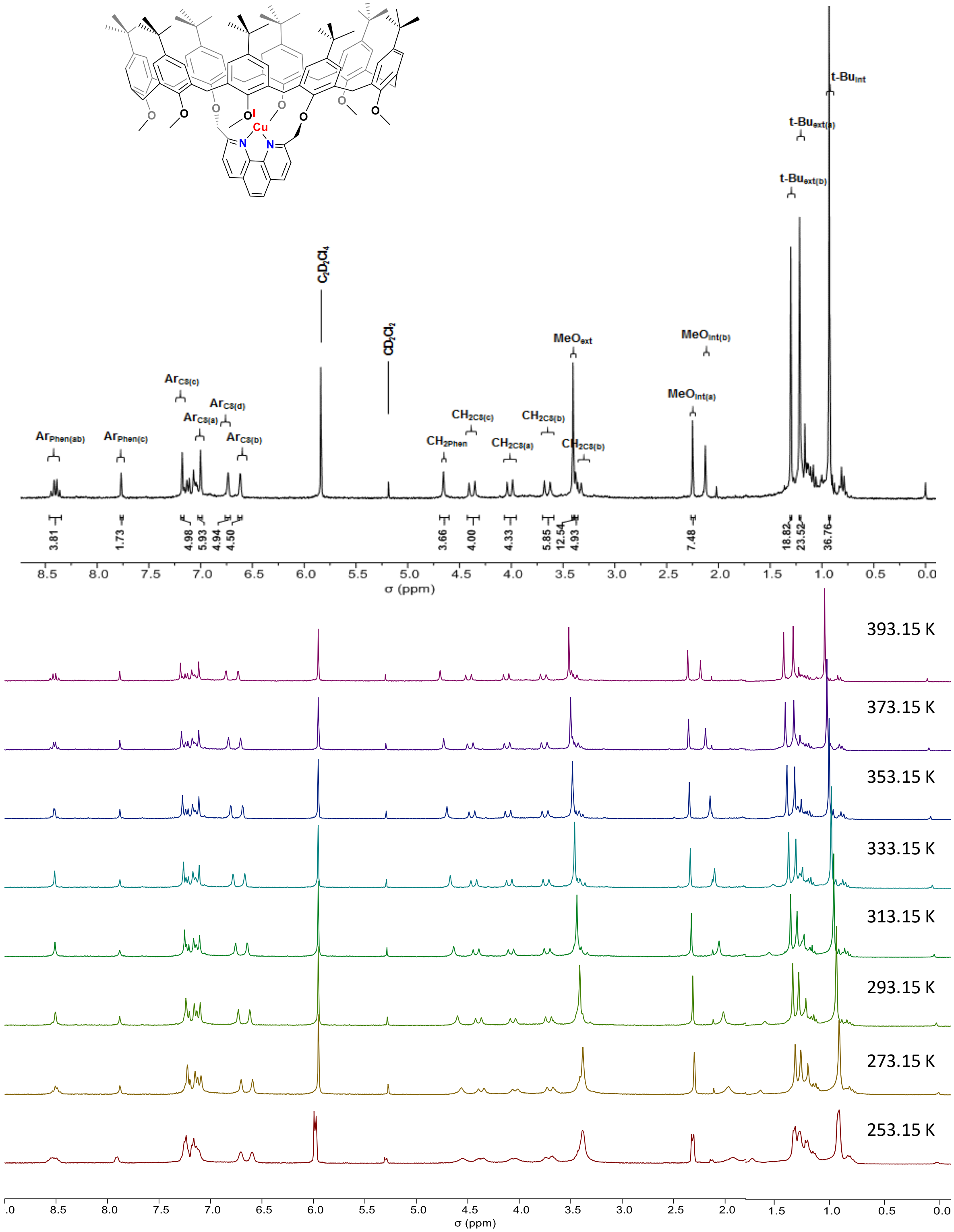

Figure S7. Top: analysis of the ^{1}H NMR spectrum of **[Cu(C$_8$PhenMe$_6$)I]** in $C_2D_2Cl_4$ at 393.15 K. Bottom: VT ^{1}H NMR spectra in $C_2D_2Cl_4$ from 253.15 to 393.15 K.

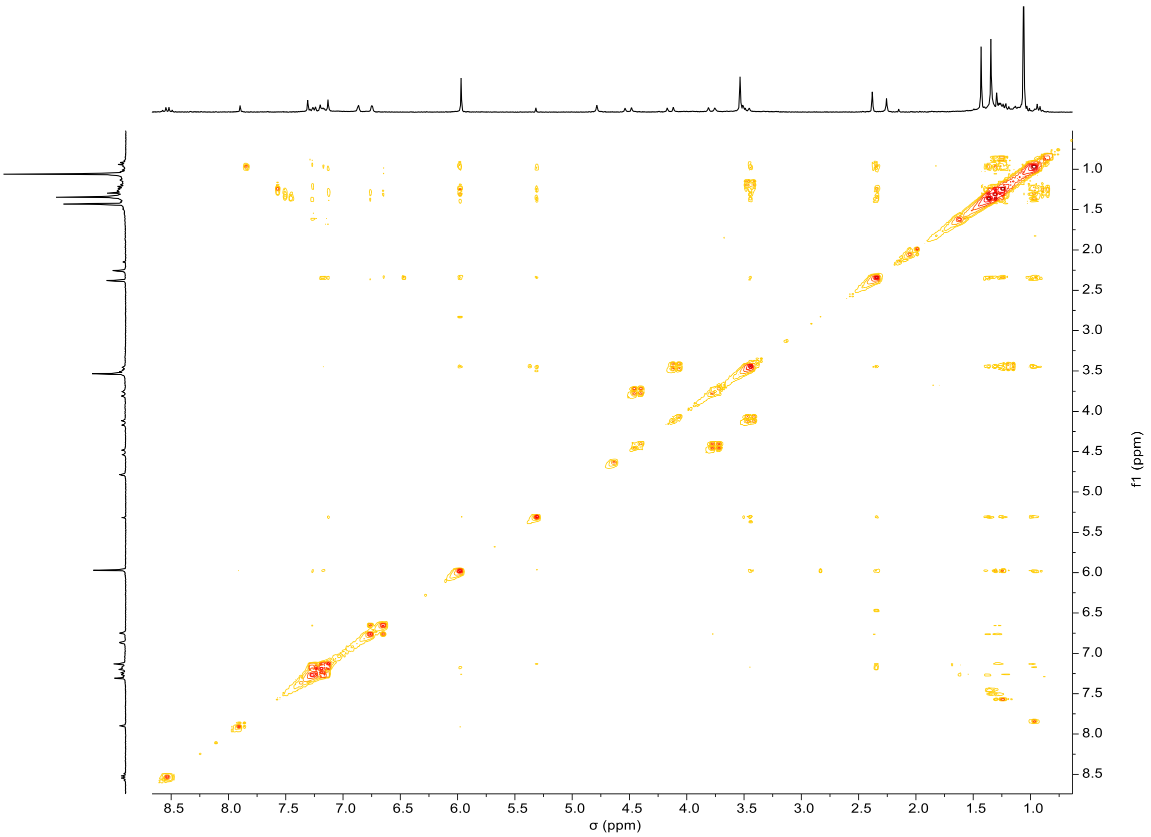

Figure S8. COSY NMR spectrum of **[Cu(C8PhenMe6)I]** in $C_2D_2Cl_4$ at 298.15 K.

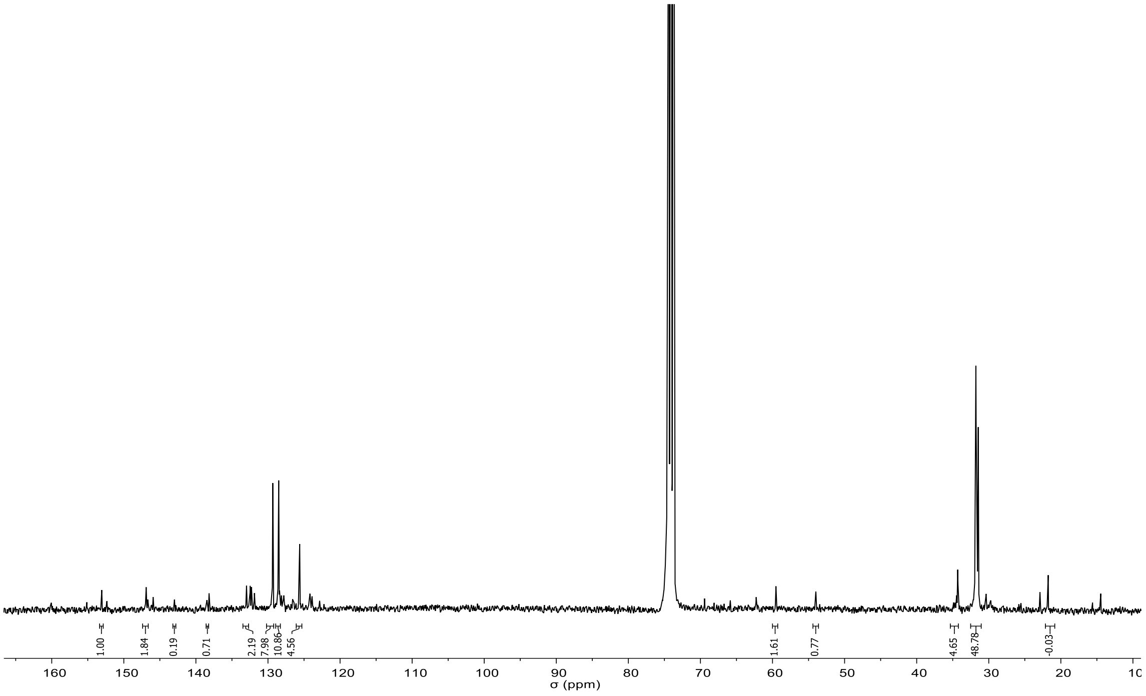

Figure S9. ^{13}C NMR spectrum of **[Cu(C8PhenMe6)I]** in $C_2D_2Cl_4$ at 293.15 K.

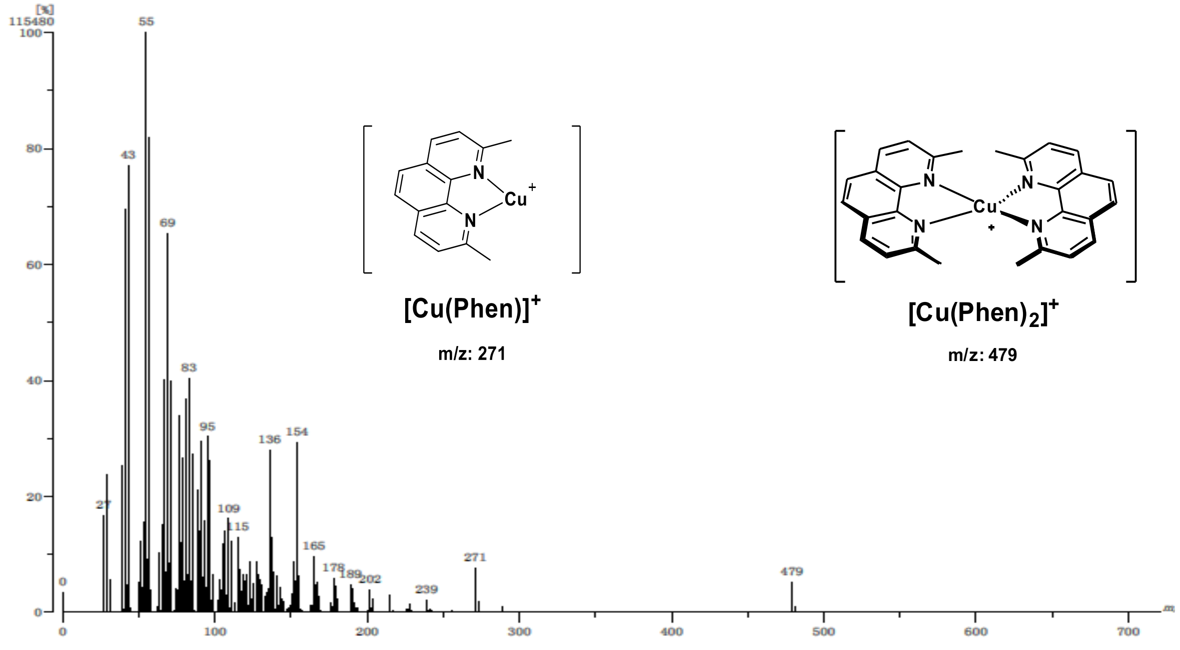

Figure S10. DART MS of **[Cu(Phen)I]**$_2$ in $CHCl_3$.

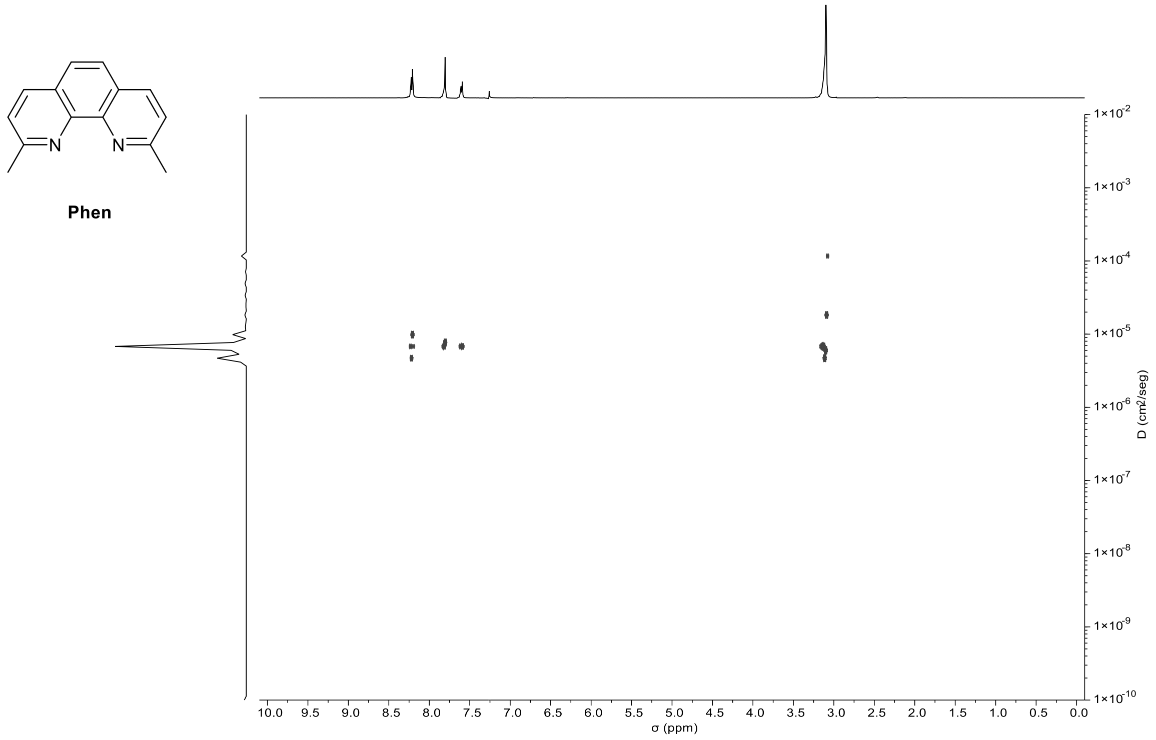

Figure S11. ^{1}H DOSY spectrum of the ligand **Phen** (8 mM in $CDCl_3$|toluene-d_8 500 μL|50 μL) at 298 K, diffusion = 6.80 x 10^{-6} cm^2/s, r_H = 2.38 Å.

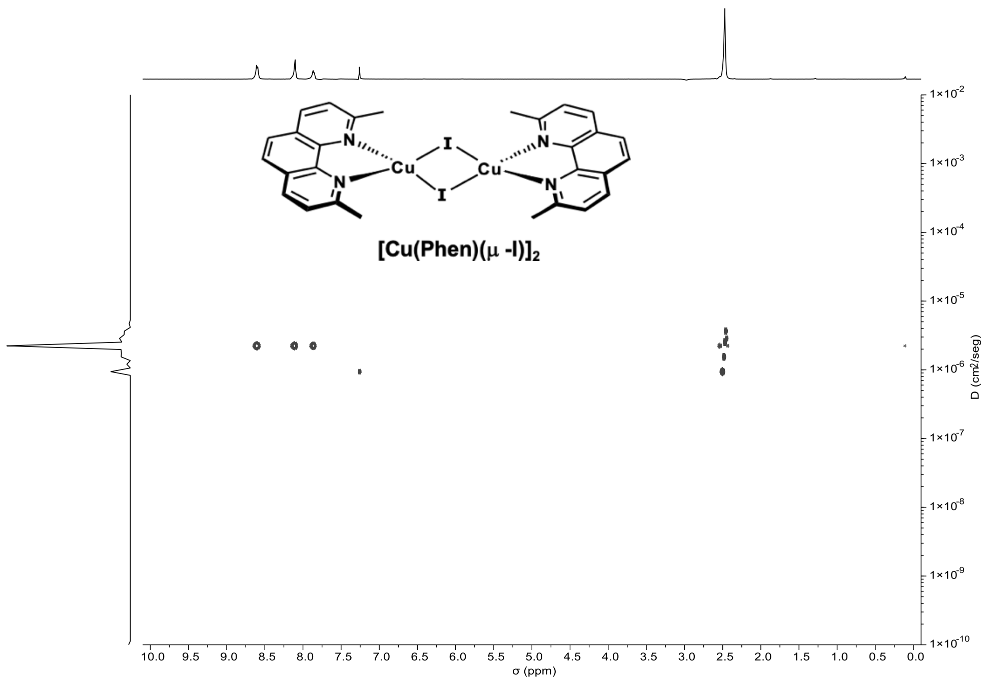

Figure S12. ^{1}H DOSY NMR spectrum of **[Cu(Phen)(μ-I)]$_2$** (8 mM in $CDCl_3$|toluene-d_8 500 μL|50 μL) at 298 K, diffusion = 2.23 x 10^{-6} cm^2/s, r_H = 7.25 Å.

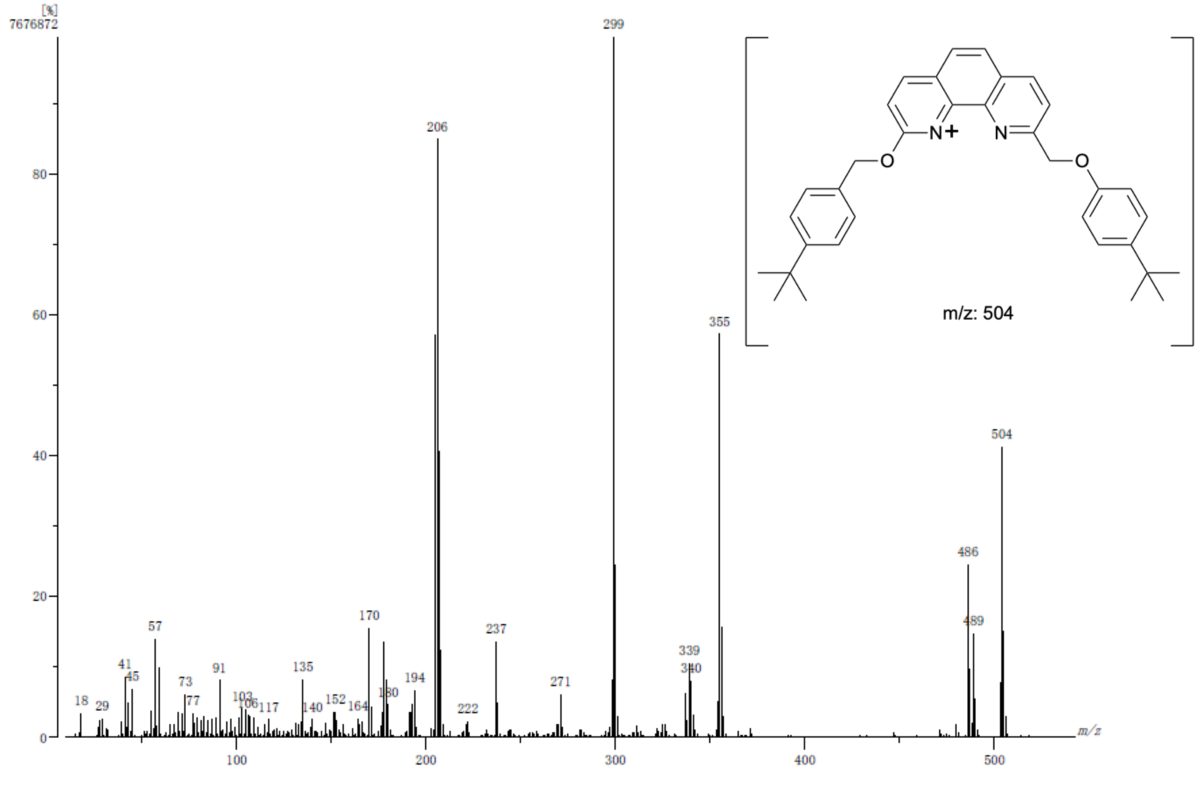

Figure S13. DART MS of **Phen(OAr)$_2$.**

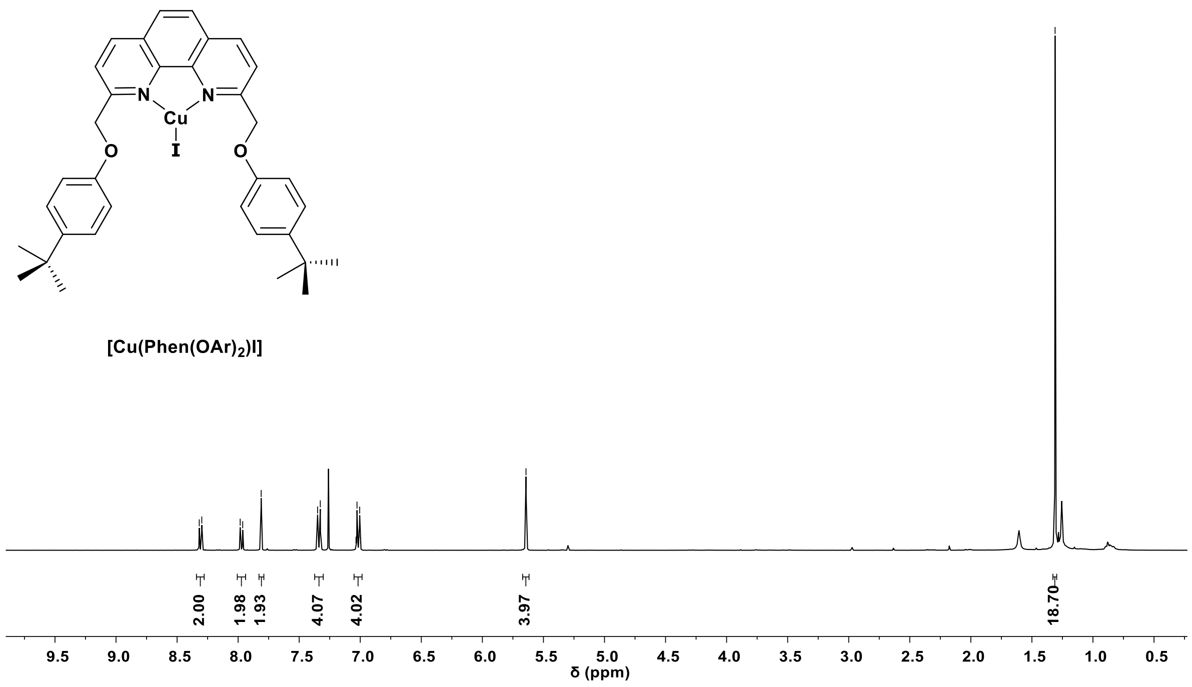

Figure S14. ^{1}H NMR spectrum of **[Cu(Phen(OAr)$_2$I]** in $CDCl_3$ at 293.15 K.

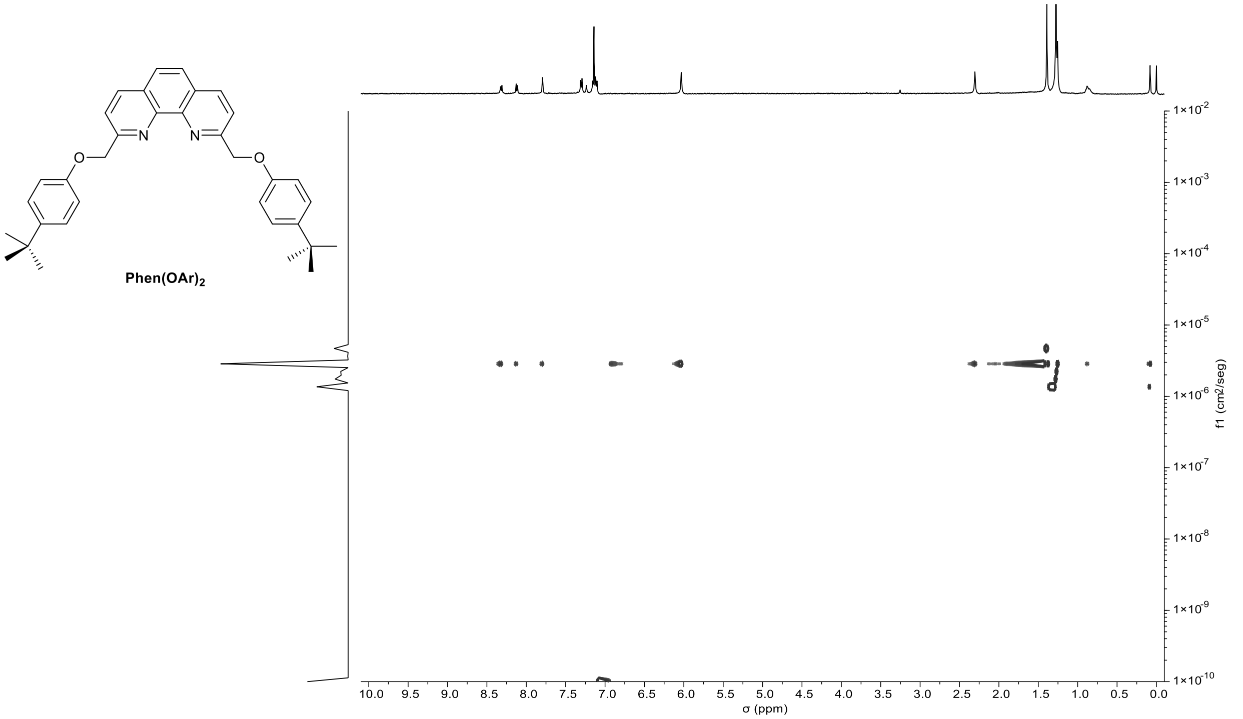

Figure S15. ^{1}H DOSY spectrum of **Phen(OAr)$_2$** (8 mM in $CDCl_3$|toluene-d_8 500 μL|50 μL) at 298 K, diffusion = 2.86 x 10^{-6} cm^2/s, r_H = 5.65 Å.

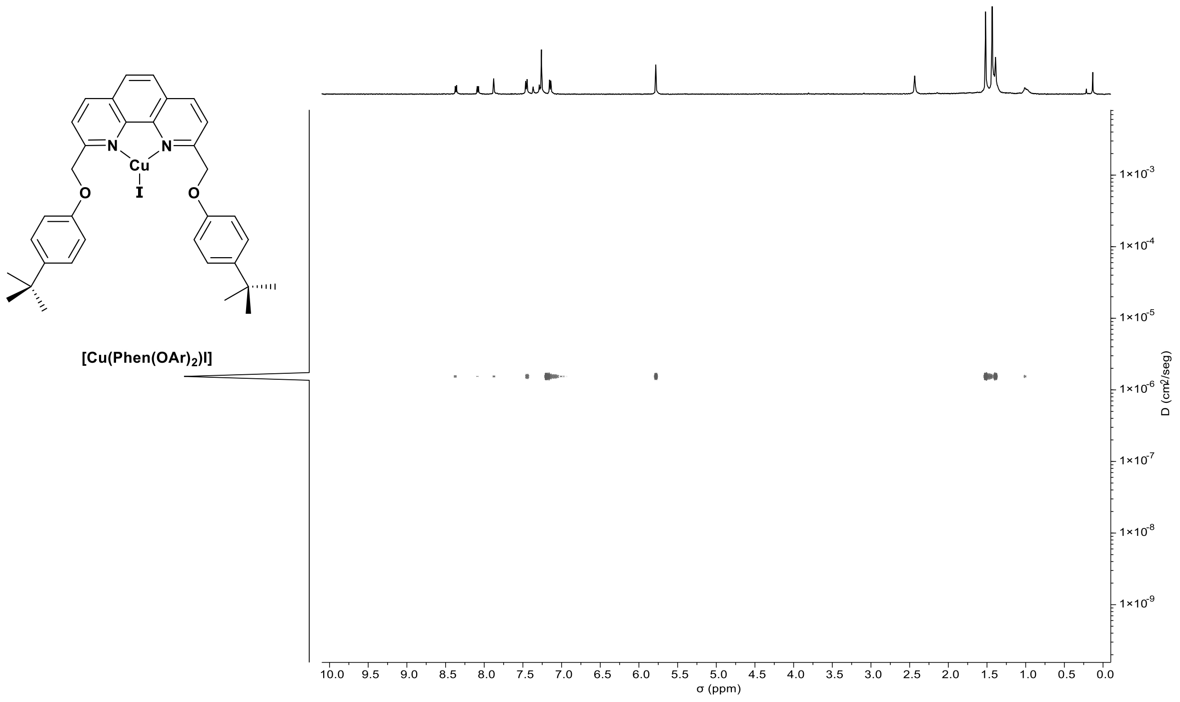

Figure S16. DOSY NMR spectrum of **[Cu(Phen(OAr)$_2$I]** (8 mM in $CDCl_3$|toluene-d_8 500 μL|50 μL) at 298 K, diffusion = 1.53 x 10^{-6} cm^2/s, r_H = 10.56 Å.

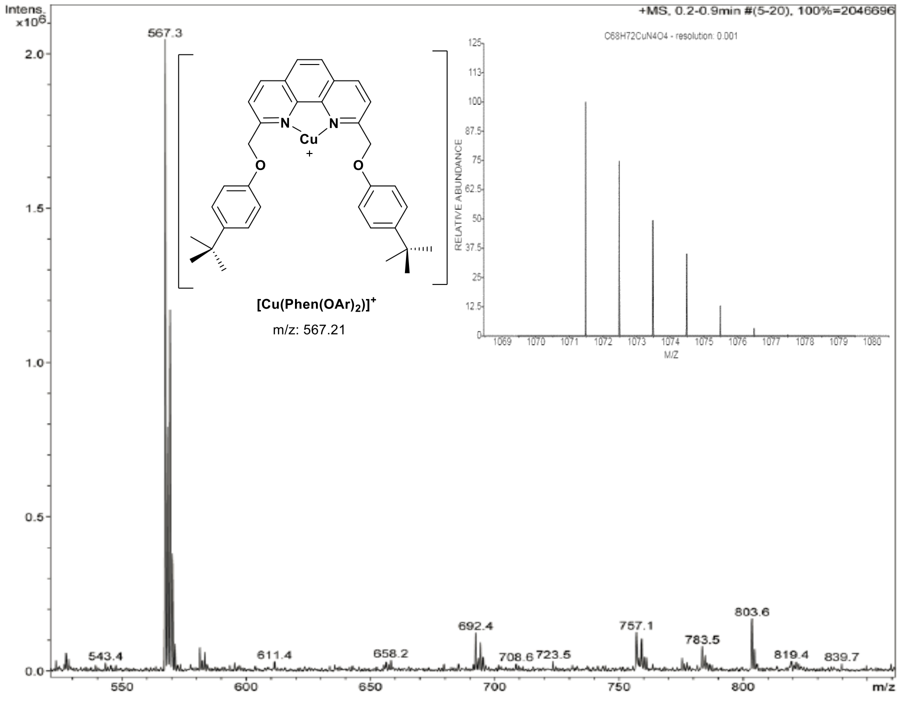

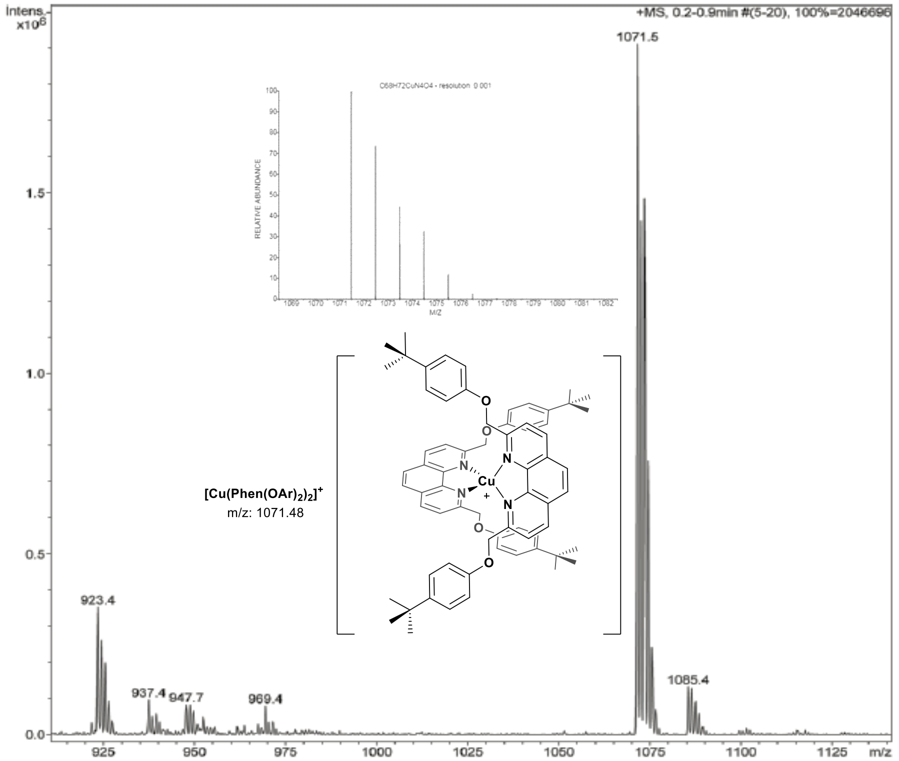

Figure S17. Top: ESI MS of **[Cu(Phen(OAr)$_2$)]$^+$** and its calculated isotopic pattern. Bottom: high *m/z* portion of ESI MS showing **[Cu(Phen(OAr)$_2$)$_2$]$^+$** and its calculated isotopic pattern.

S.4. General procedure for C-N cross-coupling reactions and characterization of the coupling products

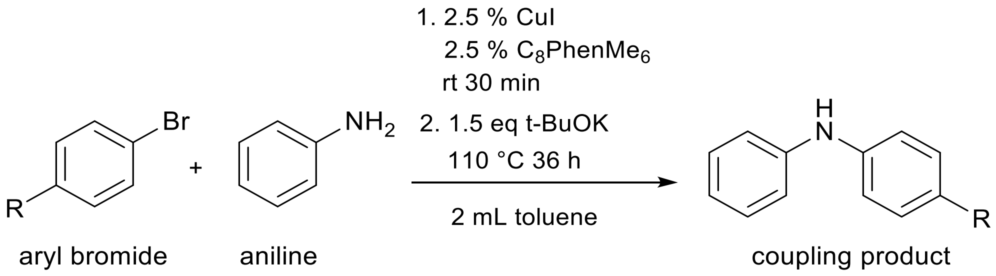

Scheme 7. General coupling procedure.

A 25 mL Schlenk flask was charged with 0.02 mmol (4 mg, 2.5 % mol) of CuI and 0.02 mmol (30 mg, 2.5 % mol) of **C_8PhenMe$_6$** and 2.0 mL of toluene, and the mixture was stirred for 30 min under dinitrogen. After addition of 0.80 mmol (73 μL, 74 mg) of aniline, 0.80 mmol of the corresponding aryl bromide, and 1.2 mmol (150 mg) of *t*-BuOK, the temperature was raised to 110 °C in an oil bath. Reactions were monitored by TLC carried out on 0.25 mm plates coated with silica gel, using UV light (254 nm) as visualising agent, until the consumption of the starting materials, or when a significant amount of the likely coupling products was evident, then the reaction was quenched by exposing it to air. The crude products were extracted with diethyl ether, separated and isolated by flash column chromatography performed on aluminium oxide 90 basic Macherey-Nagel as the stationary phase and hexane-dichloromethane at different ratios as the eluent. Yields refer to isolated compounds and their identity was confirmed by comparison with literature spectroscopic data. The same conditions were employed for comparison with cavitand **C_8Phen**, the catalyst blank **[Cu(Phen)I]$_2$**, and the molecular analogue **[Cu(Phen(OAr)$_2$)I]**; mercury drop tests were carried out under the same conditions.

S.4.1. Optimisation of the C-N cross-coupling reaction

Table S1. Optimization of the reaction conditions for C-N cross-coupling.[a]

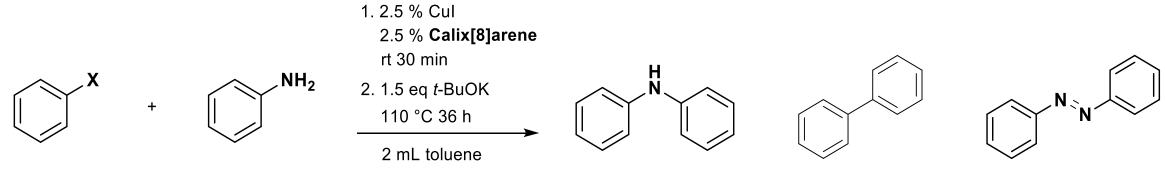

Entry	Ligand	Aryl halide	Base	Yield[b] (%)		
				1	2	3
1	**$C_8PhenMe_6$**	chlorobenzene	*t*-BuOK	---	---	---
2	**$C_8PhenMe_6$**	bromobenzene	*t*-BuOK	73	8	---
3	**$C_8PhenMe_6$**	iodobenzene	*t*-BuOK	56	10	---
4	**$C_8PhenMe_6$**	bromobenzene	MeONa	20	10	---
5	**$C_8PhenMe_6$**	bromobenzene	NaOH	---	12	---
6	**$C_8PhenMe_6$**	bromobenzene	DBU	---	8	---
7	**$C_8PhenMe_6$**	bromobenzene	Cs_2CO_3	---	10	---
8[c]	**C_8Phen**	bromobenzene	*t*-BuOK	50	12	7
9[c]	**C_8Phen**	iodobenzene	*t*-BuOK	50	12	7
10[d]	**Phen**	bromobenzene	*t*-BuOK	20	10	---
11[d]	**$Phen(OAr)_2$**	bromobenzene	*t*-BuOK	26	8	10
12[e]	Without	bromobenzene	*t*-BuOK	---	9	---
13[f]	**$C_8PhenMe_6$**	bromobenzene	*t*-BuOK	68	6	---
14[g]	**$C_8PhenMe_6$**	bromobenzene	*t*-BuOK	52	12	---
15[h]	**$C_8PhenMe_6$**	bromobenzene	*t*-BuOK	---	---	---
16[i]	**$C_8PhenMe_6$**	bromobenzene	*t*-BuOK	---	---	---

[a] The evaluations were performed in a Schlenk flask (25 mL) with 2 mL of toluene, temperature was raised to 110 °C in an oil bath for 36 h under a nitrogen atmosphere. Reaction conditions: molar ratio of 1:1 of aryl halide and aniline, with 2.5 % mol of ligand and 2.5 % mol of CuI. [b] Isolated yields after column chromatography. [c] Also 15 % of triphenylamine was isolated. [d] 10 % mol of ligand and 10 % mol of CuI. [e] Without ligand and 10 % mol of CuI. [f] Mercury drop-test was performed. [g] THF as solvent. [h] Neat. [i] 2.5 % mol of CuCl.

S.4.2. Characterisation of the C-N coupling products

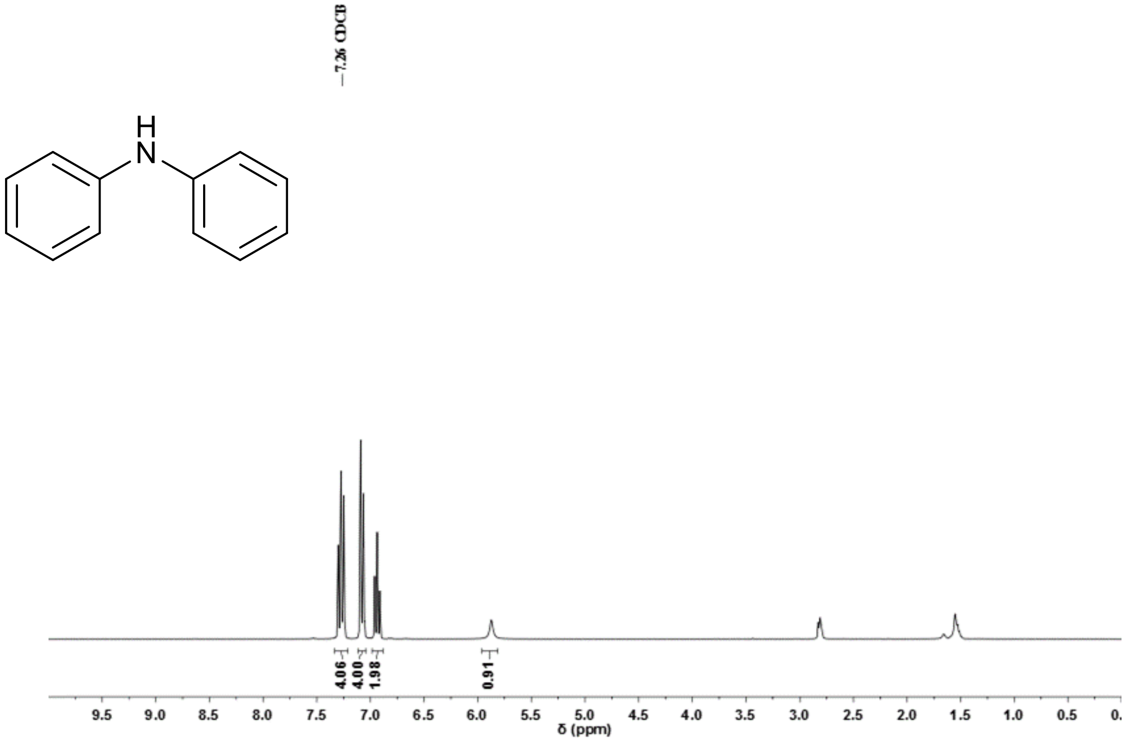

Figure S18. ^{1}H NMR spectrum of diphenylamine.

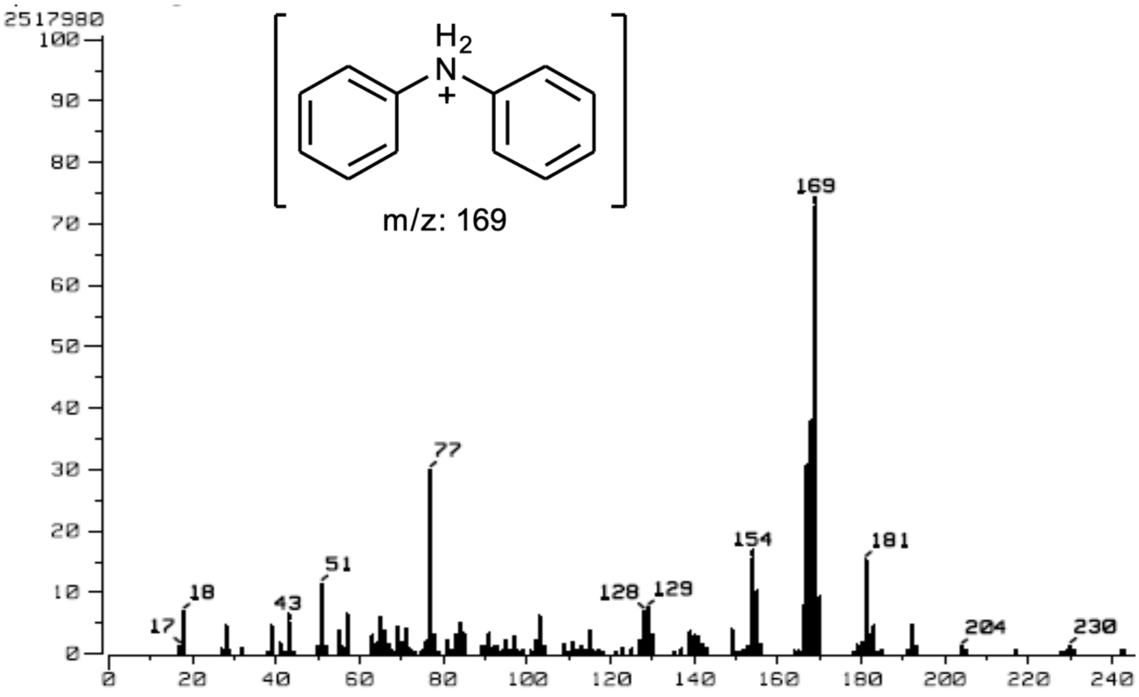

Figure S19. DART MS of diphenylamine.

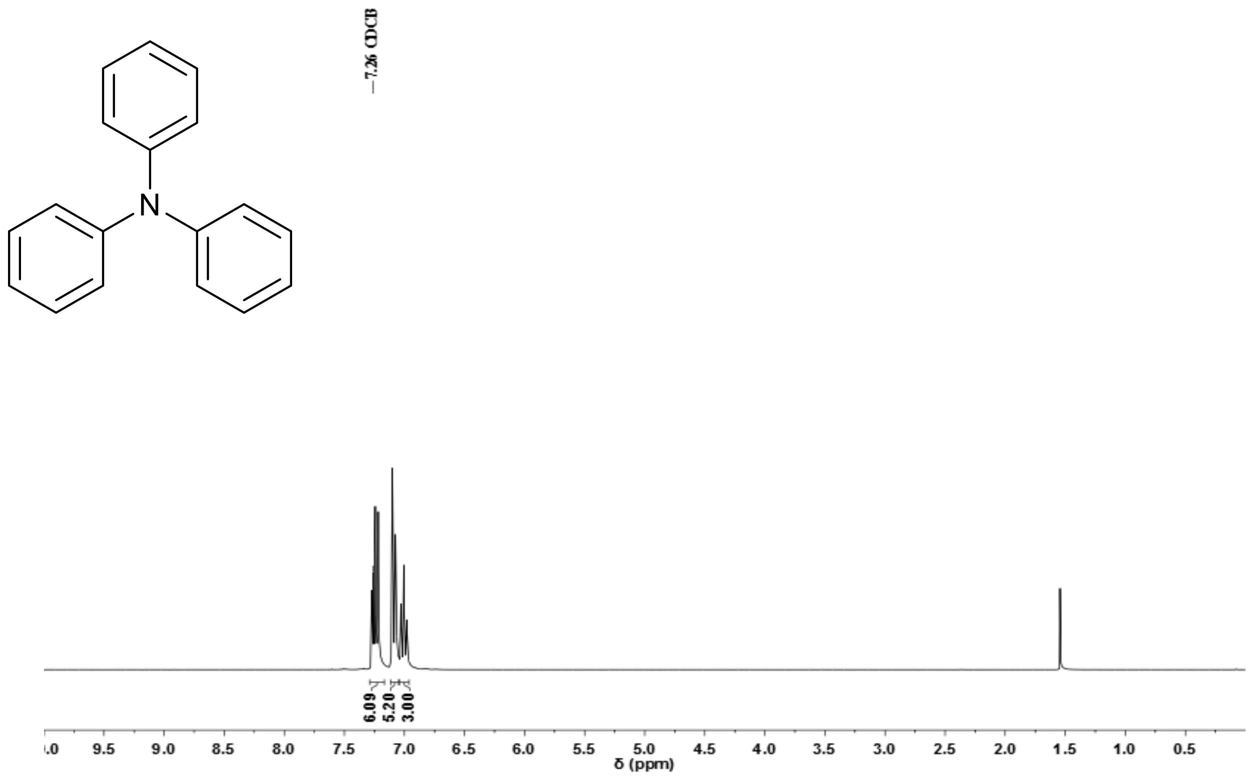

Figure S20. ^{1}H NMR spectrum of triphenylamine.

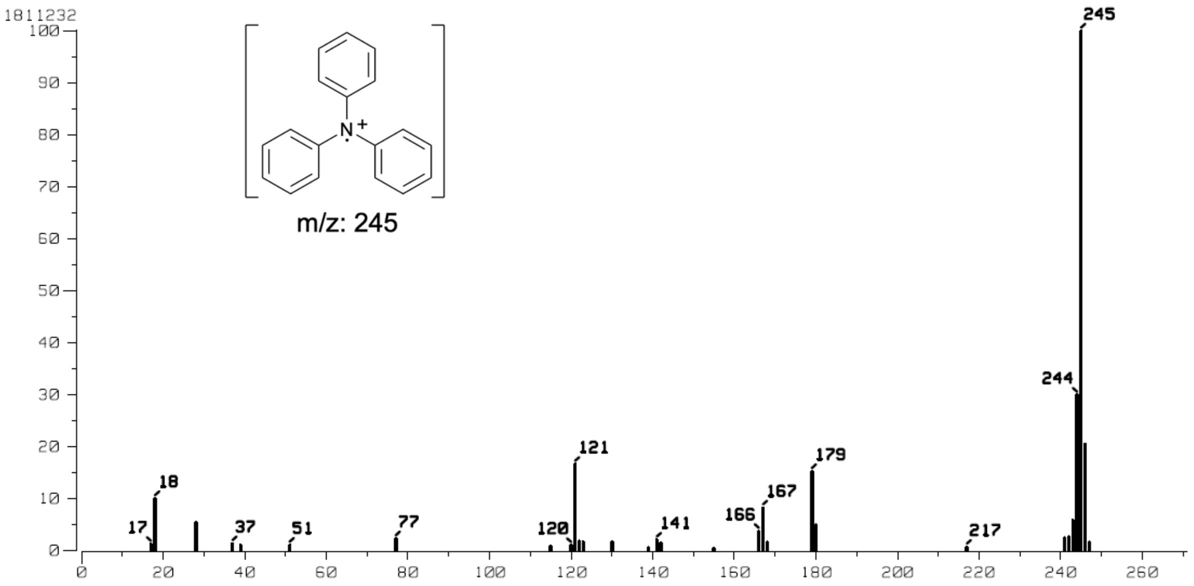

Figure S21. DART MS of triphenylamine.

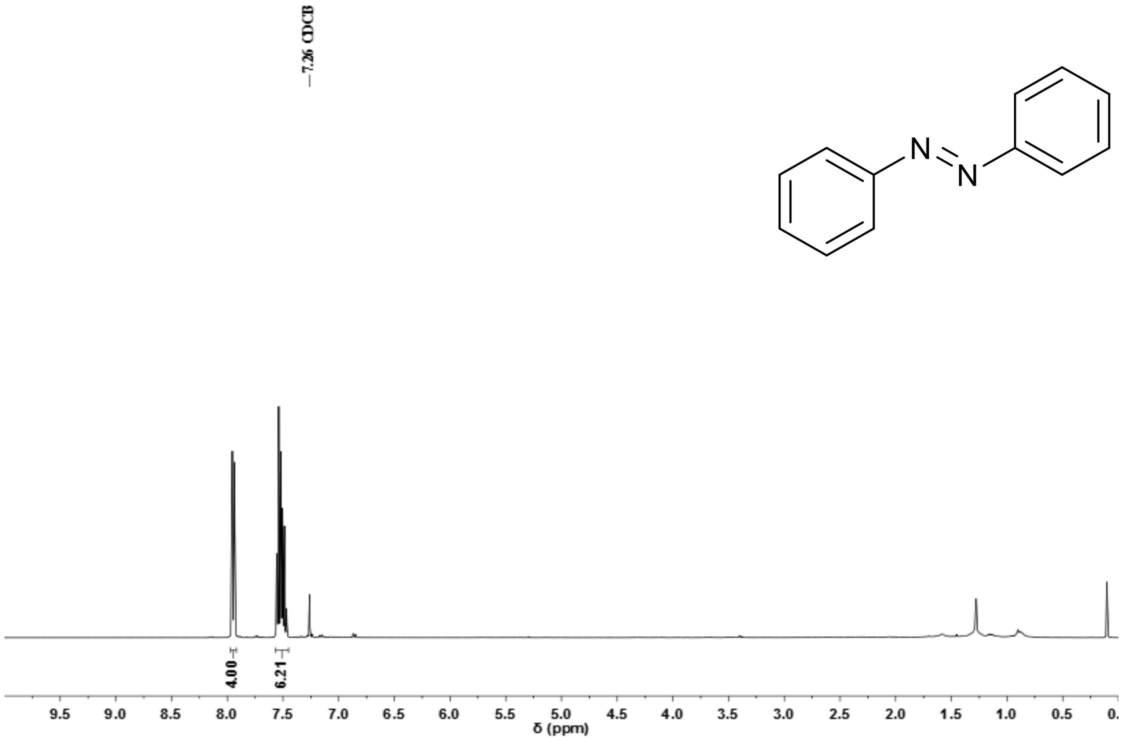

Figure S22. ^{1}H NMR spectrum of azobenzene.

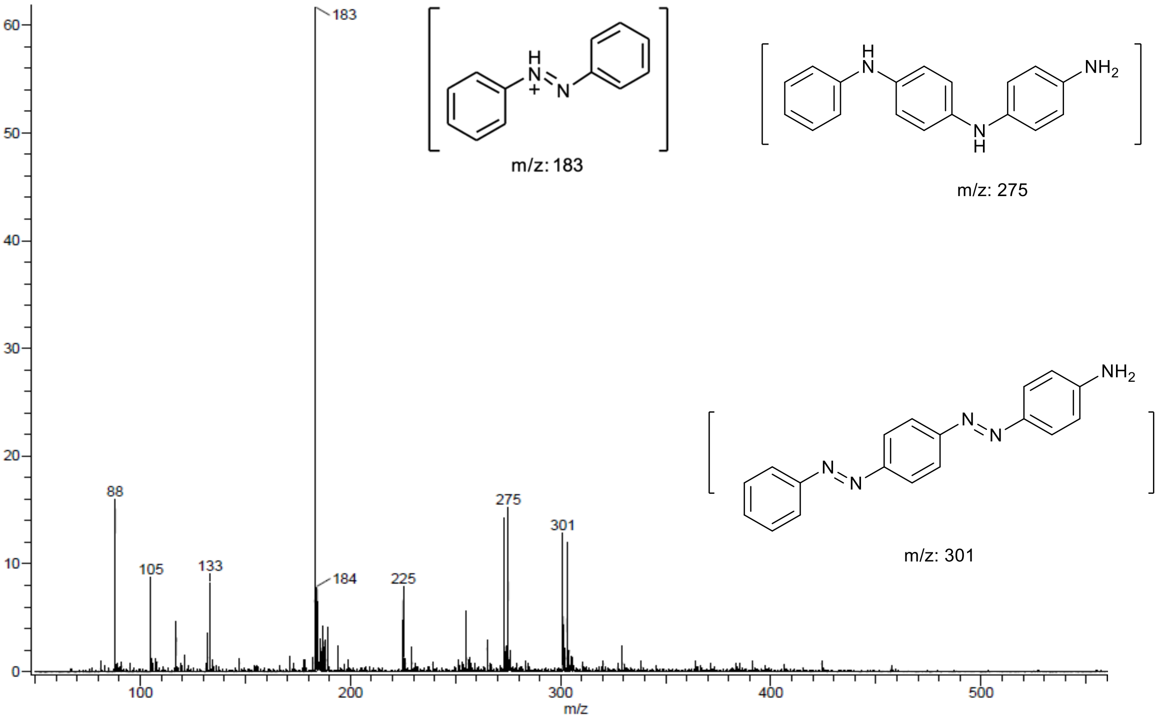

Figure S23. DART MS of azobenzene.

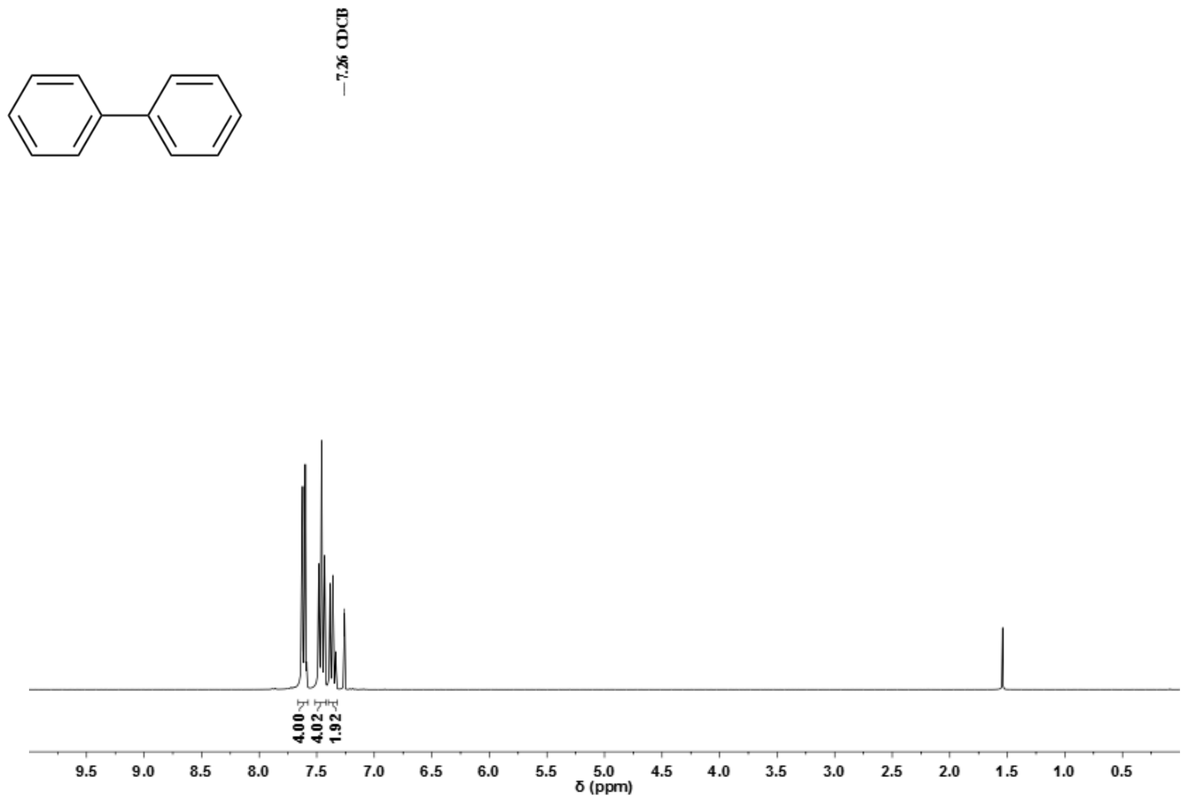

Figure S24. ^{1}H NMR spectrum of biphenyl.

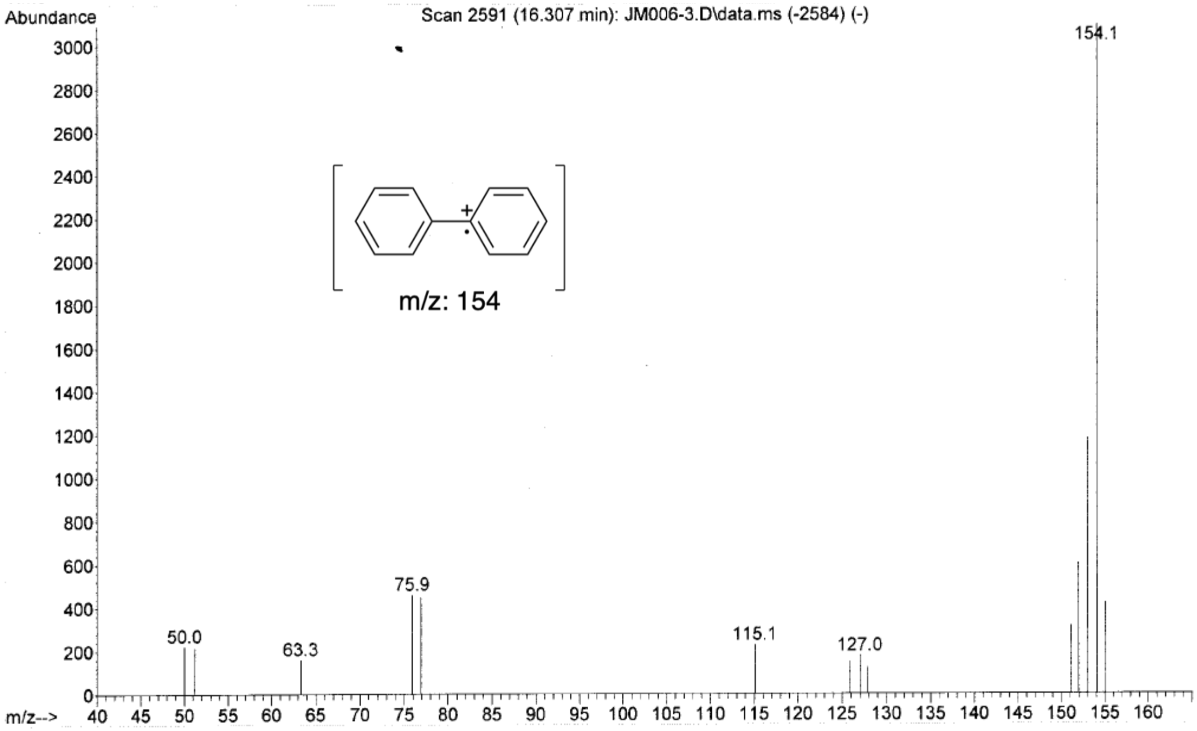

Figure S25. DART MS of biphenyl.

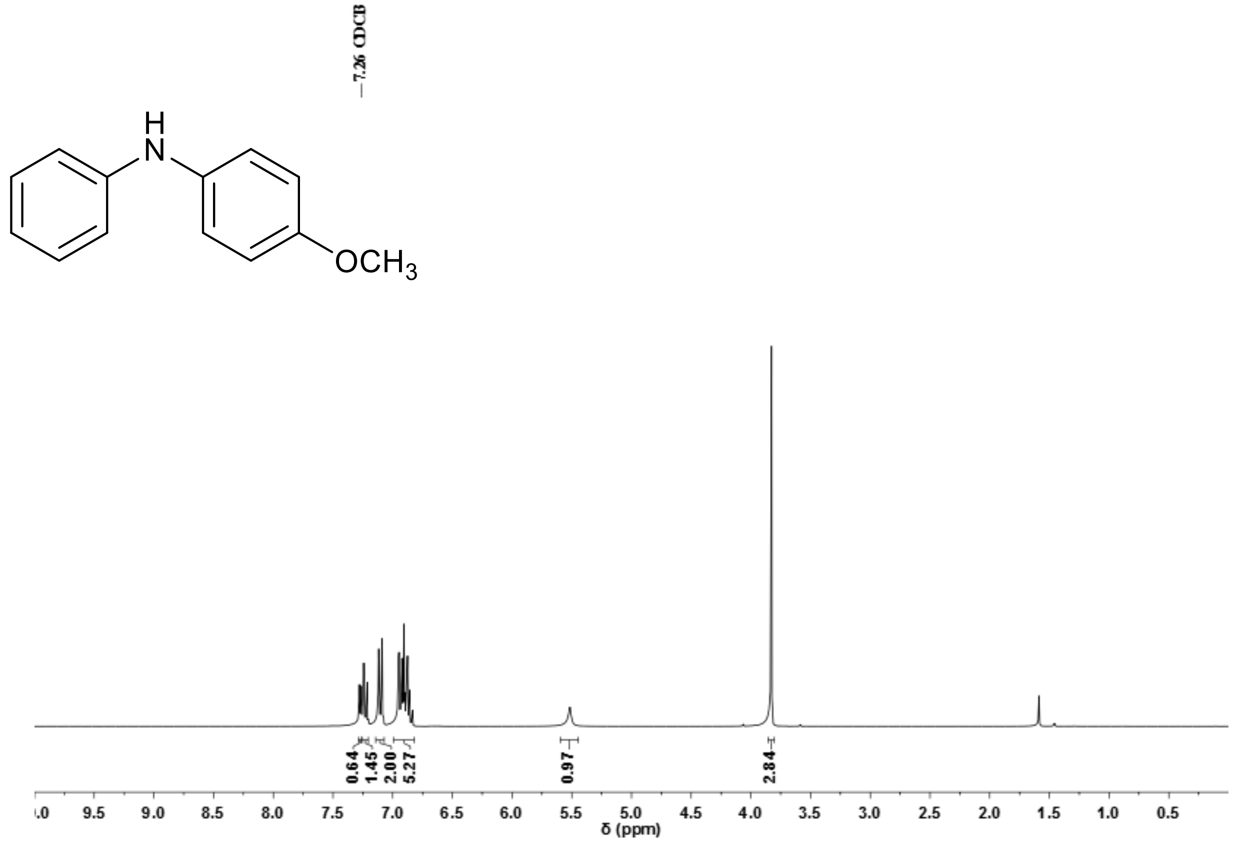

Figure S26. ^{1}H NMR spectrum of 4-methoxydiphenylamine.

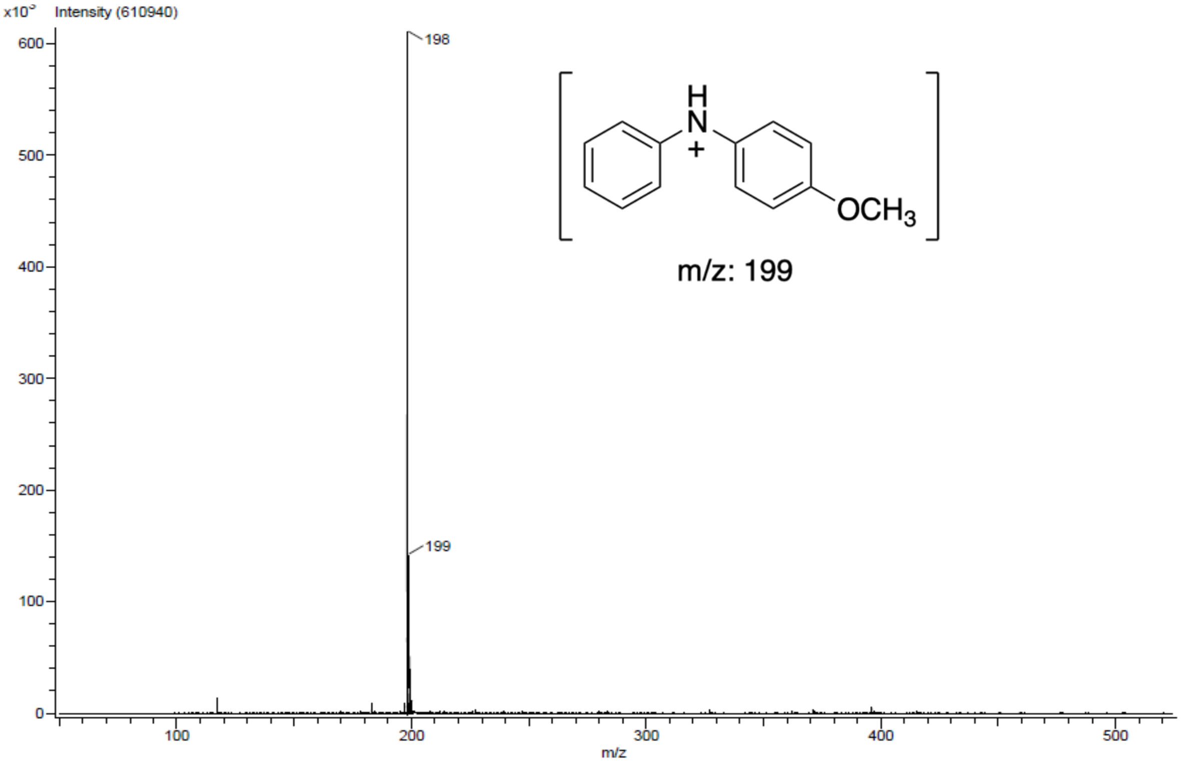

Figure S27. DART MS of 4-methoxydiphenylamine.

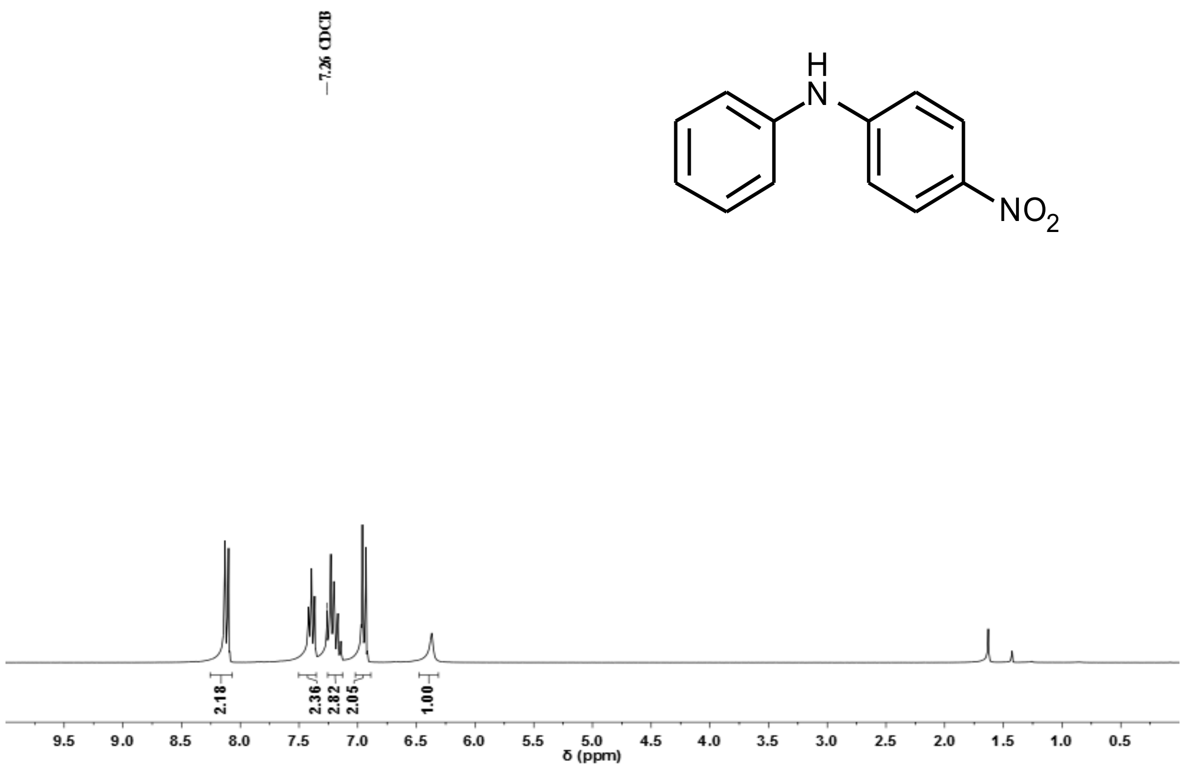

Figure S28. ^{1}H NMR spectrum of 4-nitrodiphenylamine.

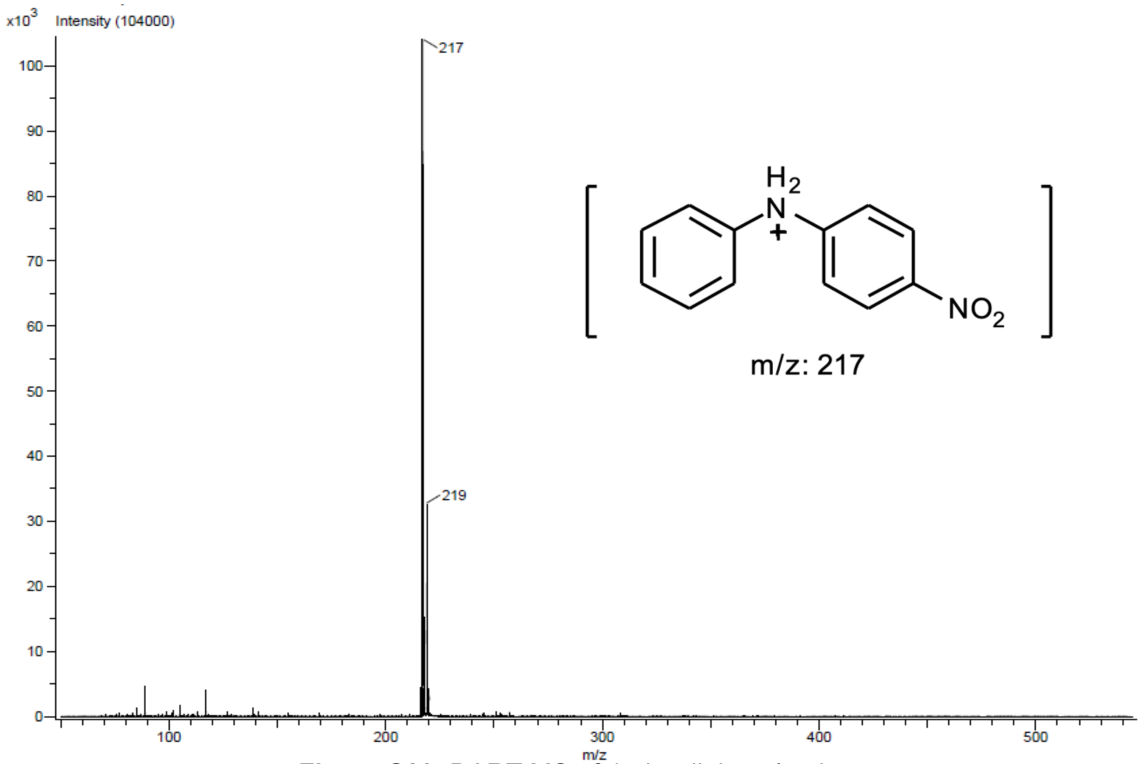

Figure S29. DART MS of 4-nitrodiphenylamine.

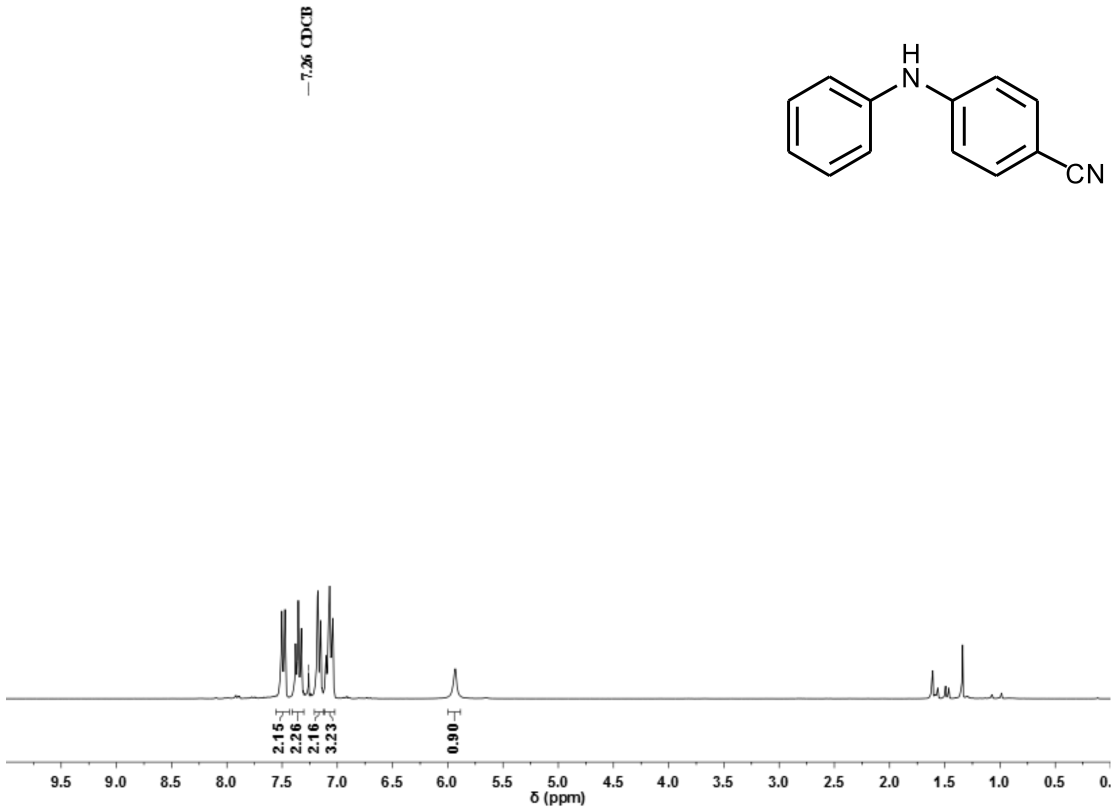

Figure S30. ^{1}H NMR spectrum of 4-cyanodiphenylamine.

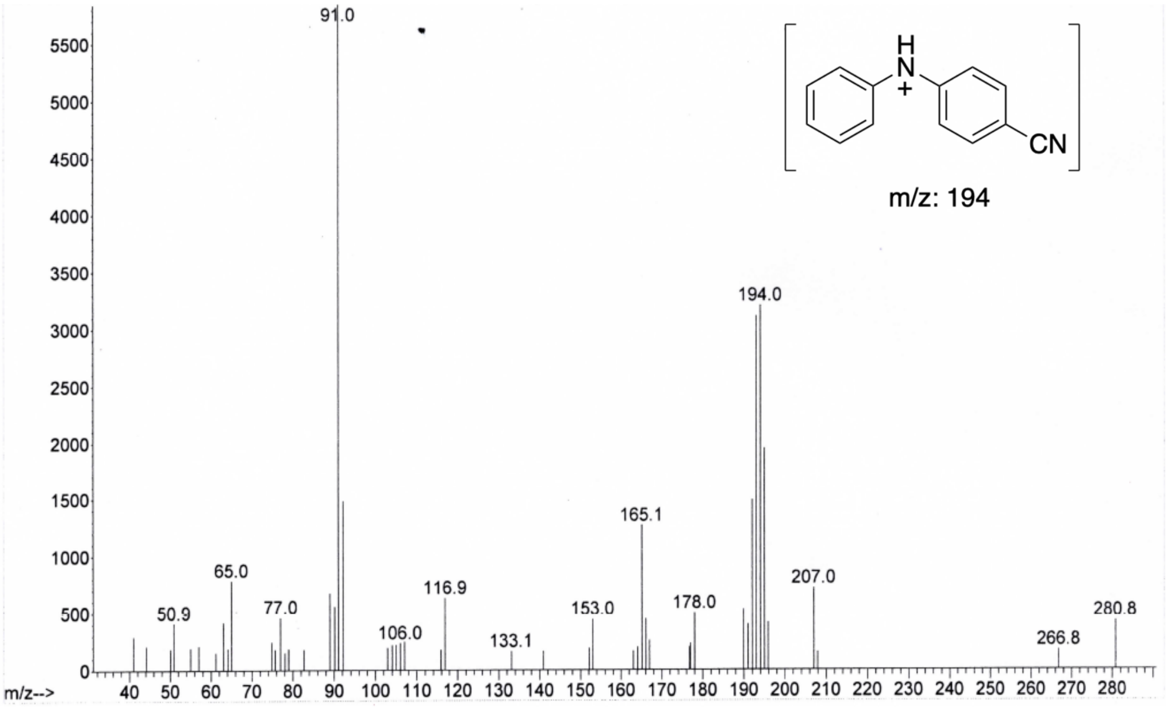

Figure S31. DART MS of 4-cyanodiphenylamine.

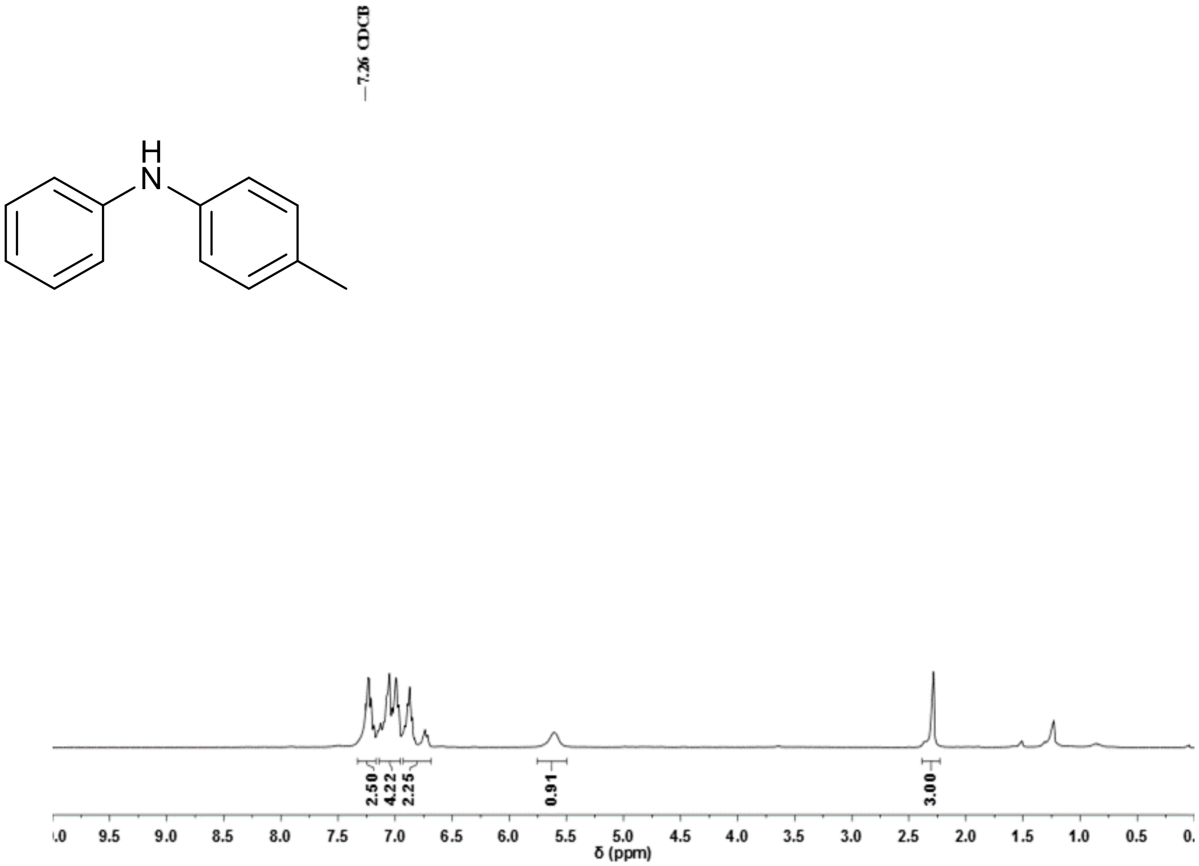

Figure S32. ^{1}H NMR spectrum of 4-methyldiphenylamine.

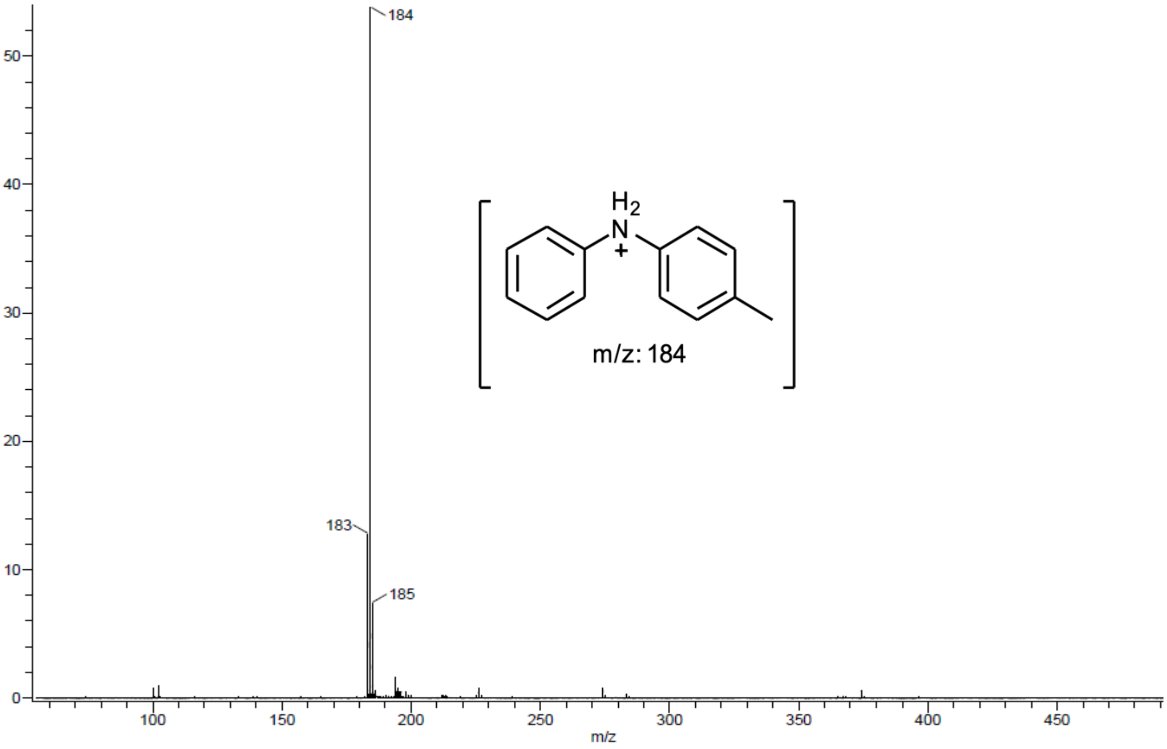

Figure S33. DART MS of 4-methyldiphenylamine.

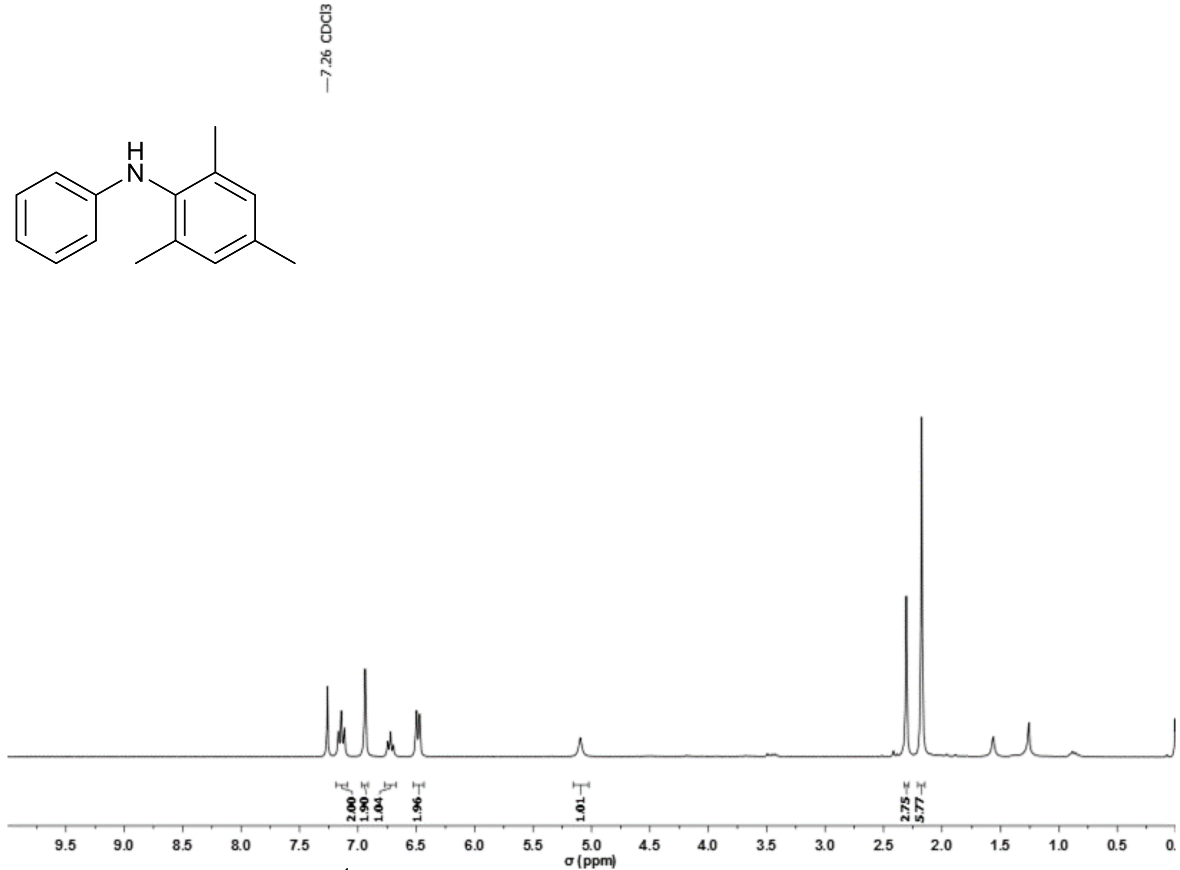

Figure S34. ^{1}H NMR spectrum of 2,4,6-trimethyldiphenylamine.

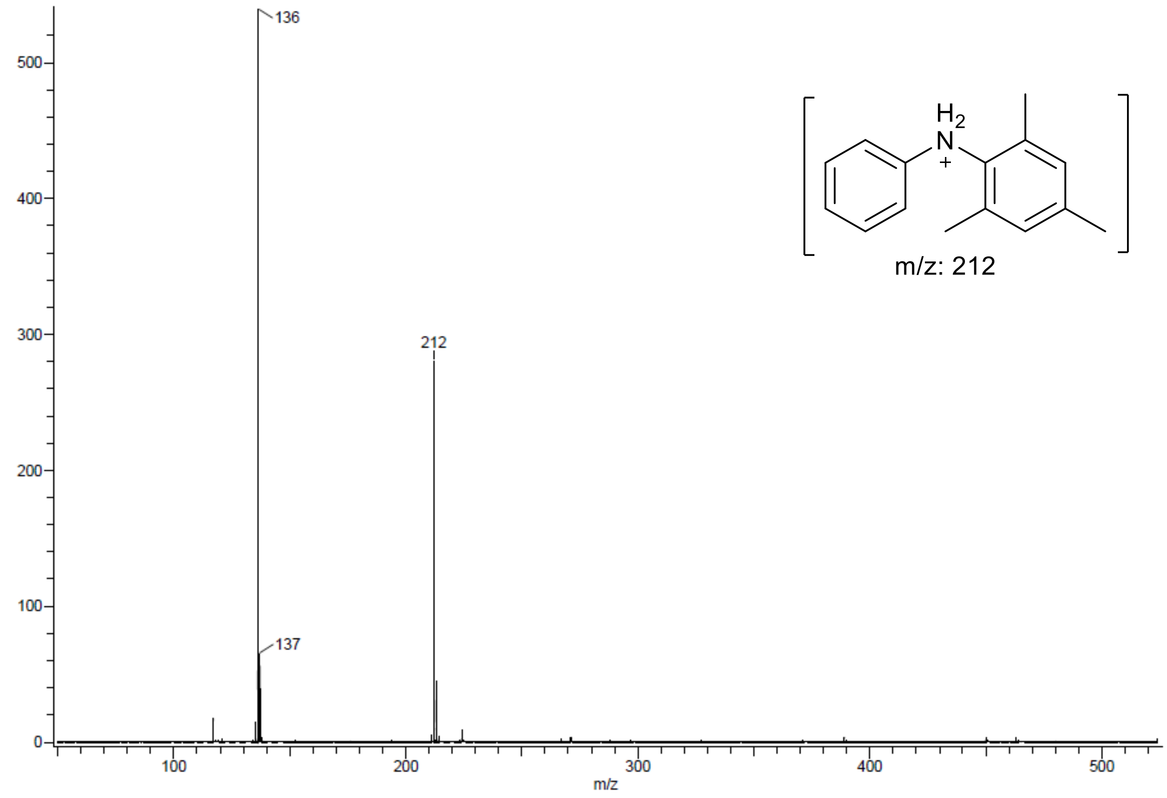

Figure S35. DART MS of 2,4,6-trimethyldiphenylamine.

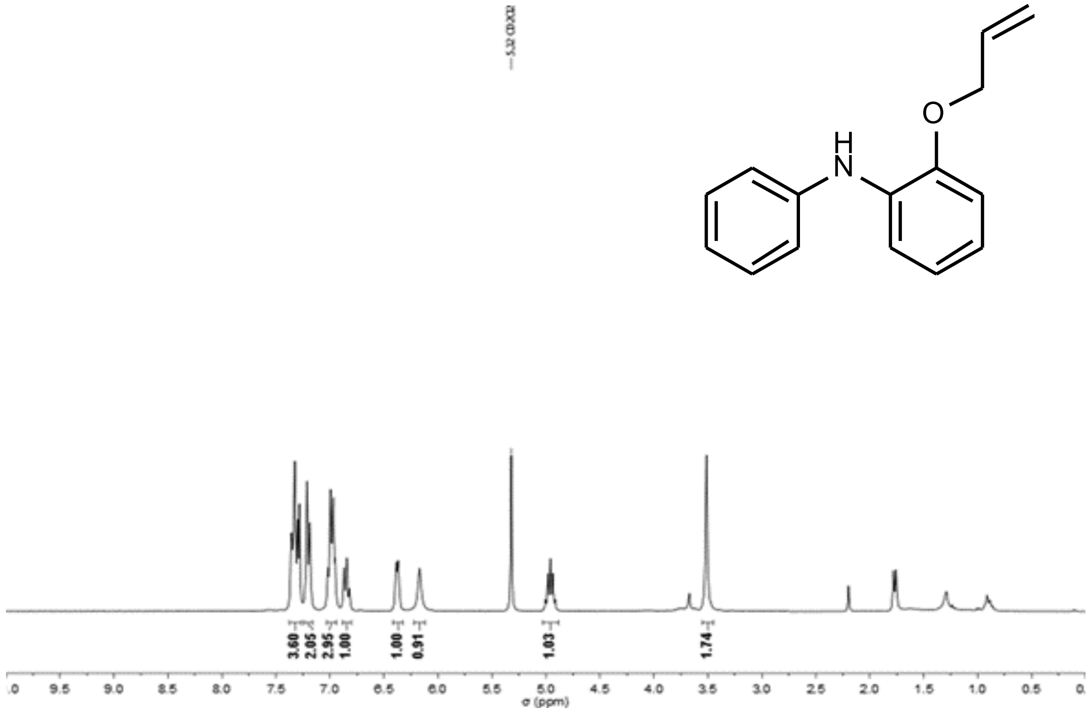

Figure S36. ^{1}H NMR spectrum of 2-(allyloxy)-N-phenylaniline.

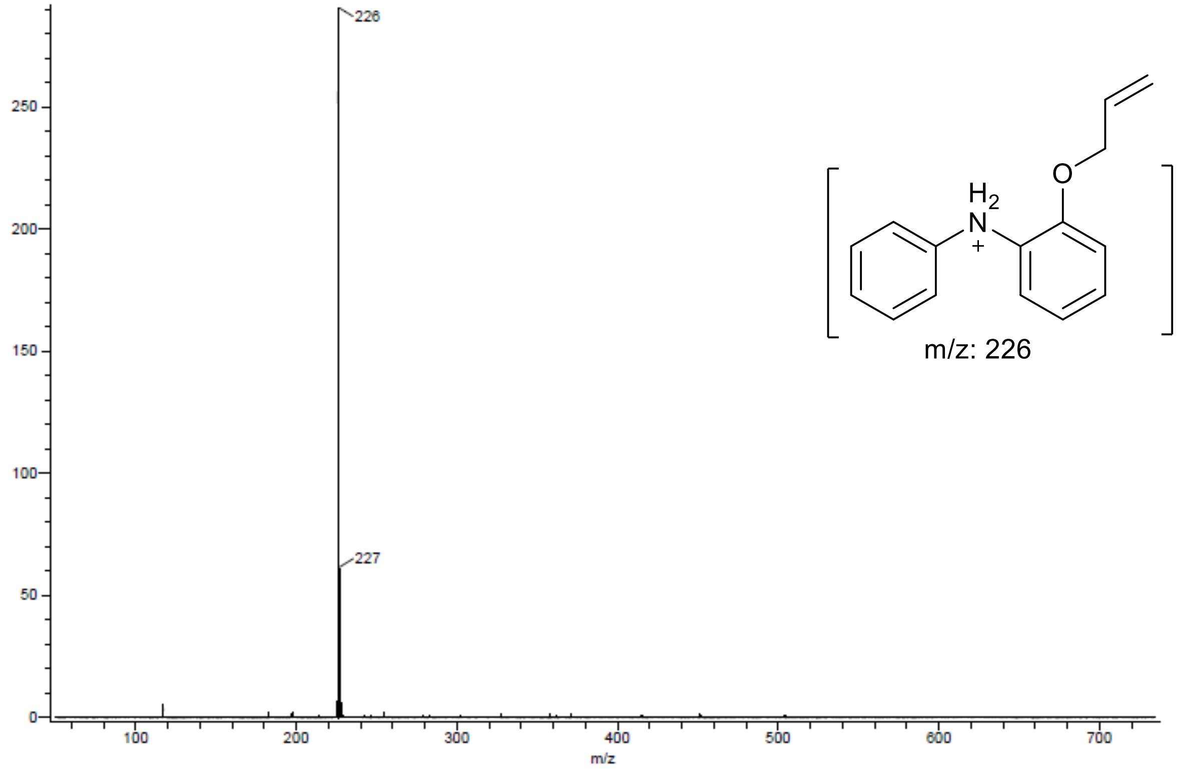

Figure S37. DART MS of 2-(allyloxy)-N-phenylaniline.

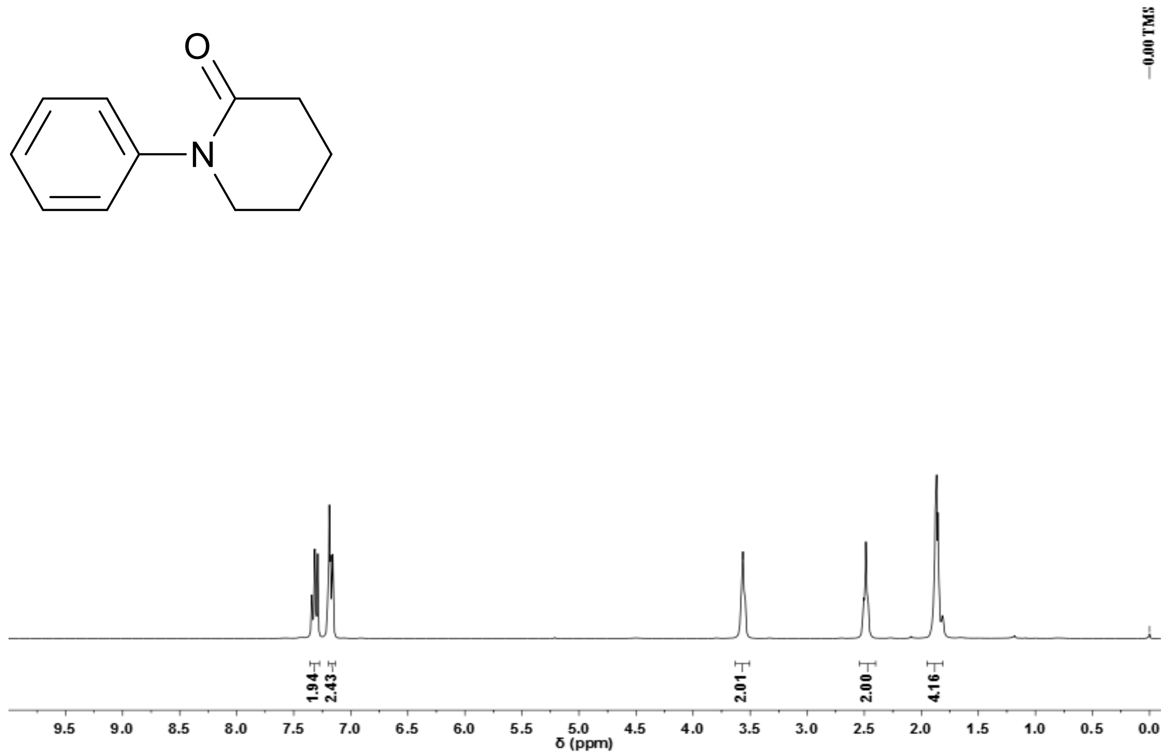

Figure S38. ^1H NMR spectrum of 1-phenyl-2-piperidinone.

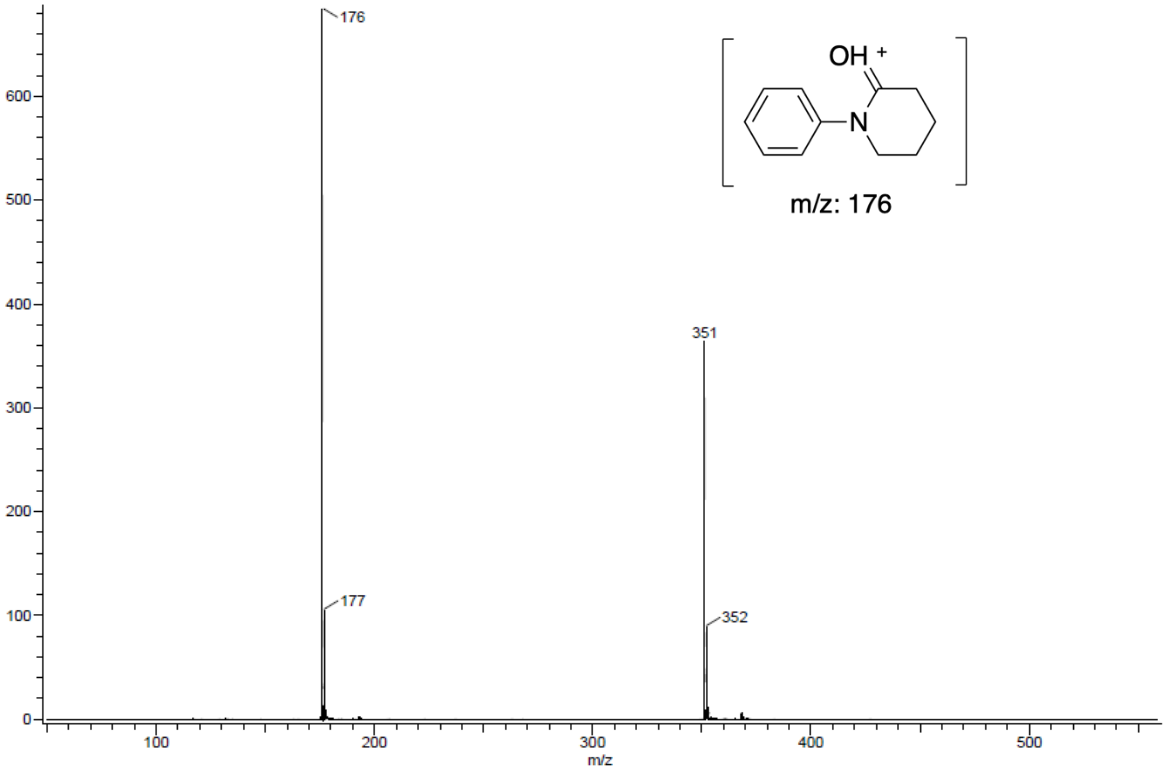

Figure S39. DART MS of 1-phenyl-2-piperidinone.

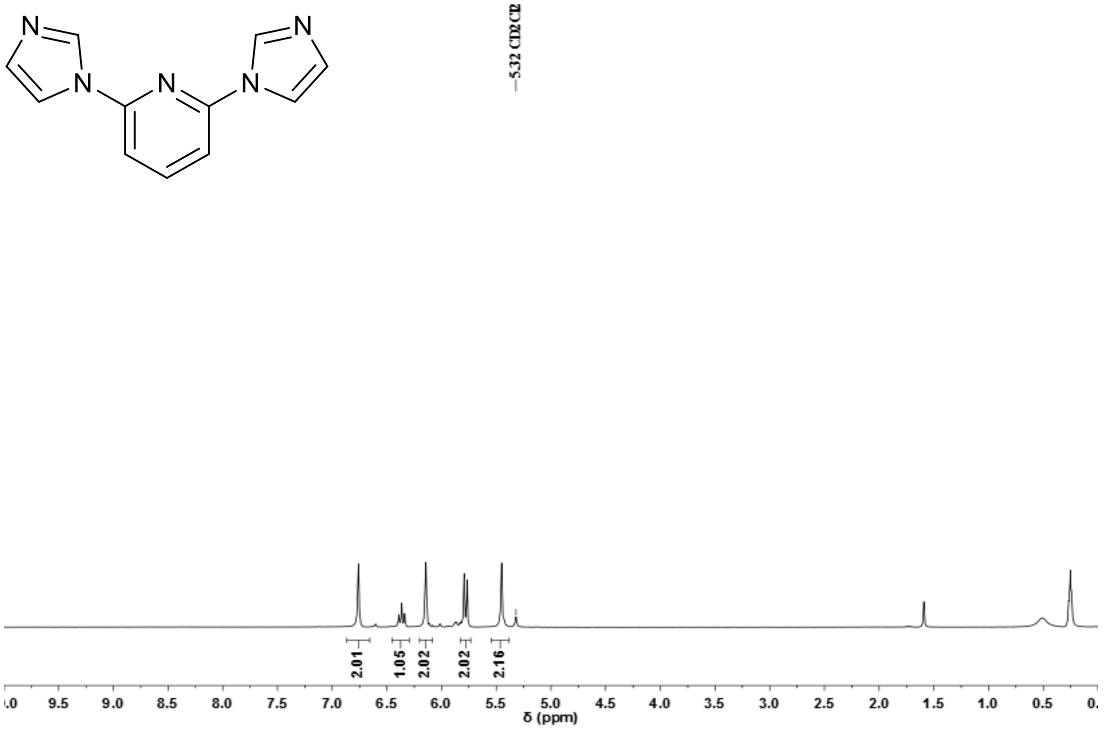

Figure S40. ^{1}H NMR spectrum of 2,6-di(1H-imidazol-1-yl)pyridine.

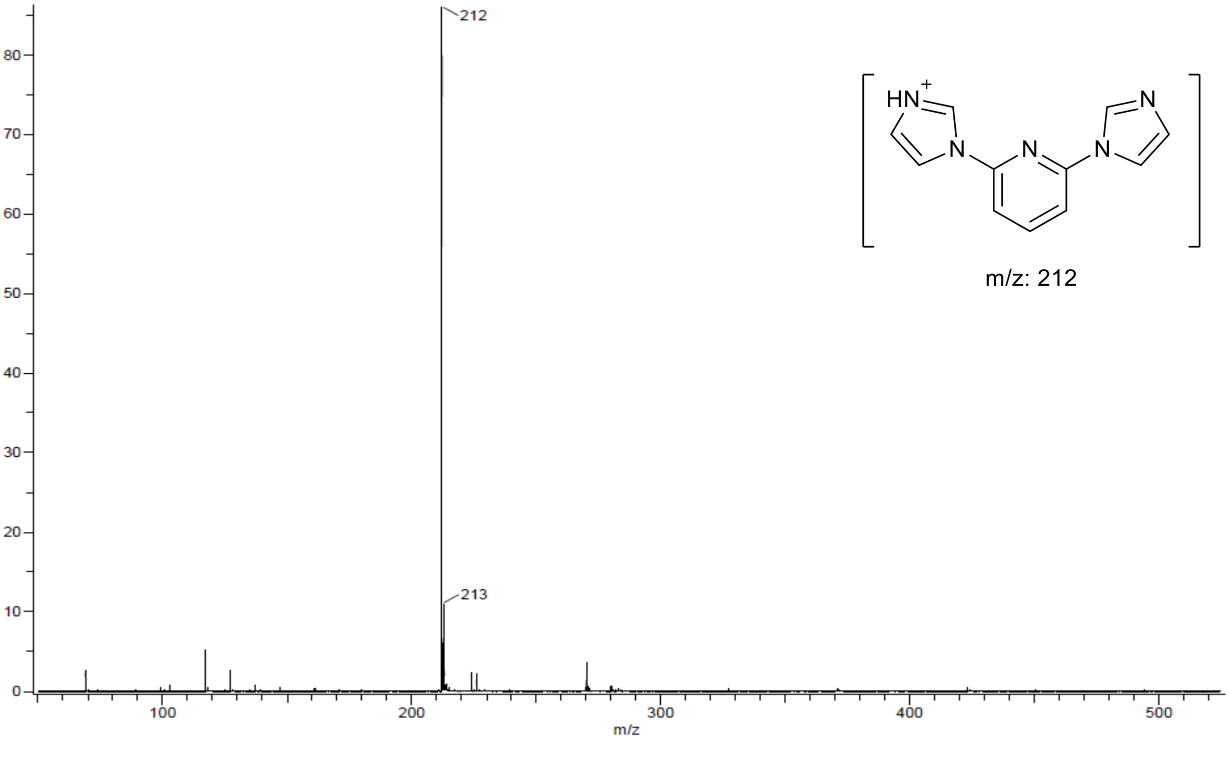

Figure S41. DART MS of 2,6-di(1H-imidazol-1-yl)pyridine.

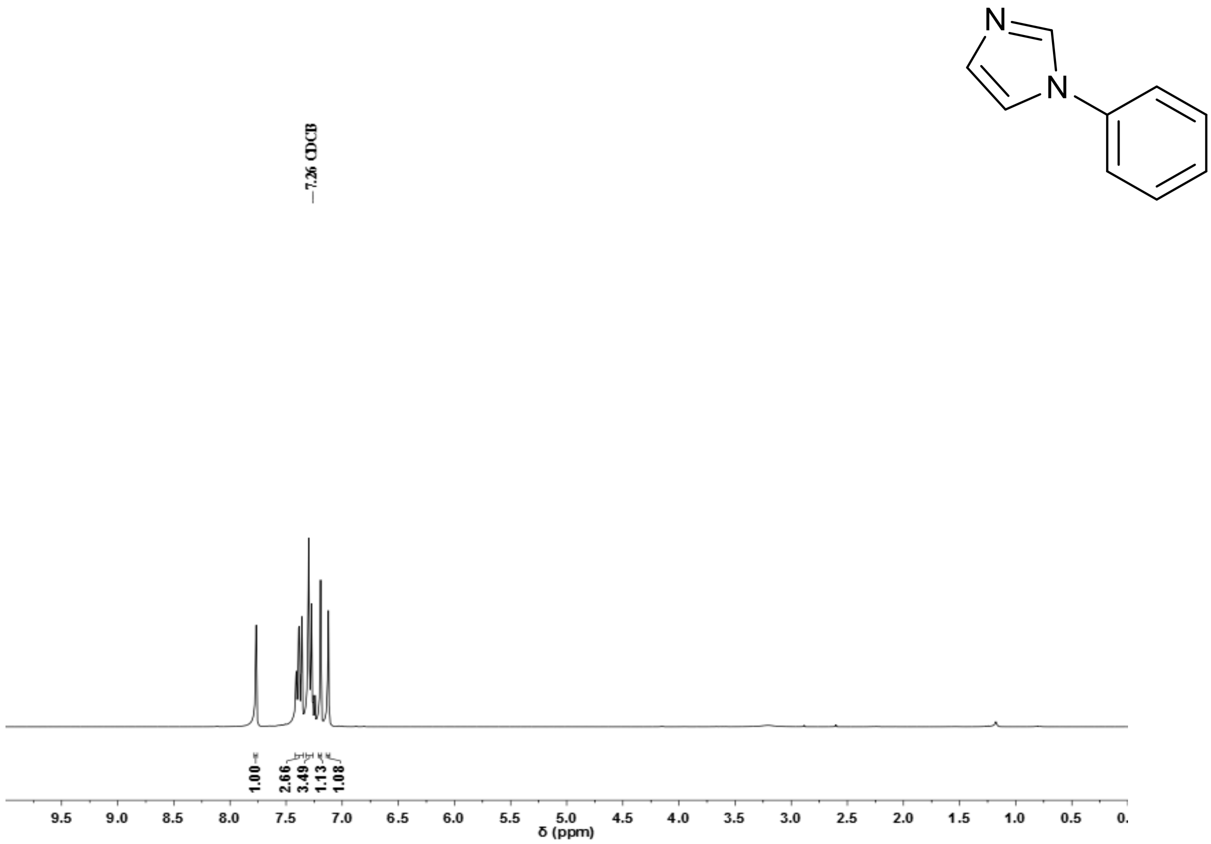

Figure S42. ^{1}H NMR spectrum of 1-phenylimidazole.

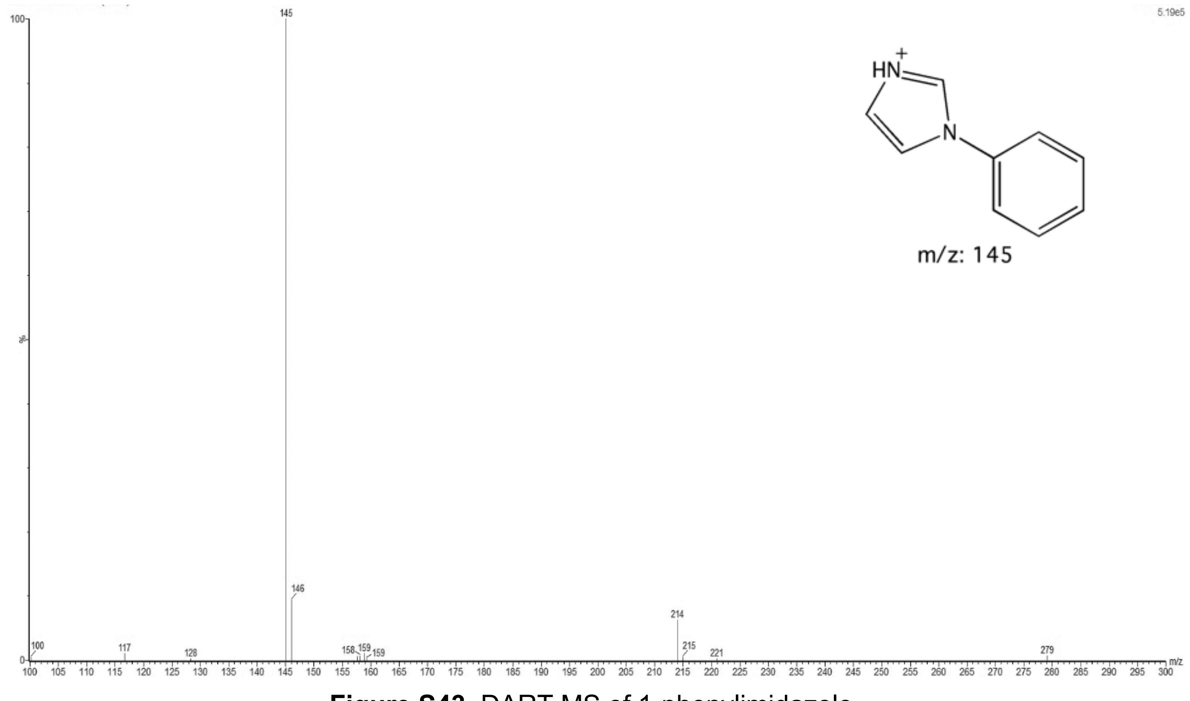

Figure S43. DART MS of 1-phenylimidazole.

S5. Computational Investigations

S5.1 Computational Methodology

S5.1.1 Free Energy Calculation

Harmonic frequency calculations were performed on the optimized structures to verify that they are indeed energy minima. The Goodvibes python script[5] was used to obtain the zero-point energy and thermal corrections for the selected structures, by calculating an approximate partition function using the rigid-rotator-quasiharmonic oscillator. To improve accuracy, the frequencies were scaled using the scaling factor 1.0340.[6] Frequencies below 100 cm^{-1} were set to 100 cm^{-1} and the gas phase values were corrected to a solution-phase standard state of 1 mol L^{-1}. Reported energies are either relative electronic energies ΔE (PBE0/def2-TZVP/D3/COSMO//PBE0/def2-SVP/D3/-COSMO) or relative free energies ΔG at 298.15 K, all given in kJ mol^{-1}.

S5.1.2 Molecular Dynamics Simulations

Structures were equilibrated using a modified procedure by Wallnöfer *at al.*[7] that involves extensive heating and cooling to achieve equilibration. The production runs (in explicit chloroform) were carried out in NpT ensembles at 300 K, using Amber18.[8] Temperature was regulated with the Langevin thermostat,[9] whereas the pressure was kept at 1 bar using the Berendsen barostat.[10] The SHAKE algorithm[11] was used to restrain hydrogen bonds allowing for a time step of 2 fs; coordinates were saved every 10 ps, simulating a total of 1 µs. Accelerated MD (aMD) simulations[12] were carried out using the dual-boost algorithm implemented in Amber18,[8] where a bias was applied on the total potential and an additional boost on the dihedral term.[8] A total of 12 aMD simulations, each with 1 µs, were performed with various boosting parameters. These settings were derived as proposed by Pierce *et al.*[13] by performing 100 ns classical MD simulations. All trajectories of the production runs were combined, and the structures were aligned on the phenanthroyl bridge. A hierarchical

clustering was applied on the combined trajectories to obtain a structurally divers ensemble. We did not re-weight the trajectories because (i) accurate re-weighting is difficult to achieve within aMD and energy minima remain such regardless of their relative energies, (ii) our goal was to obtain structures that are as diverse as possible, since relative energies of the conformers will change upon Cu(I) coordination.

S5.2 Computational protocol to determine the dissociation transition state $\mathbf{TS_{3\text{-}4}}$

The transition state $\mathbf{TS_{3\text{-}4}}$ presents a particular difficulty in obtaining a valid structure, as the electronic energy is rising in a monotonic fashion, as the dissociation coordinate is elongated. While this makes the determination of the exact structure difficult, the energy barrier of this TS can still be estimated. By manually creating structures along the reaction coordinate, 0.25 Å apart, and performing a restrained optimization as well as frequency calculations, the initial steps of the reaction can be characterized (Figure S44). When another datapoint is added, with the product structures infinitely separated, a jump in free energy can be observed. By fitting a sigmoid function on the values of $-T\Delta S$ (black dotted line), the free energy along the dissociation (red line) can be estimated from adding the calculated enthalpy (blue dotted line) to $-T\Delta S$ (black dotted line). The highest point is taken as the estimated for $\mathbf{TS_{3\text{-}4}}$. The approximated transition-state structure is depicted in Figure S50.

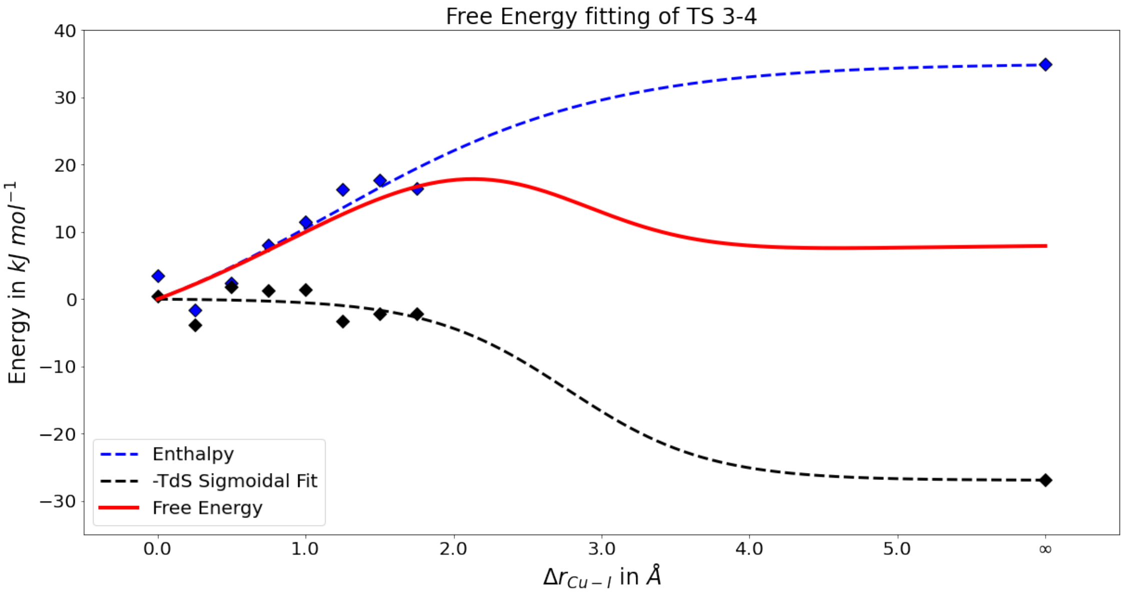

Figure S44. Free energy fitting of **TS_{3-4}**. Structures were generated along the reaction coordinate in steps of 0.25 Å (blue dots) and interpolated by a sigmoid blue dashed line. Optimizations and frequency calculations were carried out at the PBE0/def2-TZVP/D3/COSMO//PBE0/def2-SVP/D3/COSMO level (black dots) and sigmoid functions were fitted for the enthalpy and -TΔS (black dashed line). The free energy was calculated by summing up the sigmoids and the maximum value was taken as the TS free energy estimate (red line).

S5.3 Structures of reactive species

The transition states **TS$_{2-3}$** (proton abstraction) and **TS$_{4-5}$** (iodide abstraction) are diffusion controlled, hence, a barrier of 20 kJ/mol is assumed and no structures are reported.

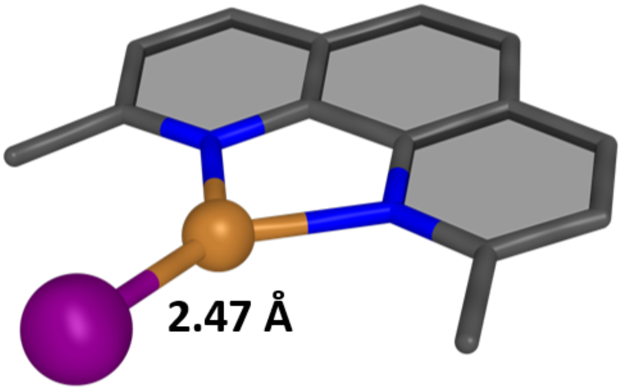

Figure S45. Structure **1**, optimized with PBE0/def2-SVP/D3/COSMO(toluene).

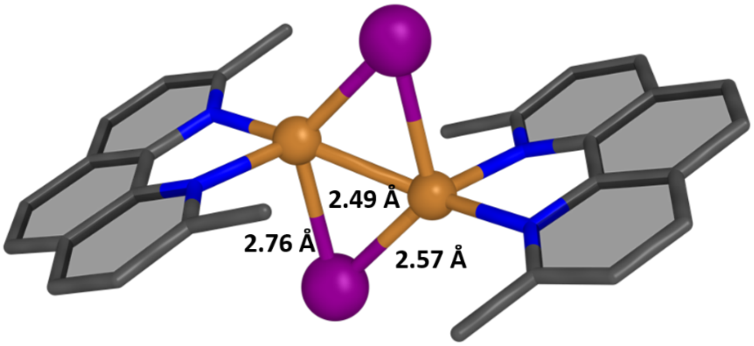

Figure S46. Structure [**Cu(Phen)(μ-I)**]$_2$ optimized with PBE0/def2-SVP/D3/COSMO(toluene).

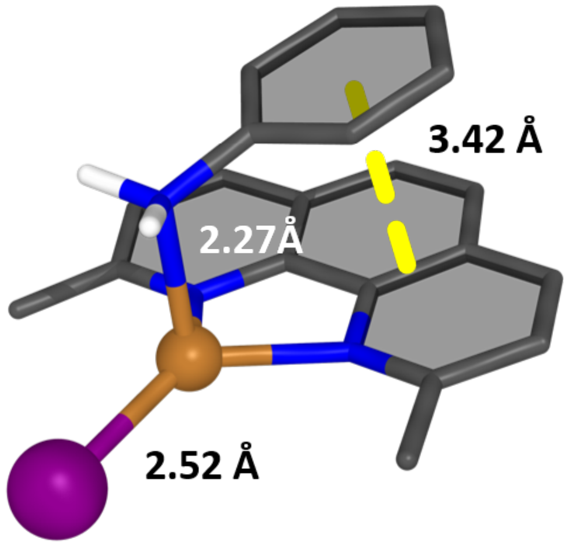

Figure S47. Structure **2**, optimized with PBE0/def2-SVP/D3/COSMO(toluene).

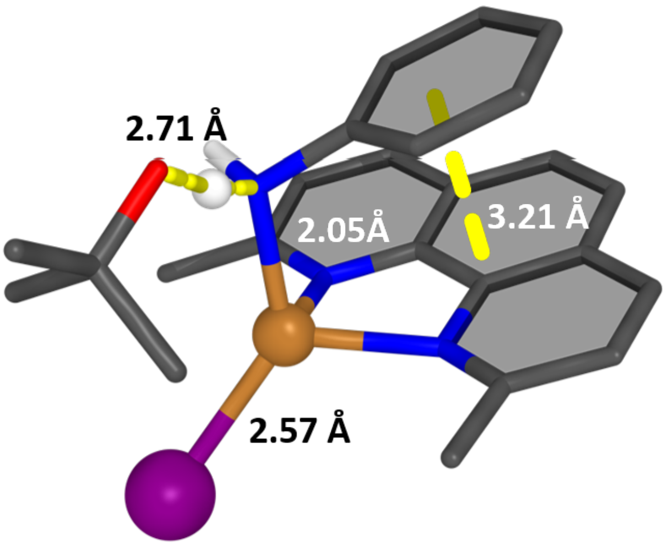

Figure S48. Structure of approximate $\mathbf{TS}_{2\text{-}3}$, optimized with PBE0/def2-SVP/D3/COSMO(toluene).

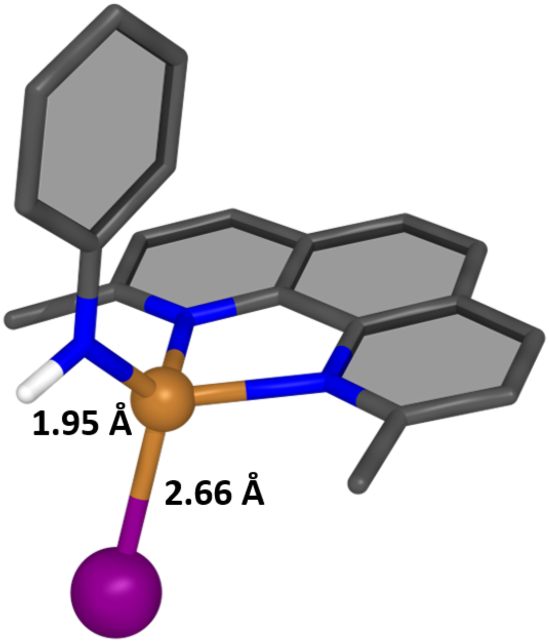

Figure S49. Structure **3**, optimized with PBE0/def2-SVP/D3/COSMO(toluene).

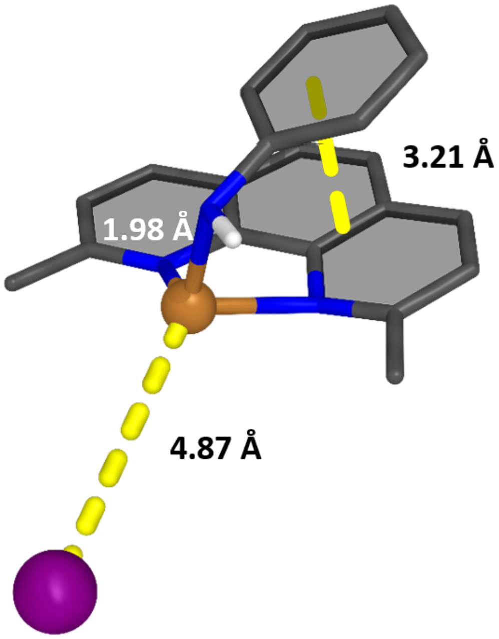

Figure S50. Structure **TS$_{3\text{-}4}$**, Estimate optimized with PBE0/def2-SVP/D3/COSMO(toluene).

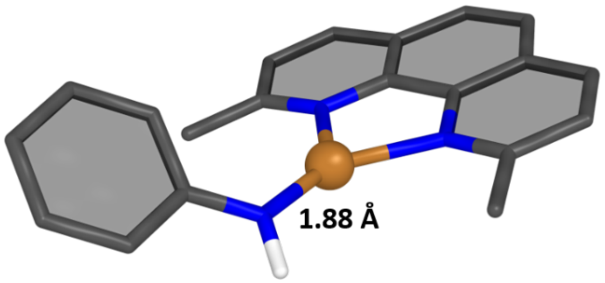

Figure S51. Structure **4**, optimized with PBE0/def2-SVP/D3/COSMO(toluene).

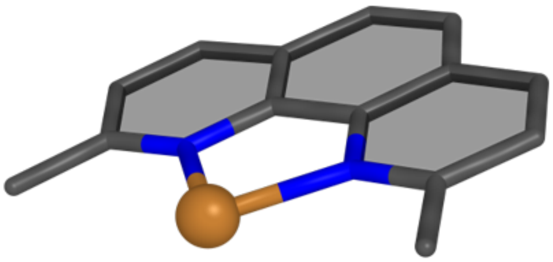

Figure S52. Structure **2a** optimized with PBE0/def2-SVP/D3/COSMO(toluene), counter ion is omitted.

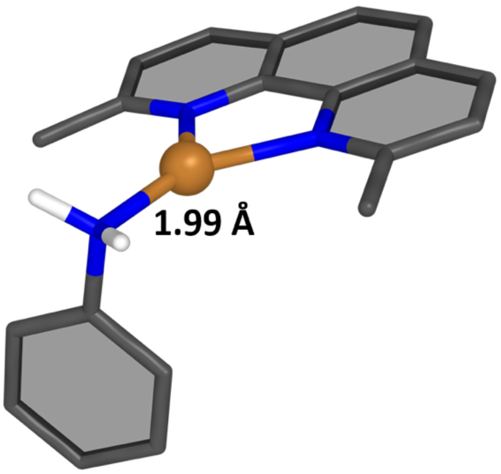

Figure S53. Structure **3a** optimized with PBE0/def2-SVP/D3/COSMO(toluene), counter ion is omitted

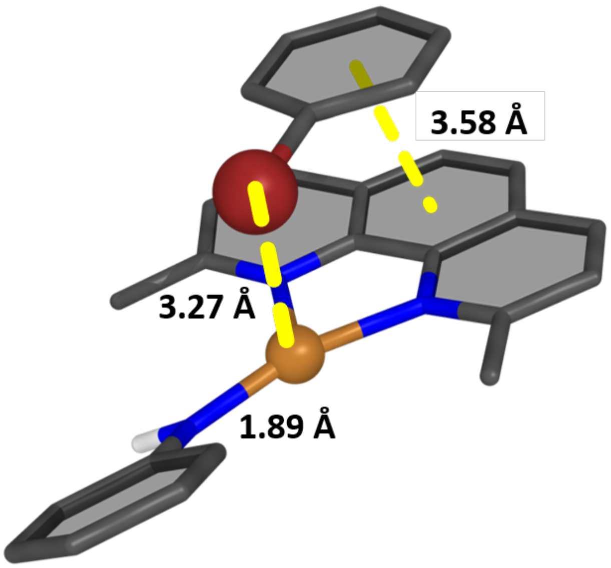

Figure S54. Structure **5**, optimized with PBE0/def2-SVP/D3/COSMO(toluene).

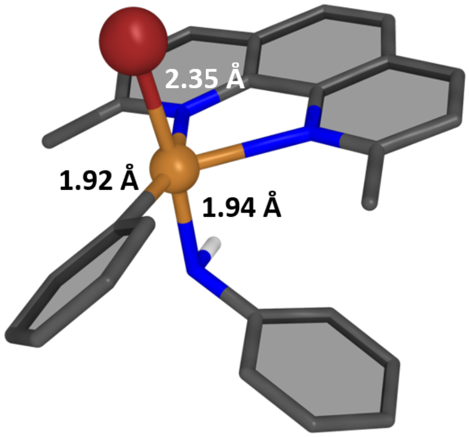

Figure S55. Structure **6**, optimized with PBE0/def2-SVP/D3/COSMO(toluene).

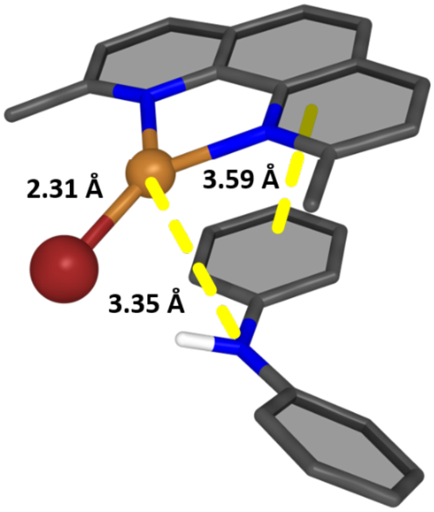

Figure S56. Structure **7**, optimized with PBE0/def2-SVP/D3/COSMO(toluene).

S5.4 Subsequent cycles of the reaction

The first cycle of the reaction begins with iodine and ends with bromine attached to the Cu atom. All further cycles of the reaction take place starting with the Br analogue of structure. An investigation of the electronic energy path of the Br equivalents of structures **1** to **4** is shown in Figure S57. The reaction energies are largely similar, with the main differences being in the case of **3** and **4** with the bromine intermediates being 7 and 20 kJ mol^{-1} higher in energy.

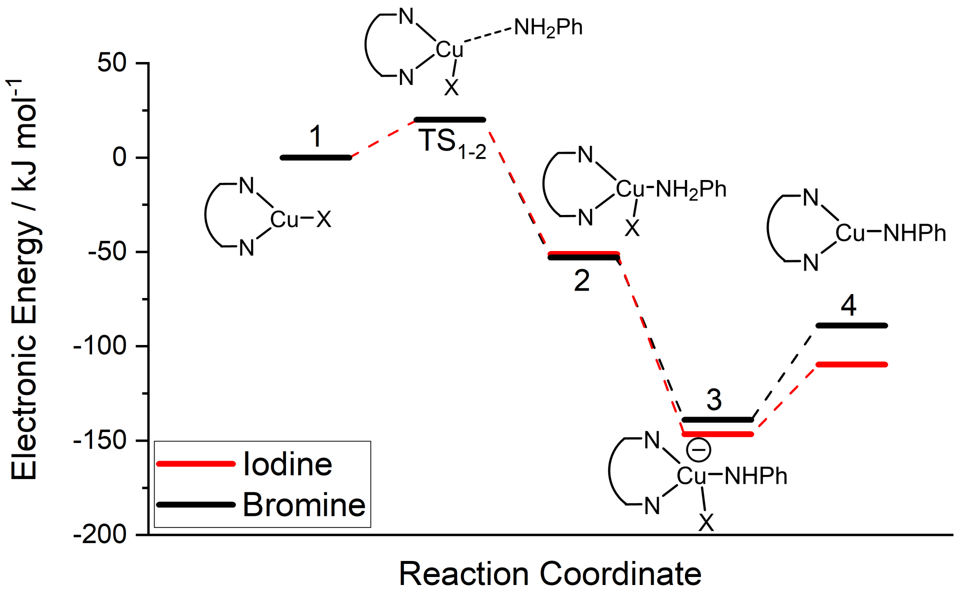

Figure S57. Energy diagram of structures **1**-**4** for the first (iodine) and subsequent (bromine) cycles of the reaction. X stands for the respective halogen atom. Energies were computed with PBE0/def2-TZVP/D3/COSMO//PBE0/def2-SVP/D3/COSMO.

S5.5 Investigation of the supramolecular complexe

The supramolecular catalyst is a difficult structure to fully investigate, as QM calculations are computationally intensive and do not take into account the flexibility of the calixarene ring. MD simulations can not only describe the flexibility of the cage, but they allow for the inclusion of explicit solvent, which is essential for preventing the calixarene cavity from collapsing. This comes at the cost of an inability to take into account any bond formation or dissociation. To this end, we developed a protocol, as shown in Figure S58, where we combine MD simulations with QM optimizations, in such a way that we are able to obtain snapshots along the reaction pathway. By utilizing DFT for elucidating the reaction path on a model system, we can pinpoint the important intermediates involved in the reaction. Meanwhile, on the MD side, we can perform simulations of the catalyst to get an idea about the cage movements. By clustering the simulations, we are able to acquire a few representative structures, to which we can fit the various intermediates. These structures can then subsequently serve as starting points for more simulations of the various intermediates, as well as constructing a reaction profile for the whole supramolecular catalyst – being able to capture the effect on confinement on the reaction path. Specificities of each calculation step can be found in the respective subsections of the computational methodology.

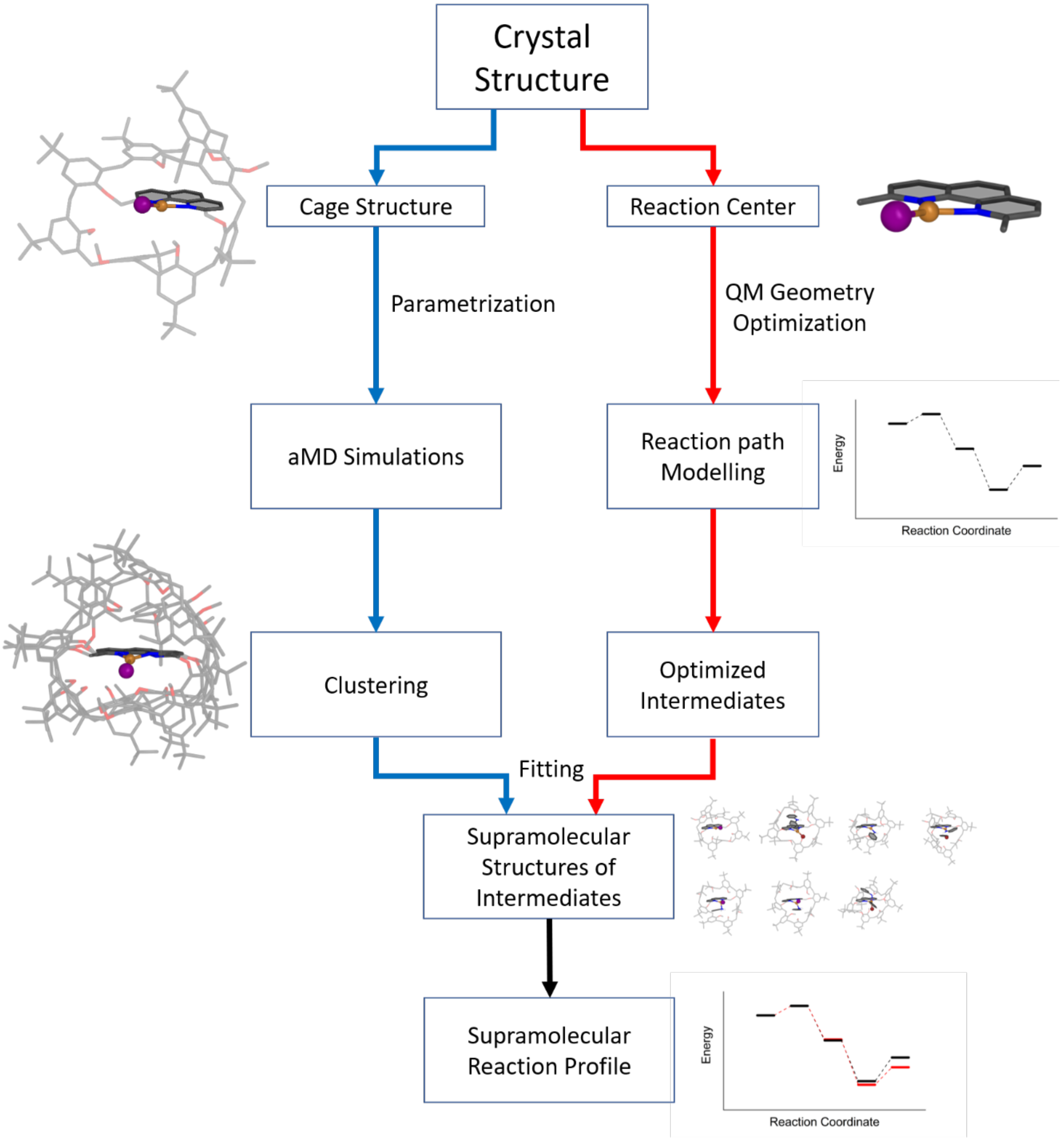

Figure S58. Workflow combining QM and MD simulations, developed to investigate the supramolecular catalyst.

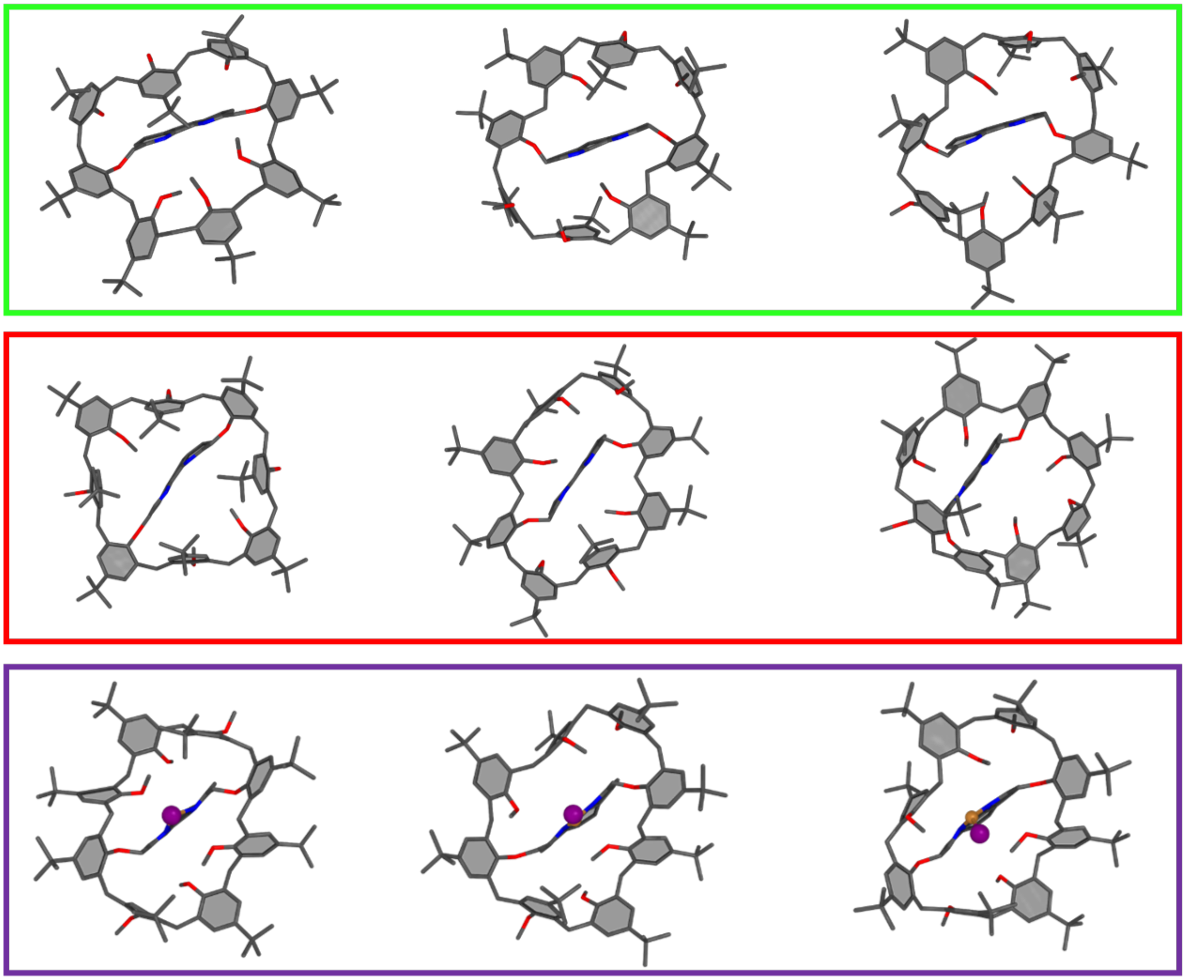

Figure S59. Top: Three most populated clusters of the aMD simulations of the calixarene ligand without Cu coordination (**C_8PhenMe$_6$**) in toluene. Middle: most populated clusters of **C_8PhenMe$_6$** in chloroform. Bottom: Most populated the clusters for the simulation of **1_{calix}** in explicit chloroform.

The fitting of the small phenanthroyl system was performed on multiple clusters from different simulations. Clustering the aMD simulations provided with an overview of the dynamics of the calixarene macrocycle. When comparing the main clusters in the simulations with implicit solvent (not shown) and chloroform, it was observed that explicit solvation was essential to retain the cavity. When comparing the top three most populated clusters of the aMD simulations of the uncoordinated ligand **C_8PhenMe$_6$** in toluene and in

chloroform (top and middle panel in Figure S59), it is evident that the structures are very similar. Indeed, even CuI coordination yielding $\mathbf{1}_{calix}$ (bottom panel in Figure S59) did not change much, but very similar clusters were obtained for the simulation of $\mathbf{1}_{calix}$ in chloroform compared to simulation of the ligand alone with cavities of similar size. As results for chloroform and toluene were similar, all subsequent calculations were performed in chloroform only to reduce computational costs.

As the simulations yielded well-defined cavities, fitting of structures **2** to **7** deemed possible without too many clashes. To this end, structures **2** through **7** were fitted into the cavity, using the phenantroyl bridge as a base for alignment, yielding $\mathbf{2}_{calix}$ through $\mathbf{7}_{calix}$. Using the number of steric clashes as the criterion, each fitted structure was ranked. The most populated cluster of the simulation of $\mathbf{1}_{calix}$ showed the least amount of clashing and thus was chosen as the template for subsequent optimizations.

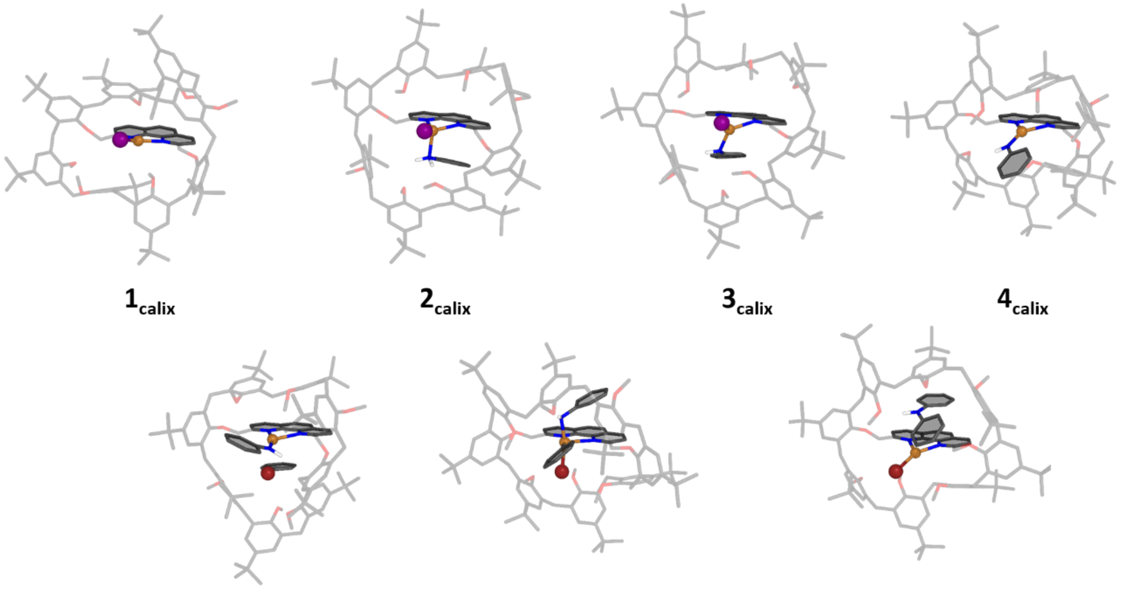

$\mathbf{1}_{calix}$ $\mathbf{2}_{calix}$ $\mathbf{3}_{calix}$ $\mathbf{4}_{calix}$

$\mathbf{5}_{calix}$ $\mathbf{6}_{calix}$ $\mathbf{7}_{calix}$

Figure S60. Structures of the supramolecular calixarene intermediates, optimized with PBE0/def2-TZVP/D3/COSMO//PBE0/def2-SVP/D3/COSMO.

The resulting structures were subsequently optimized with PBE0/def2-SVP/D3+COSMO, followed by single point calculations with the larger def2-TZVP basis set and the energetic landscape was evaluated. Final structures are depicted in Figure S60.

As simulations of structure $\mathbf{1}_{calix}$ as well as $\mathbf{6}_{calix}$ were performed, the flexibility of the calixarene ring was also investigated. It was observed that the calixarene units exhibits less movement during the simulation of $\mathbf{6}_{calix}$. This was quantified as the relative entropy of the diherdrals between the individial units calculated with the X-entropy script.[14] In Figure S61, it can be seen that the distributions of the dihedrals in the simulated structure $\mathbf{6}_{calix}$ are considerably narrower than those of $\mathbf{1}_{calix}$.

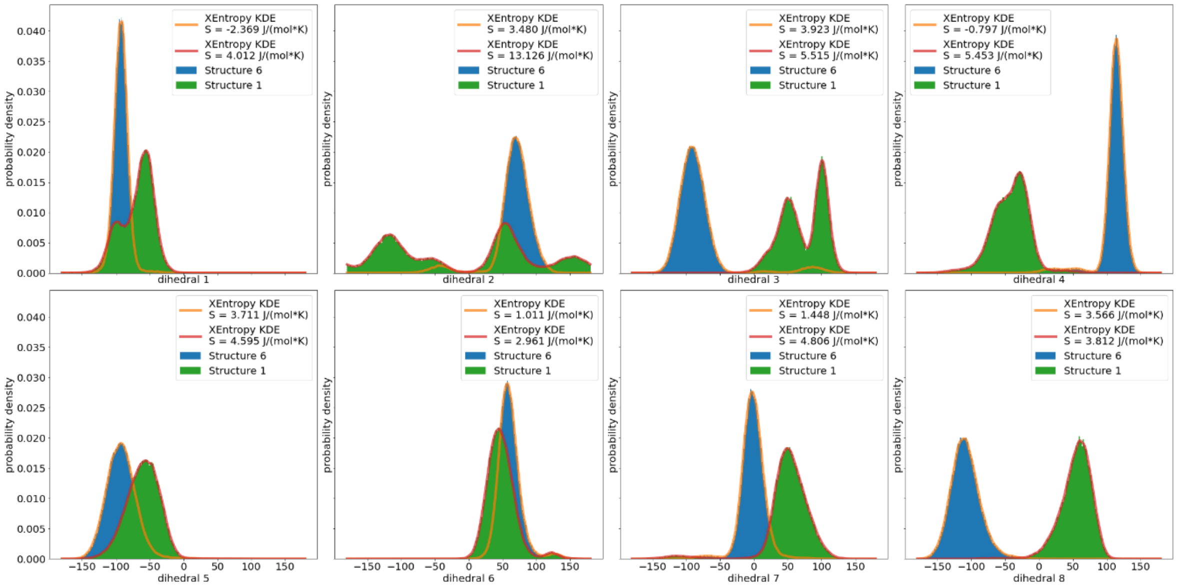

Figure S61. Entropy values of the dihedral distributions, as calculated with the X-entropy script. The green distribution, contoured by red, represents the values from the simulation of $\mathbf{1}_{\mathbf{calix}}$. The blue distribution, contoured by orange, represents the values obtained from the simulation of $\mathbf{6}_{\mathbf{calix}}$.

S6. References

(1) Armarego, W. L. F.; Chai, C. L. L. Purification of Organic Chemicals; **2009**.

(2) Hernández, D. J.; Vázquez-Lima, H.; Guadarrama, P.; Martínez-Otero, D.; Castillo, I. Tetrahedron Lett. **2013**, 54, 4930–4933.

(3) Munch, J. H.; C. D. Gutsche. p-tert-butylcalix[8]arene. Org. Synth. **1990**, 68, 243.

(4) Chandler, C. J.; Deady, L. W.; Reiss, J. A. J. Heterocyclic Chem. **1981**, 18, 599.

(5) Luchini G.; Alegre-Requena J. V.; Funes-Ardoiz I.; Paton R. S. GoodVibes: automated thermochemistry for heterogeneous computational chemistry data. F1000Research **2020**, 291.

(6) Kesharwani, M.; Brauer, B.; Martin, L. Frequency and Zero-Point Vibrational Energy Scale Factors for Double-Hybrid Density Functionals (and Other Selected Methods): Can Anharmonic Force Fields Be Avoided? J. Phys. Chem. A **2015**, 119, 1701–1714.

(7) Wallnoefer, H; Handschuh, S.; Liedl, K.; Fox, T. Stabilizing of a Globular Protein by a Highly Complex Water Network: A Molecular Dynamics Simulation Study on Factor Xa. J. Phys. Chem. B. **2010**, 114, 7405–7412.

(8) a) Salomon-Ferrer, R.; Case, D.; Walker, R. An overview of the Amber biomolecular simulation package. Wiley Interdiscip. Rev. Comput. Mol. Sci. **2013**, 3, 198–210. b) D. A. Case, I. Y. Ben-Shalom, S. R. Brozell, D. S. Cerutti, T. E. Cheatham, III, V. W. D. Cruzeiro, T. A. Darden, R .E. Duke, D. Ghoreishi, M. K. Gilson, H. Gohlke, A.W. Goetz, D. Greene, R. Harris, N. Homeyer, Y. Huang, S. Izadi, A. Kovalenko, T. Kurtzman, T. S. Lee, S. LeGrand, P. Li, C. Lin, J. Liu, T. Luchko, R. Luo, D. J. Mermelstein, K. M. Merz, Y. Miao, G. Monard, C. Nguyen, H. Nguyen, I. Omelyan, A. Onufriev, F. Pan, R. Qi, D. R. Roe, A. Roitberg, C. Sagui, S. Schott-Verdugo, J. Shen, C. L. Simmerling, J. Smith, R. Salomon-Ferrer, J. Swails, R. C. Walker, J. Wang, H. Wei, R. M. Wolf, X. Wu, L. Xiao, D. M. York and P. A. Kollman. AMBER 2018, Reference Manual. University of California, San Francisco. **2018**.

(9) Adelman, S.; Doll, J. Generalized Langevin Equation Approach for Atom-Solid-Surface Scattering - General Formulation for Classical Scattering off Harmonic Solids. J. Chem. Phys. **1976**, 64, 2375–2388.

(10) Berendsen, H.; Postma, J.; van Gunsteren, W.; DiNola, A.; Haak, J. Molecular-Dynamics With Coupling to an External Bath. J. Chem. Phys. **1984**, 81, 3684–3690.

(11) Ciccotti, G.; Ryckaert, J. Molecular-Dynamics Simulation of Rigid Molecules. Comput. Phys. Commun. **1986**, 4, 345-392.

(12) a) Hamelberg, D.; de Oliveira, C.; McCammon, J. Sampling of slow diffusive conformational transitions with accelerated molecular dynamics. J. Chem. Phys. 2007, 127, 1-9. b) Hamelberg, D.; Mongan, J.; McCammon, J. Accelerated molecular dynamics: A promising and efficient simulation method for biomolecules. J. Chem. Phys. **2004**, 120, 11919–11929.

(13) Pierce, L.; Salomon-Ferrer, R.; F. de Oliveira, C.; McCammon, J.; Walker, R. Routine Access to Millisecond Time Scale Events with Accelerated Molecular Dynamics. J. Chem. Theory Comput. **2012**, 8, 2997–3002.

(14) Kraml, J.; Hofer, F.; Quoika, P. K.; Kamenik, A. S.; Liedl, K. R. *J. Chem. Inf. Model.* **2021**, *61*, 1533–1538.
